# Genomic divergence of zebu and taurine cattle identified through high-density SNP genotyping

**DOI:** 10.1186/1471-2164-14-876

**Published:** 2013-12-13

**Authors:** Laercio R Porto-Neto, Tad S Sonstegard, George E Liu, Derek M Bickhart, Marcos VB Da Silva, Marco A Machado, Yuri T Utsunomiya, Jose F Garcia, Cedric Gondro, Curtis P Van Tassell

**Affiliations:** Animal Genetics Laboratory, The University of Queensland, School of Veterinary Science, Gatton, QLD 4343 Australia; School of Environmental and Rural Science, University of New England, Armidale, NSW 2351 Australia; United States Department of Agriculture, Agricultural Research Service, Bovine Functional Genomics Laboratory, Beltsville, MD 20705 USA; Universidade Estadual Paulista (UNESP), Rua Clovis Pestana 793, Aracatuba, SP, Brazil; Bioinformatics and Animal Genomics Laboratory, Embrapa Dairy Cattle, Juiz de Fora, Minas, Gerais, Brazil; CSIRO Food Futures Flagship, and Division of Animal, Food and Health Sciences, Queensland Bioscience Precinct, Brisbane, QLD 4067 Australia

**Keywords:** Bos, Taurus, Indicus, FST, Selection, Speciation

## Abstract

**Background:**

Natural selection has molded evolution across all taxa. At an arguable date of around 330,000 years ago there were already at least two different types of cattle that became ancestors of nearly all modern cattle, the *Bos taurus taurus* more adapted to temperate climates and the tropically adapted *Bos taurus indicus*. After domestication, human selection exponentially intensified these differences. To better understand the genetic differences between these subspecies and detect genomic regions potentially under divergent selection, animals from the International Bovine HapMap Experiment were genotyped for over 770,000 SNP across the genome and compared using smoothed F_ST_. The taurine sample was represented by ten breeds and the contrasting zebu cohort by three breeds.

**Results:**

Each cattle group evidenced similar numbers of polymorphic markers well distributed across the genome. Principal components analyses and unsupervised clustering confirmed the well-characterized main division of domestic cattle. The top 1% smoothed F_ST_, potentially associated to positive selection, contained 48 genomic regions across 17 chromosomes. Nearly half of the top F_ST_ signals (n = 22) were previously detected using a lower density SNP assay. Amongst the strongest signals were the BTA7:~50 Mb and BTA14:~25 Mb; both regions harboring candidate genes and different patterns of linkage disequilibrium that potentially represent intrinsic differences between cattle types. The bottom 1% of the smoothed F_ST_ values, potentially associated to balancing selection, included 24 regions across 13 chromosomes. These regions often overlap with copy number variants, including the highly variable region at BTA23:~24 Mb that harbors a large number of MHC genes. Under these regions, 318 unique Ensembl genes are annotated with a significant overrepresentation of immune related pathways.

**Conclusions:**

Genomic regions that are potentially linked to purifying or balancing selection processes in domestic cattle were identified. These regions are of particular interest to understand the natural and human selective pressures to which these subspecies were exposed to and how the genetic background of these populations evolved in response to environmental challenges and human manipulation.

**Electronic supplementary material:**

The online version of this article (doi:10.1186/1471-2164-14-876) contains supplementary material, which is available to authorized users.

## Background

Natural selection has shaped the genome of all living creatures in our planet, including domesticated animals. Nearly all modern cattle can be associated with one of two types or sub-species. This division between the types *Bos taurus taurus* (taurine cattle) and *Bos taurus indicus* (zebu cattle) is estimated to have occurred from a common ancestor between 330,000 [1] and 2 million [2] years ago. Since divergence, cattle types have accumulated different genetic variations, which have contributed to highly differentiated phenotypes. It is assumed that the divergence between cattle types was long before domestication, which is estimated to have occurred between 10,000 to 7,000 BC in two separate locations: the Fertile Crescent (taurine cattle) and the Indus Valley (zebu cattle) [3, 4]. After domestication human-oriented selection added further complexity to the evolution of cattle.

For most of the history of human-cattle coexistence the environment was the main force driving changes in the animals’ genome. Shortly after domestication, human breeders preferred traits that enabled easy management; however, breeders also sought production improvement traits as well [5]. The introduction of the concept of *breed* in the 19th century led to human-oriented selection imposing strong bottlenecks, which created population demes based on phenotypes. Breed formation was followed by breed expansion via the use of artificial insemination, which reduced genetic variability within breeds particularly in the sex chromosomes and mitochondrial DNA [6]. This is due to the fact that only one haplotype is passed on to the following generation, and subjected to stronger selective forces when compared to autosomal chromosomes.

Positive and balancing selections are terms used to characterize different aspects that selection forces might impose on a population. Positive selection, also termed directional or purifying selection, refers to the selection process through which a particular phenotype (or genotype) is favored in a given environment, which leads to an increase of its frequency in a population. In contrast, balancing selection refers to the selective process through which multiple alleles are selected, thus preserving the genetic diversity in a population. Balancing selection is often observed when heterozygous animals have a competitive advantage. Alternatively, these may be regions of convergent selection across groups. Importantly, both positive and balancing selection phenomena can be tracked using SNP genotypes or sequence data from the cattle genome.

SNP genotyping has become widely used in animal genetics and a number of methods have been developed to identify regions under selection. Out of these F_ST_ is a widely used statistic to evaluate the diversity of subpopulations of animals or to determine the relative distance between populations. Many variations of the F_ST_ concept [7] exist, but all adhere to the core principle of being a metric of allele frequencies and their variance. This metric has also been used to identify loci under selection [8–10].

In this study, we used a pure drift F_ST_ model [11] which assumes all animals originated from the same ancestral population. This model was applied to taurine and zebu animals to identify loci under selection. These two groups correspond to the main (and most ancestral) separation of domestic cattle, which in most but not all cases corresponds to animals adapted to tropical and temperate environments. The identification of such loci can aid in the identification of genes and genomic variants that are related to environmental adaptation and/or selection derived from human agro-pastoral activities.

## Methods

### Statement on the ethical use of animals

No ethics statement was required for the collection of genetic material. The DNA from animals included in this study were either part of previous analyses that obtained specific permissions [12] or were extracted from semen straws collected in accredited AI centers in accordance with the Brazilian legislation on animal welfare.

### Cattle samples and SNP genotypes

All individuals were genotyped using the BovineHD BeadChip that includes ~777 K SNP (Illumina, Inc. San Diego, USA) following standard procedures. The SNP set included in this genotyping platform was carefully selected to reduce the potential for ascertainment bias during SNP discovery. Seven different grouping of breeds were used to assess the minor allele frequency of all available SNP, this included Holstein, Angus, Nelore, *Bos taurus taurus* dairy excluding Holstein, *Bos taurus taurus* beef ignoring Angus, *Bos taurus indicus* excluding Nelore, and adapted *Bos taurus taurus* (e.g. Senepol). This was complemented with sequence data from 30 breeds that were compiled and weighted to minimize ascertainment bias. More information on the BovineHD can be found in the supplier’s website (http://www.illumina.com/documents//products/datasheets/datasheet_bovineHD.pdf).

Only animals with call rates > = 98%, and SNP with more than 95% successful genotypes were kept in the final dataset. Filtering was also based on available pedigree information and the estimated proportion of alleles shared identical-by-descent (PI_HAT > 0.8) ([13]http://pngu.mgh.harvard.edu/~purcell/plink/), animals with high relatedness were excluded. A total of 339 *Bos taurus taurus* or taurine individuals from the Bovine Hapmap DNA panel [12] were included in the analyses. Breeds represented in this group were: Angus (n = 44), Brown Swiss (n = 24), Charolais (n = 37), Guernsey (n = 21), Hereford (n = 36), Holstein (n = 63), Jersey (n = 39), Limousin (n = 47), Norwegian Red (n = 17), and Red Angus (n = 11). The *Bos taurus indicus* or Zebu animals (n = 166) were also from the Bovine Hapmap experiment, and they were complemented with additional individuals. Breeds represented in this group were: Nelore (n = 91), Gir (n = 50), and Guzera (n = 25). Even though Brahmans are considered zebu animals, it is known that taurine animals were also used during the breed formation and expansion; therefore they were not included in these analyses.

### Population and linkage disequilibrium (LD) structure

Pairs of markers with high linkage disequilibrium (LD) provide redundant information and impose higher computational demands for population structure analyses. To remove extraneous information, the dataset was pruned based on LD between markers using the PLINK [13] command --indep-pairwise 50 10 0.1, which calculates LD for each pair of marker in a window of 50 SNP. If a pair of SNP had r^2^ > 0.1, then one of the SNP was removed, the window was moved 10 SNP and the process restarted. The pruned genotypes defined a dataset including 38,681 SNP that were then used to assess the population structure using three methods: 1) unsupervised clustering of individuals based on maximum likelihood as implemented in the program Admixture Version 1.20 [14] with cluster number (K) equal 2; 2) principal components analysis as implemented in GCTA [15]; and 3) estimated genetic relationship matrix [16] visualized as heat map using R [17]. For plots of LD between markers, r^2^ were calculated using Haploview [18].

### Identification of genomic regions under selection

F_ST_ statistics were used to characterize the differentiation between taurine and zebu animals by first identifying SNP potentially under selection. Next, genomic regions with a high proportion of such SNP were identified, and then the genic content of regions with extreme signals for positive and balancing selection were further analyzed. The estimation of SNP F_ST_ was based on a pure drift model defined by Nicholson et al. [11], following the simplification proposed by Flori et al. [10]. These analyses were performed using R [17] scripts. The SNP F_ST_ were smoothed across the Bovine genome reference assembly UMD 3.1 [19] using a local variable bandwidth kernel estimator [20] (R package *lokern*), where every fifteen SNP F_ST_ values generated one smoothed F_ST_ value. This bandwidth was used because it covers a region of ~50Kb which is the average extent of LD found in these populations. The genomic regions with predominantly higher F_ST_ values usually resulted in high values of smoothed F_ST_ and were potentially associated to positive selection. In contrast, regions with mainly low F_ST_ values generated low smoothed F_ST_ values and were potentially associated to regions under balancing selection. The top and bottom 1% smoothed F_ST_ values were identified, translated into genomic position (UMD 3.1) and the genic content of each region was tested for gene ontology overrepresentation. The cattle chromosome X (BTAX) is highly differentiated between taurine and zebu animals. Therefore, the identification of the top and bottom 1% values included only the autosomes, being the BTAX analyzed separately as it contains regions under relatively strong positive selection. Similar analyses were also performed only within-taurine (n = 9 breeds, the Red Angus was excluded due to small sample size) and only within-zebu (n = 3 breeds). These analyses were performed to gather hints as to the origin of the differentially selected regions seen between zebu and taurine cattle.

Regions harboring copy number variants (CNV) might also be under selection and contributing to an observed selection signal, therefore CNV regions that coincide to smoothed F_ST_ peaks were further explored. Gene content of cattle CNV regions was assessed using Ensembl (ftp://ftp.ensembl.org/pub/current_fasta/bos_taurus/pep/). It is worthwhile to point out that F_ST_ and CNV results did not use the exact same samples. CNV results are based on Bickhart *et al*. [21] that use a Holstein, a Nelore, a Hereford and 3 Angus samples, also included in the F_ST_ analyses. Intersections between balancing selection region coordinates and exon positions were compared using MySQL queries. We obtained a catalog of all bovine peptides from Ensembl. This yielded 22,118 peptides, 345 of which overlap with 24 predicted balancing selection regions, and corresponded to 318 unique Ensembl genes. Using PANTHER version 7 [22], we tested for over representation of biological process, molecular function and pathway terms under the balancing selection regions. Results were Bonferroni [23] adjusted and PANTHER terms with less than five observations were not further analyzed. Similar analyses were performed on the peptides under the 48 positive selection regions. PANTHER results were similar when all peptides under the 24 balancing selection regions and 48 positive selection regions were combined in a single analysis.

## Results

### SNP genotypes

**Figure 1:**
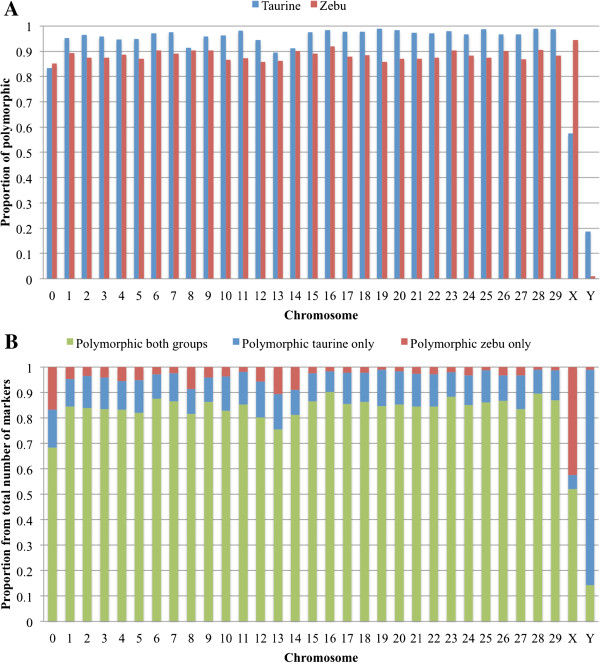
**Polymorphic status of the BovineHD (Illumina) markers in zebu and taurine cattle. A)** Proportion of polymorphic markers, and **B)** Proportion of markers by polymorphic status across both cattle types.

After quality control, a total of 768,506 SNP were considered. In taurine, most of the autosomes had >90% of markers polymorphic and in zebu slightly less markers were polymorphic (between 80-90%). This distribution was similar across all autosomes; however, the taurine group had a reduced proportion of polymorphic markers when compared to the zebu on BTAX (Figure 1A). Most autosomes had >80% of SNP polymorphic in both groups, with ~10% polymorphic only in taurine and only a reduced number of SNP exclusively polymorphic in zebu. The zebu exclusive SNP group was different again for the BTAX where ~50% of the SNP were polymorphic in both groups and close to 40% polymorphic only in zebu (Figure 1B). Within cattle types, the average heterozygosity was 0.21 and 0.29 for zebu and taurine.

### Population substructure

**Figure 2:**
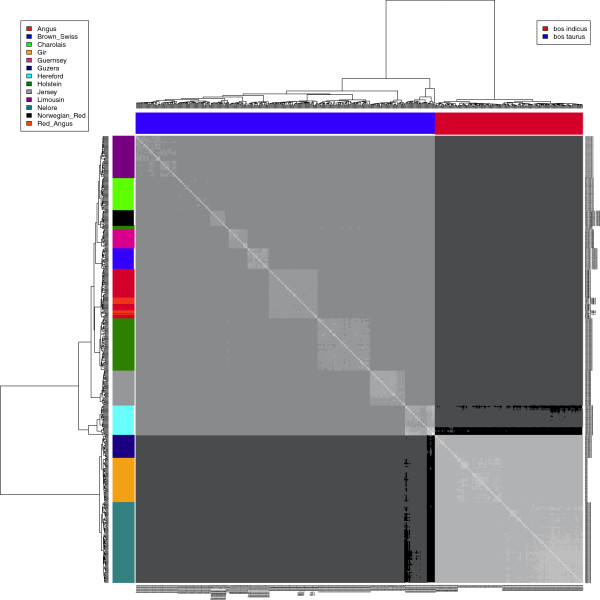
**Heatmap of relationship between individuals of 10 taurine and 3 zebu cattle breed (n = 505) based on the genetic relationship matrix calculated using 768,506 SNP genotypes.**

The separation between taurine and zebu is the most substantial type-distinction between domestic cattle. Clustering animals based on the genetic relationship matrix clearly demonstrates this division between cattle populations (Figure 2), which is also seen using an unsupervised clustering with selected number of clusters K = 2 (Additional file 1: Figure S1A). This latter analysis evidences the majority of individuals are pure bred within each cattle type assigning an estimated proportion of more than 0.9 for either the zebu or taurine clusters.

The first principal component, which is the axis that explains the most variance, not surprisingly corresponds to the same main division. The second principal component starts to subdivide the taurine animals (Additional file 1: Figure S1B and C). This subdivision of taurine animals was also seen in four independent runs of principal components analyses that used the same number of individuals per breed and different random combinations of taurine breeds in addition to the three zebu breeds (Additional file 2: Figure S2). This agrees with the lower pair-wise F_ST_ observed between zebu breeds in comparison to taurine breeds (Additional file 3: Table S1).

### Genomic regions under selection

**Figure 3:**
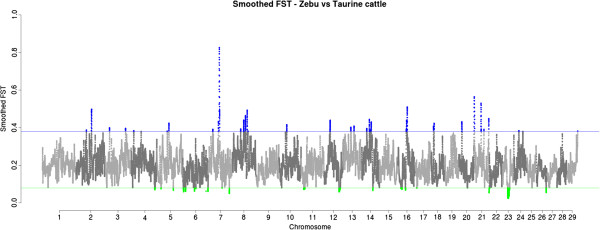
**Smoothed FST comparing taurine and zebu animals.** Only autosomes are plotted in alternated shades of gray. The top and bottom 1% values are highlighted in blue and green, corresponding to the regions under positive and balancing selections.

Regions under positive and balancing selection were defined as the regions in the top and bottom 1% of all smoothed F_ST_ values, respectively (Figure 3, Tables 1 and 2).

**Table 1:** **Positive selection: regions in the top 1**% **smoothed F**_**ST**_ **values**

Region	BTA	SNP start pos	SNP end pos	Highest sFST	CNV [21]	Within cattle type**	Candidate genes	Cross reference
P1	2	47,857,335	48,065,161	0.387	1			
P2*	2	71,565,086	72,885,823	0.498	1	Hol		[24]
P3	3	19,689,648	20,166,059	0.399	0	Hol, Nor, Bro	CDC42SE1	
P4	3	94,742,479	95,401,345	0.396	0			
P5	4	12,106,878	12,361,811	0.384	0			
P6	4	46,670,940	46,814,875	0.380	0			[25]
P7	5	48,229,556	48,336,996	0.381	0			[9, 12, 24]
P8	5	55,881,766	56,801,729	0.424	1	Hol	STAT6, GLI1	[24, 25]
P9	7	21,008,805	21,606,667	0.391	0	Gue	ITGB1BP3	[24, 25]
P10	7	47,299,497	47,859,329	0.433	3	Lim	SPOCK, PPP2CA	[12]
P11*	7	50,951,861	53,757,384	0.826	5	Ang, Cha, Gue, Nor, Gir	CD14, CDC23, EGR1, MYOT, TMEM173	[24]
P12	8	39,288,115	39,800,492	0.393	0		CD274	[24]
P13	8	53,490,845	54,592,381	0.440	1	Nor		[26]
P14	8	58,649,674	58,727,004	0.380	0			
P15	8	61,543,379	62,874,750	0.464	1			
P16	8	69,691,214	70,488,061	0.493	0	Ang	POLR3D, PPP3CC	
P17	8	73,617,355	73,704,634	0.382	0			
P18	10	36,488,829	37,051,537	0.416	0	Her	CHP	[25]
P19	12	27,935,604	29,508,940	0.439	1	Hol		[24, 25]
P20	13	34,119,211	35,054,048	0.402	0		ZEB1	
P21	13	48,893,096	49,816,619	0.408	0	Guz		[9]
P22	14	24,603,090	25,298,972	0.395	0	Nor	PLAG1, XKR4, MOS	[10]
P23	14	36,715,710	37,511,658	0.444	0	Gue		[24]
P24	14	38,919,669	39,027,008	0.383	0	Gue		
P25	14	42,121,450	42,376,970	0.389	0			
P26	14	45,478,315	46,437,276	0.430	0			[24]
P27	16	40,318,965	40,656,961	0.390	0			
P28	16	40,886,797	41,149,860	0.383	0			
P29	16	41,564,542	42,407,997	0.440	0	Jer		
P30	16	43,250,880	43,501,100	0.390	0			[27]
P31*	16	44,277,286	45,534,177	0.510	2		PIK3CD	
P32	18	11,298,096	11,959,392	0.409	0	Bro	IRF8	[24]
P33	18	14,171,624	14,702,657	0.423	0		ACSF3, SPATA2L	[10]
P34	20	13,714,109	15,135,107	0.429	0			[24]
P35*	20	71,629,018	71,967,622	0.508	0			
P36*	21	83,766	2,416,432	0.564	0	Gue		
P37*	21	31,681,776	33,273,658	0.530	1	Her		
P38	21	45,793,883	45,979,589	0.391	0			
P39	21	68,349,152	68,943,249	0.448	0	Ang	HSP90AA1, PPP2R5C	
P40	24	24,114,816	24,452,344	0.383	1	Nor		
P41	29	51,452,986	52,452,986	0.382	1			
P42	30	33,064,381	50,329,406	0.810	0		IRAK1, BCAP31, CETN2, GAB3, IKBKG, KIR3DL2, MTM1, SRPK3	
P43	30	53,439,167	57,258,157	0.711	0		BTK	
P44	30	68,834,838	71,300,049	0.861	0		CYLC1	[12]
P45	30	72,360,206	79,580,964	0.844	0			[12]
P46	30	84,352,052	85,706,219	0.764	0		IL2RG	[12]
P47	30	96,229,383	100,603,158	0.699	0		ALAS2, SMC1A, LOC524601, SPIN2, VSIG4	
P48	30	130,500,087	132,116,040	0.670	0		RS1	

^*^Regions containing smoothed FST (sFST) in the top 0.1%.

^**^Regions at sFST top 1% of within taurine and within zebu breeds. Full table of within cattle type results for candidate regions under positive selection is on Additional file 6: Table S3. Ang – Angus; Bro – Brown Swiss, Cha – Charolais, Gue – Guernsey, Jer – Jersey, Her – Hereford, Hol – Holstein, Lim – Limousin, Nor – Norwegian Red, Guz – Guzera, Nel – Nelore.

**Table 2:** **Balancing selection: regions in the bottom 1**% **smoothed F**_**ST**_ **values**

Region	BTA	SNP start pos	SNP end pos	Lowest	CNV [21]	Within cattle type**	Candidate genes	Cross reference
B1	4	110,295,764	111,378,106	0.070	2			
B2	4	111,742,866	112,562,902	0.076	2		CNTNAP2	
B3	5	19,457,756	19,898,237	0.075	0		ATP2B1	
B4	5	76,719,327	77,207,435	0.067	0		PKP2	
B5	6	2,883,313	4,231,143	0.059	6		CCNA2, ANXA5	
B6	6	12,490,545	13,266,473	0.062	1		CAMK2D	
B7	6	54,759,464	55,199,755	0.062	4			
B8	6	61,590,746	61,892,976	0.076	0		APBB2	
B9	6	118,252,961	118,649,364	0.061	0	Guz		
B10	7	65,205,183	65,242,121	0.079	0		GLRA1	
B11	7	98,598,188	99,371,157	0.050	3		ERAP2, LNPEP	
B12	11	11,993,676	13,090,823	0.072	0		DYSF	
B13	11	16,914,701	17,716,204	0.074	2			
B14	12	70,094,561	76,785,743	0.059	6		ABCC4	
B15	14	53,550,213	54,231,380	0.066	2	Nel, Guz, Jer		
B16	16	16,039,261	17,069,240	0.076	1		FAM5C	
B17	16	19,740,336	20,450,779	0.073	0		ESRRG	
B18	16	36,476,830	37,151,556	0.068	1		XCL2	
B19	17	8,512,165	8,575,700	0.079	0			
B20	21	69,852,429	70,269,531	0.054	0	Guz		
B21	22	1,504,583	1,623,884	0.078	1		SEC61G, NEK10	
B22*	23	24,242,547	31,194,961	0.025	30	Ang, Cha, Her, Lim	BOLA (MHC) genes, TNF, AGER, NCR3, C2, CFB, LY6G6F, BTNL2, IL17A, IL17F, CLIC1, CSNK2B, MOG	[12, 24]
B23	23	32,608,468	33,237,258	0.069	1	Cha	ALDH5A1, TDP2, GMNN	
B24	26	46,663,802	47,234,109	0.055	0	Cha		

^*^Regions containing smoothed FST (sFST) in the bottom 0.1%.

^**^Regions at sFST bottom 1% of within taurine and within zebu breeds. Full table of within cattle type results for candidate regions under balancing selection is on Additional file 7: Table S4. Ang – Angus; Bro – Brown Swiss, Cha – Charolais, Gue – Guernsey, Jer – Jersey, Her – Hereford, Hol – Holstein, Lim – Limousin, Nor – Norwegian Red, Guz – Guzera, Nel – Nelore.

#### Regions under positive selection

The top 1% smoothed F_ST_ values were distributed across 48 regions in 17 chromosomes (Table 1) including the BTAX (not shown in Figure 3). Of those, 12 regions were known to harbor copy number variations, and 22 regions had been described as under positive selection in previous studies (Table 1). Twenty of them also overlapped on one or more breed specific peaks in the within cattle type analyses. Among the previously described peaks, 10 of them overlapped to taurine breed signals, and 1 to a zebu breed peak.

The search for overrepresentation of gene ontology terms was not conclusive. Nevertheless, some regions can be highlighted because of their genic content and/or results from previous studies identifying them as being under selection. The BTA7:47.2-53.7 Mb region (Table 1: regions P10 and P11) harbors two closely linked regions that are potentially under selection. These regions contain a number of immune-related and imprinted genes (*CD14*, *HSPA9* and PCDH family) previously identified to be under selection, and associated with cattle fertility (*SPOCK*). Moreover, a number of CNV are located in the same region and linkage disequilibrium (LD) blocks larger than the average genomic LD are present in both taurine and zebu animals with LD blocks varying in length (Additional file 4: Figure S3A). Another interesting region is the BTA14:24.6-25.2 Mb region (Table 1: region P22), which confirmed previous results [10] and was recently associated with cattle production-related traits. Interestingly, the zebu and taurine LD patterns also markedly vary within this region (Additional file 4: Figure S3B). The BTAX is the final region to be highlighted, as almost the entire chromosome was shown to be highly differentiated between taurine and zebu.

#### Regions under balancing selection

The bottom 1% smoothed F_ST_ values consisted of 24 genomic regions across 13 chromosomes (Table 2). Of those, only a region on BTA23 had been previously described as a candidate for balancing selection. This region also overlapped taurine breed signals from the within-taurine analysis. In total, 6 regions overlapped within cattle type analyses, three to zebu breed peaks and four to taurine breeds.

Fourteen of these regions have been described as having CNV. These included the large region (Table 2: B22) on BTA23:24.2-31.1 Mb comprising the BOLA gene family (*MHC – II* molecules) which harbors 30 described CNV. This region has also been previously associated with balancing selection [12, 24] in cattle (Table 2).

The 24 balancing selection regions overlap with 345 Ensembl peptides, corresponding to 318 unique Ensembl genes (Table 2). Additionally ~83% (20/24) of the balancing selection regions completely or partially span cattle Ensembl genes. We assigned PANTHER accessions to a total of 332 overlapping peptides. Statistically significant over represented peptides were observed for multiple categories. Five pathways were found significantly overrepresented (adjusted p-value <0.05): the olfactory transduction, systemic lupus erythematosus, type I diabetes mellitus, antigen processing and presentation, graft-versus-host disease and allograft rejection pathways; all of which could be linked to immune response systems (a biological process also overrepresented).

The average F_ST_ for each chromosome in each analysis can be found in the Additional file 5: Table S2. Also in the supplementary material all top and bottom F_ST_ peaks for all analyses are presented (Additional file 6: Table S3 and Additional file 7: Table S4).

## Discussion

In all, 505 animals derived from 10 taurine and 3 zebu cattle breeds were genotyped across more than 770,000 SNP markers to investigate the genomic changes subsequent to the separation between taurine and zebu cattle, which occurred at a date between 330 thousand and 2 million years ago [1, 2]. Evaluation of the SNP genotyping platform suggested there was minimal bias in properly characterizing both subspecies of animals, except possibly on the sex chromosomes. As expected, most of the chromosomes had a higher proportion of polymorphic markers in taurine animals, also resulting in higher heterozygosity, when compared to zebu (Figure 1A). This is due to the fact that most of the SNP described for cattle were identified using the reference sequence of a taurine animal [19, 28], but this should not overly impact population diversity metrics [29]. Nevertheless, all chromosomes have >80% SNP polymorphic in both cattle types, exception made for BTA1, 13, X and Y (Figure 1B), providing a large number of informative markers.

Clustering animals based on the genetic relationship matrix, not surprisingly, split the animals into two groups (taurine and zebu) in agreement to the division along the first principal component and the magnitude of pair-wise F_ST_ between breeds. The split along the second principal component between taurine breeds suggests that there is more variation within this cattle type than there is within zebu. Since it is known that unbalanced principal components analyses could mislead interpretations of population structures [30], four randomized evenly sampled analyses were run (Additional file 2: Figure S2). These additional analyses supported the previous results. This could be partly due to more intensive selection and reproductive isolation in taurine breeds than among zebu cattle. However, even though the BovineHD BeadChip was developed to minimize potential ascertainment bias, one cannot entirely reject the possibility that the subdivision seen on principal component 2 was due to this potential bias carried over by the genotyping platform. In the near future when whole genome sequences from a number of breeds and cattle types become available a definitive conclusion about this aspect will be drawn.

The BTAX and Y carry a great number of SNP with high difference in allelic frequencies between groups. These chromosomes have probably undergone much stronger selection or, more parsimoniously, higher genetic drift, due to their unique inheritance [6], and the history of domestication, selection, breed formation. Furthermore, the intensive use of artificial insemination techniques have likely contributed to the reduction of genetic variability within breeds (or cattle types) in these chromosomes. It is understood that in the case of the SNP that are polymorphic in both cattle types, the alternative allele likely arose within the cattle population before the split between taurine and zebu, and remained in both populations at variable frequencies. Alleles that are fixed in one subspecies and variable in the other possibly arose after the split. However, this understanding does not take into account that alleles that were fixed in one population also might have arisen before the split, but were fixed due to different selection processes or as a result of different bottlenecks on the populations. The identification of the ancestral allele of these SNP, ideally using whole genome sequences of other Bovids, would contribute to understand the evolutionary processes behind these monomorphic sites.

The use of metrics based on variance of allelic frequencies in order to identify genomic regions that are potentially under selection, such as F_ST_, have already been explored in a number of studies using cattle [10, 28, 31], sheep [9] and dogs [8]. In this study a relatively high density of markers (average gap between markers 4Kb) was applied to detect genomic differences between zebu and taurine using F_ST_, identifying regions that were potentially associated with different types of selection. Due to their original geographic distribution, taurine cattle are more adapted to temperate climate, while zebu cattle are better adapted than most taurine cattle to tropical environments. Therefore, differences between these two cattle could be linked to adaptation to the environment; however, it is likely that selection imposed by humans in different geographical locations and livestock-product production goals may have also produced regions that were under differing selective pressures. This study, the most comprehensive to date for cattle, identified 48 regions under potential positive and 24 under balancing selection.

A number of these positive selection candidates have been identified to be under selection in previous studies (22 out of 48, Table 1). These previous studies cannot strictly be considered independent analyses since a subset of markers included in the analyses presented here were already used in those. However, in this work more than a 10 fold increase in marker density was used, thus reducing the overlap of SNP across experiments to less than 10%. Further, different cattle samples and populations were used. Thereafter, even though not absolutely independent, from previous studies, our results lend support to the findings from previous articles provide new insights on ancient differentiation between zebu and taurine cattle. These regions may be genomic segments that were under natural selection or drift, but in fact, might for instance represent zebu fragments that were introgressed in taurine breed potentially defining low-level admixed populations [24, 25]. A parallel could also be drawn to described QTLs that overlap these highly divergent genomic regions, e.g. on BTA14:~25 Mb which harbors quantitative loci for stature [32], fertility [33–35] and subcutaneous fat [36]. The different LD structure in these regions supports the concept of introgressed segments as a way of sharing recent polymorphisms between the cattle types [37], and defines quantitative loci and signatures of selection.

The highest differentiation peak was found in BTA7:~50 Mb. This region had previously been identified as a site containing a signature of selection [12, 24]. A number of features were also identified in this region, including different LD structure between zebu and taurine cattle, the presence of imprinted genes, and potential association to fertility traits. This region is among the very few regions for positive selection that also contain CNVs; which may seem antagonistic to purifying selection. It is not clear at this point how CNV are being kept in the population at this site and at the same time there is a differential signal for zebu and taurine cattle. It could be in consequence that these CNV being less likely in LD with neighbouring SNPs, because similar CNVs can occur on different haplotype backgrounds. Another possibility is that duplications can initiate gene conversion events, which can then decrease the LD surrounding such variants. Interestingly, CNVs were often observed at most candidate sites for balancing selection, where variation is expected.

Fourteen out of 24 balancing selection regions overlap identified CNVs, including the highly variable region on BTA23: ~24 Mb with 30 described CNV (Table 2). This set of balance selection-derived genes possess a wide spectrum of molecular functions and provide a rich resource for testing hypotheses on the genetic basis of phenotypic variation within and among breeds. Consistent with similar analyses in other mammals (human, mouse and dog), several of these genes, which are important in drug detoxification, defense/innate and adaptive immunity, are also highlighted by these analyses in cattle. These gene families include the bovine MHC (BoLA), ATP-binding cassette (ABC) transporters, Glutathione S-transferases, Complement factors, Interleukin-17A (*IL17A*), Heat shock 70 kDa protein 1A (*HSPA1A*), Chloride intracellular channel protein 1 (*CLIC1*), and Casein kinase II subunit beta (*CSNK2B*), which support the shared GO terms among mammals. Conservation of these genes across mammals suggests that selective pressure may drive acquisition or retention of species-specific gene functions.

On the other hand, lineage-specific selection events were detected in mammals, especially in mice and rats. In this regard, it is intriguing to note that mammary gland development genes, such as Butyrophilin-like protein 2 (*BTNL2*) and Myelin-oligodendrocyte glycoprotein (*MOG*) were enriched in GO Biological process on the PANTHER analyses. We also detected marked variation between individuals and across diverse cattle breeds, which indicates that these selection events may have occurred within the artiodactyla and/or *Bos* lineages contributing to cattle speciation and domestication.

Genome-wide, most CNVs evolved under neutral evolutionary pressures. Their frequency and sequence context were shaped by demographic events, mutation rate and genetic drift. However, most CNVs in potentially functional regions, especially those overlapping genes, are under purifying selection and there are only a few examples of CNVs on these positive selection sites. Regions that differ in copy number between subspecies can be informative about ancient adaptations that may have led to species-specific phenotypes [38]. Recent copy number changes can inform about human selection that may have led to genetic and phenotypic differences between breeds.

Similar to selection for variability seen in balancing regions that result in low F_ST_ values, it is worth noting that low values could also represent purifying selection forces that are simultaneously applied in both populations in the same direction, imposing high similarity between the compared groups which would result in low differentiation (low F_ST_). In this case, a potential deleterious mutation affecting both populations would be selected against in both groups. This can partially explain the high frequency of genes associated to Mendelian diseases within those potential balancing selection regions. Highlighting a few examples, Dysferlin (*DYSF*) is associated to muscular dystrophy [39], ATPase, Ca (2+)-transporting, plasma membrane, 1 (*ATP2B1*), where mouse knockouts have identified variation underlying embryonic lethality, and has a critical role in male fertility [40], Plakophilin 2 (*PKP2*), which is linked to circulatory system conditions [41, 42], and Cyclin A (*CCNA2*) that is an essential regulatory molecule for the cell’s cycle [43]. It is not clear at this point, and it will require further investigation to define if the selection signals seen in these regions are due to the presence of those candidate genes or not.

It is not completely clear at this point how the observed signals of selection originated. The within-taurine and within-zebu F_ST_ complement the taurine-zebu contrast analysis providing hints on the breed driving each signal. From the autossomal regions previously described as candidate regions under positive selection, around half of them overlap to signals of one or more breeds in the within-taurine analysis (10 out of 19), which is consistent with one’s expectation, since the majority of previous work was done using mostly taurine breeds, and in a few cases also composite cattle. There was only one region previously described as a candidate for balancing selection, in BTA23, and this also overlaps with within-breed type signals. A number of peaks were characterized with more than one breed specific peak in the within-breed analyses, supporting a commonality of selective pressure in at least a few regions in some breeds. However, not all observed signals from the comparison taurine-zebu could be attributed to a specific breed (s), and these suggest that they represent a deeper degree of separation and, possibly, adaptation between cattle types.

In summary, genomic regions that are linked to positive and balancing selection were detected within taurine and zebu cattle, which represent the major sub-division of domestic cattle. A number of previously described regions containing positive selection were confirmed. Novel selection regions were likely discovered due to the higher resolution of informative SNPs available in this study compared to previous analyses. Some of these regions overlap with production QTL, and e.g. immune-related genes, suggesting that favorable variations to adaptation and production are present in the general cattle population, however the application of these results into breeding programs to accelerate creation of synthetic breeds with high production value in tropical environments remains elusive until subsequent investigations confirm the underlying effect of the variants underlying the signatures. This information is needed to define breeding systems able to efficiently introgress specific genomic fragments of zebu in taurine cattle and vice-versa.

## Conclusions

Genomic regions that are potentially linked to purifying or balancing selection processes in domestic cattle were identified genome-wide. The genetic variants imposing such selective pressure are not known, even though for some regions candidate genes could be assigned, and could serve as resource for new hypothesis testing in the future. These regions are of particular interest to understand the natural and human selective pressures to which these subspecies were exposed and how the genetic background of these populations evolved in response to environmental challenges and human manipulation.

### Availability of supporting data

Supporting information is available in the additional files and further supporting data is available from the authors on request.

## Electronic supplementary material

Additional file 1: Figure S1: Population substructure, the main division in domestic cattle (based on 505 individuals, 38,681 SNP). A) Unsupervised clustering result (inferred number of clusters K = 2). The two clusters represent the main division in ancestry of domestic cattle, the zebu (red) and taurine (blue). The estimated proportion of each cluster (y) is given for each individual. #1-91 Nelore, #92-141 Gir, #142-166 Guzera, #167-187 Guernsey, #188-226 Jersey, #227-270 – Angus, #271-281 Red Angus, #282-317 Hereford, #318-364 Limousin, #365-401 Charolais, #402-425 Brown Swiss, #426-488 Holstein, #489-505 Norwegian Red. B-C) Principal components analysis (PCA1 vs PCA2), taurine and zebu animals are plotted B) by cattle type zebu (blue) and taurine (red), and C) by breed. (TIFF 911 KB)

Additional file 2: Figure S2: “Balanced” principal components analyses (PCA). In order to investigate if the distribution of the breeds within the principal components factorial plan was due to the uneven number of individuals in each breed, four independent evenly balanced PCA were run. (TIFF 354 KB)

Additional file 3: Table S1: Wright’s F-statistics FIS and pair-wise FST between cattle breeds based on 768,506 SNP genotypes. (PDF 72 KB)

Additional file 4: Figure S3: Linkage Disequilibrium (r^2^) of selected regions potentially under positive selection. a) BTA7:47 – 54 Mb. b) BTA14: 24 – 26 Mb. (TIFF 1 MB)

Additional file 5: Table S2: Average F_ST_ per chromosome for each analysis. (PDF 61 KB)

Additional file 6: Table S3: Candidate region for positive selection: top 1% smoothed F_ST_ values for all breeds in all analyses. (PDF 2 MB)

Additional file 7: Table S4: Candidate regions for balancing selection: bottom 1% smoothed F_ST_ values for all breeds in all analyses. (PDF 769 KB)
