# Supplementary figures and images for: Genomic divergence of zebu and taurine cattle identified through high-density SNP genotyping
Source: BMC Genomics. 2013 Dec 13;14(1):876. doi: 10.1186/1471-2164-14-876 (PMC4046821; doi:10.1186/1471-2164-14-876)

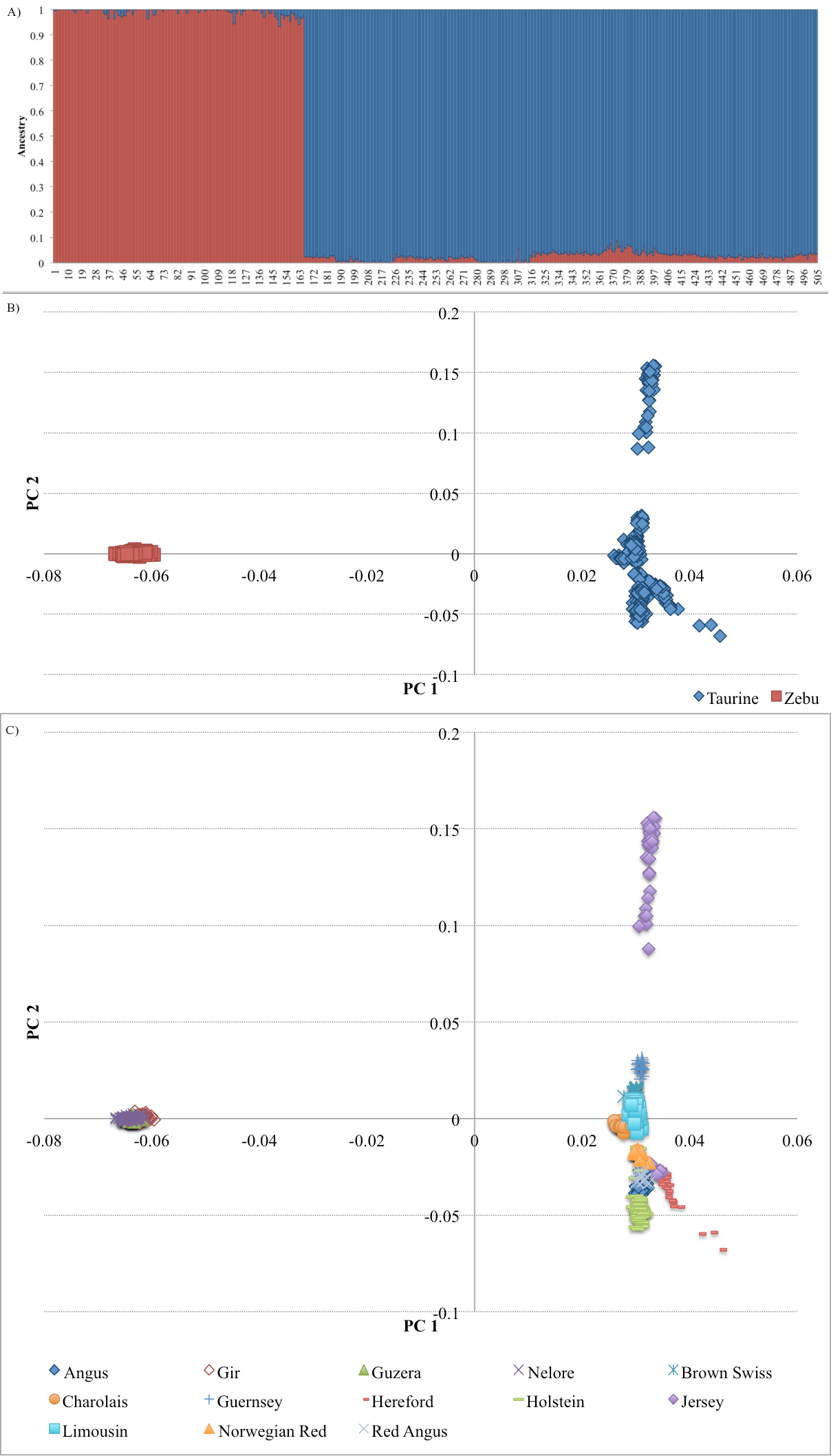

Supplement: Supplementary file 1 — Additional file 1: Figure S1: Population substructure, the main division in domestic cattle (based on 505 individuals, 38,681 SNP). A) Unsupervised clustering result (inferred number of clusters K = 2). The two clusters represent the main division in ancestry of domestic cattle, the zebu (red) and taurine (blue). The estimated proportion of each cluster (y) is given for each individual. #1-91 Nelore, #92-141 Gir, #142-166 Guzera, #167-187 Guernsey, #188-226 Jersey, #227-270 – Angus, #271-281 Red Angus, #282-317 Hereford, #318-364 Limousin, #365-401 Charolais, #402-425 Brown Swiss, #426-488 Holstein, #489-505 Norwegian Red. B-C) Principal components analysis (PCA1 vs PCA2), taurine and zebu animals are plotted B) by cattle type zebu (blue) and taurine (red), and C) by breed. (TIFF 911 KB) [file 12864_2012_5571_MOESM1_ESM.tiff]

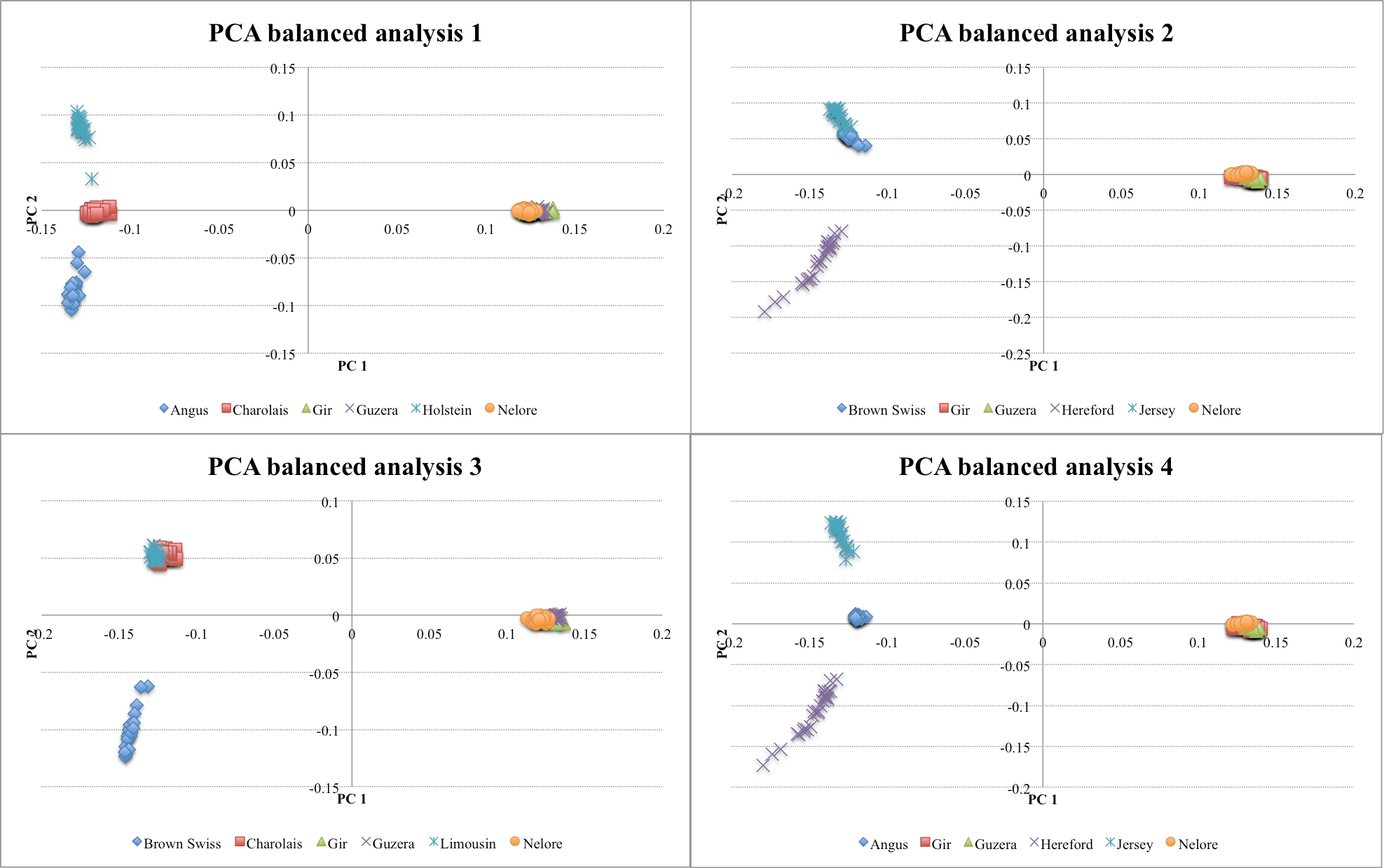

Supplement: Supplementary file 2 — Additional file 2: Figure S2: “Balanced” principal components analyses (PCA). In order to investigate if the distribution of the breeds within the principal components factorial plan was due to the uneven number of individuals in each breed, four independent evenly balanced PCA were run. (TIFF 354 KB) [file 12864_2012_5571_MOESM2_ESM.tiff]

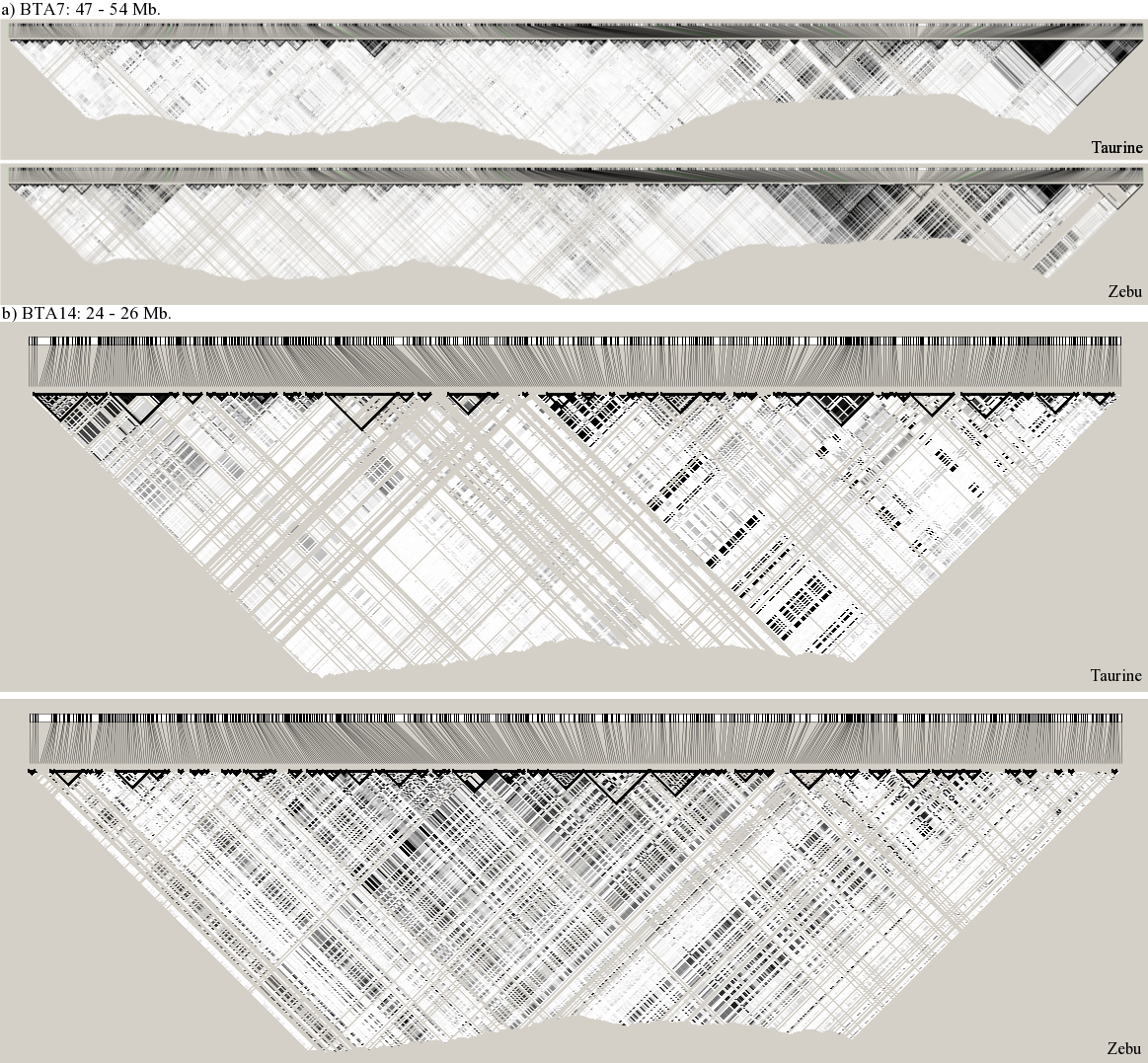

Supplement: Supplementary file 4 — Additional file 4: Figure S3: Linkage Disequilibrium (r2) of selected regions potentially under positive selection. a) BTA7:47 – 54 Mb. b) BTA14: 24 – 26 Mb. (TIFF 1 MB) [file 12864_2012_5571_MOESM4_ESM.tiff]
