# Supplementary material for: Genomic divergence of zebu and taurine cattle identified through high-density SNP genotyping
Source: BMC Genomics. 2013 Dec 13;14(1):876. doi: 10.1186/1471-2164-14-876 (PMC4046821; doi:10.1186/1471-2164-14-876)
Supplement: Supplementary file 3 — Additional file 3: Table S1: Wright’s F-statistics FIS and pair-wise FST between cattle breeds based on 768,506 SNP genotypes. (PDF 72 KB) [file 12864_2012_5571_MOESM3_ESM.pdf]

Supplementary table 1. Wright's F-statistics FIS and pair-wise FST between cattle breeds based on 768,506 SNP genotypes.

	FIS	Pair-wise FST												
		**Angus**	**Brown Swiss**	**Charolais**	**Gir**	**Guernsey**	**Guzera**	**Hereford**	**Holstein**	**Jersey**	**Limousin**	**Nelore**	**Norwegian Red**	**Red Angus**
**Angus**	0.0129	0	0.1585	0.0847	0.4331	0.1467	0.4109	0.1522	0.1127	0.1750	0.0983	0.4436	0.0975	0.0237
**Brown Swiss**	-0.0410	0.1585	0	0.1066	0.4627	0.1704	0.4430	0.1833	0.1477	0.1971	0.1108	0.4687	0.1438	0.1648
**Charolais**	0.0125	0.0847	0.1066	0	0.4025	0.1021	0.3759	0.1151	0.0825	0.1331	0.0425	0.4162	0.0668	0.0814
**Gir**	0.0054	0.4331	0.4627	0.4025	0	0.4638	0.0537	0.4715	0.4180	0.4725	0.4113	0.0672	0.4458	0.4628
**Guernsey**	0.0212	0.1467	0.1704	0.1021	0.4638	0	0.4430	0.1675	0.1341	0.1691	0.1096	0.4703	0.1295	0.1507
**Guzera**	0.0020	0.4109	0.4430	0.3759	0.0537	0.4430	0	0.4444	0.3968	0.4547	0.3873	0.0654	0.4211	0.4407
**Hereford**	0.0761	0.1522	0.1833	0.1151	0.4715	0.1675	0.4444	0	0.1537	0.1997	0.1319	0.4869	0.1320	0.1435
**Holstein**	-0.0081	0.1127	0.1477	0.0825	0.4180	0.1341	0.3968	0.1537	0	0.1615	0.0953	0.4298	0.0864	0.1149
**Jersey**	-0.0062	0.1750	0.1971	0.1331	0.4725	0.1691	0.4547	0.1997	0.1615	0	0.1392	0.4789	0.1617	0.1825
**Limousin**	0.0040	0.0983	0.1108	0.0425	0.4113	0.1096	0.3873	0.1319	0.0953	0.1392	0	0.4242	0.0849	0.0986
**Nelore**	-0.0018	0.4436	0.4687	0.4162	0.0672	0.4703	0.0654	0.4869	0.4298	0.4789	0.4242	0	0.4539	0.4673
**Norwegian Red**	-0.0075	0.0975	0.1438	0.0668	0.4458	0.1295	0.4211	0.1320	0.0864	0.1617	0.0849	0.4539	0	0.0981
**Red Angus**	-0.0035	0.0237	0.1648	0.0814	0.4628	0.1507	0.4407	0.1435	0.1149	0.1825	0.0986	0.4673	0.0981	0
