# Supplementary material for: Genomic divergence of zebu and taurine cattle identified through high-density SNP genotyping
Source: BMC Genomics. 2013 Dec 13;14(1):876. doi: 10.1186/1471-2164-14-876 (PMC4046821; doi:10.1186/1471-2164-14-876)
Supplement: Supplementary file 5 — Additional file 5: Table S2: Average FST per chromosome for each analysis. (PDF 61 KB) [file 12864_2012_5571_MOESM5_ESM.pdf]

Supplementary table 2. Average FST per chromosome for each analyses.

BTA	FST zebu vs taurine		FST within zebu breeds				FST within taurine breeds									
	N_SNP	FST	N_SNP	Nelore	Gir	Guzera	N_SNP	Angus	Brown Swiss	Charolais	Guernsey	Hereford	Holstein	Jersey	Limousin	Norwegian Red
**1**	45750	0.203	40875	0.044	0.018	0.027	43540	0.108	0.135	0.055	0.134	0.141	0.104	0.136	0.067	0.086
**2**	39429	0.216	34453	0.050	0.019	0.029	38043	0.096	0.123	0.064	0.119	0.176	0.113	0.117	0.081	0.077
**3**	35002	0.204	30599	0.042	0.018	0.025	33583	0.142	0.133	0.061	0.133	0.123	0.104	0.137	0.059	0.087
**4**	34384	0.206	30490	0.042	0.017	0.024	32506	0.129	0.151	0.058	0.145	0.129	0.098	0.119	0.068	0.116
**5**	34163	0.209	29735	0.055	0.022	0.033	32421	0.141	0.206	0.089	0.124	0.151	0.104	0.104	0.061	0.110
**6**	35015	0.164	31605	0.053	0.025	0.032	33931	0.121	0.242	0.071	0.140	0.154	0.106	0.139	0.069	0.097
**7**	32568	0.211	28996	0.044	0.019	0.027	31726	0.116	0.141	0.067	0.126	0.155	0.118	0.131	0.078	0.085
**8**	33011	0.259	29782	0.049	0.019	0.028	29925	0.105	0.134	0.072	0.131	0.095	0.102	0.106	0.056	0.085
**9**	30569	0.184	27630	0.049	0.019	0.031	29321	0.102	0.140	0.058	0.130	0.183	0.093	0.105	0.063	0.095
**10**	29956	0.202	25931	0.040	0.019	0.025	28869	0.089	0.117	0.053	0.137	0.138	0.112	0.104	0.067	0.108
**11**	31508	0.190	27488	0.047	0.019	0.027	30904	0.125	0.192	0.066	0.196	0.156	0.099	0.108	0.076	0.105
**12**	25505	0.207	21882	0.043	0.020	0.026	24059	0.114	0.141	0.065	0.130	0.122	0.097	0.104	0.062	0.086
**13**	23171	0.247	19960	0.054	0.021	0.034	20724	0.151	0.165	0.070	0.148	0.141	0.127	0.120	0.070	0.106
**14**	24358	0.231	21935	0.051	0.020	0.032	22185	0.102	0.140	0.075	0.113	0.132	0.107	0.105	0.082	0.122
**15**	24227	0.198	21560	0.052	0.021	0.027	23626	0.122	0.143	0.060	0.146	0.117	0.082	0.131	0.070	0.093
**16**	23759	0.195	21828	0.054	0.019	0.033	23361	0.111	0.186	0.060	0.124	0.115	0.114	0.102	0.064	0.074
**17**	21906	0.181	19248	0.047	0.020	0.027	21398	0.112	0.143	0.056	0.140	0.185	0.095	0.106	0.063	0.093
**18**	19001	0.178	16826	0.047	0.019	0.027	18560	0.106	0.169	0.069	0.132	0.143	0.086	0.125	0.061	0.115
**19**	18572	0.191	15918	0.048	0.020	0.031	18393	0.126	0.172	0.065	0.174	0.145	0.106	0.140	0.078	0.098
**20**	21171	0.182	18415	0.050	0.019	0.029	20813	0.100	0.148	0.057	0.138	0.123	0.129	0.142	0.065	0.080
**21**	20770	0.209	18087	0.046	0.020	0.027	20207	0.101	0.131	0.068	0.128	0.119	0.097	0.114	0.082	0.110
**22**	17747	0.174	15520	0.052	0.021	0.030	17229	0.119	0.167	0.050	0.159	0.127	0.109	0.115	0.069	0.100
**23**	14913	0.160	13482	0.042	0.018	0.027	14594	0.083	0.116	0.050	0.106	0.132	0.090	0.107	0.068	0.117
**24**	18314	0.213	16180	0.039	0.017	0.023	17692	0.108	0.164	0.057	0.136	0.170	0.097	0.120	0.061	0.092

**25**	12679	0.174	11098	0.036	0.019	0.023	12508	0.099	0.158	0.054	0.117	0.132	0.102	0.100	0.062	0.093
**26**	14959	0.165	13480	0.044	0.018	0.026	14461	0.108	0.140	0.053	0.131	0.130	0.113	0.125	0.080	0.101
**27**	12924	0.171	11215	0.041	0.018	0.026	12493	0.116	0.138	0.054	0.139	0.124	0.086	0.124	0.065	0.088
**28**	12771	0.170	11568	0.050	0.021	0.027	12630	0.083	0.128	0.057	0.128	0.107	0.089	0.110	0.073	0.098
**29**	14417	0.183	12716	0.042	0.017	0.024	14228	0.077	0.170	0.074	0.144	0.116	0.097	0.125	0.078	0.086
**X**	38099	0.475	35950	0.029	0.021	0.034	22704	0.086	0.106	0.102	0.102	0.078	0.087	0.082	0.065	0.087
**Y**	109	0.681	-	-	-	-	34	0.534	0.370	0.848	0.400	0.221	0.558	0.433	0.402	0.560
