# Supplementary material for: Genomic divergence of zebu and taurine cattle identified through high-density SNP genotyping
Source: BMC Genomics. 2013 Dec 13;14(1):876. doi: 10.1186/1471-2164-14-876 (PMC4046821; doi:10.1186/1471-2164-14-876)
Supplement: Supplementary file 6 — Additional file 6: Table S3: Candidate region for positive selection: top 1% smoothed FST values for all breeds in all analyses. (PDF 2 MB) [file 12864_2012_5571_MOESM6_ESM.pdf]

Supplementary table 3. Summary of candidate regions in autosomes of positive selection detected using smoothed FST in three analyses: 1) comparing zebu-taurine animals, 2) within taurine (n = 9 breeds), and 3) within zebu (n = 3 breeds).

The top 1% smoothed FST values for each breed in each analysis were considered peaks. For each analyses separately, if two breed specific peaks were less than 50Kb from each other they were joined into a single peak-region. In this table no attempt was made to join any breed specific peak-regions or to identify peak-regions in common (or overlaps) across breeds and across the different analyses.

BTA	Peak_start	Peak_end	Zebu-Taurine	Within_taurine	Within_zebu
1	399506	399506		Nor	
1	571340	571340		Her	
1	1094456	1094456		Jer	
1	1666781	1666781		Bro	
1	3129850	3129850		Her	
1	3246679	3246679		Nor	
1	3807798	3807798		Jer	
1	4010489	4010489		Gue	
1	4282516	4282516		Bro	
1	7665323	7665323		Gue	
1	8111905	8111905		Ang	
1	9684361	9684361		Jer	
1	10346929	10346929		Gue	
1	11161020	11161020		Nor	
1	11482232	11482232		Jer	
1	12380238	12380238		Gue	
1	12380238	12380238		Nor	
1	12488078	12488078		Lim	
1	13697596	13697596		Lim	
1	14624014	14624014		Ang	
1	15554023	15554023		Ang	
1	15554023	15554023		Hol	
1	15976386	15976386		Jer	
1	16627180	16627180		Her	
1	19152223	19152223		Jer	
1	19799108	19799108		Ang	
1	20071616	20071616		Nor	
1	20402008	20402008		Lim	
1	20786987	20786987		Jer	
1	21278811	21278811		Bro	
1	22510052	22510052		Ang	
1	23196692	23196692		Ang	
1	24059226	24059226		Ang	
1	24164664	24164664		Nor	
1	25999738	25999738		Nor	
1	27913863	27913863		Her	
1	28820688	28820688		Hol	
1	33631349	33631349		Hol	

1	33663674	33663674	Gue	
1	34331115	34331115	Gue	
1	35056086	35056086	Lim	
1	36323950	36323950	Jer	
1	37100951	37100951	Lim	
1	37603937	37603937	Ang	
1	38307976	38307976		Nel
1	38875334	38875334	Her	
1	38908284	38908284	Ang	
1	39045328	39045328	Lim	
1	39606695	39606695	Bro	
1	39844099	39844099	Nor	
1	41814694	41814694	Jer	
1	47743810	47743810	Bro	
1	48338528	48338528	Bro	
1	48772543	48772543	Jer	
1	48833934	48833934	Gue	
1	49861333	49861333	Her	
1	50067523	50097027	Nor	
1	50337394	50337394	Nor	
1	54625163	54625163	Bro	
1	55370763	55370763	Bro	
1	55499124	55499124	Nor	
1	55735039	55735039	Bro	
1	55881470	55881470	Hol	
1	55998138	55998138	Her	
1	56027212	56027212	Gue	
1	56143825	56143825	Ang	
1	56598694	56598694	Gue	
1	56620756	56620756	Lim	
1	56874618	56874618	Gue	
1	56949298	56949298	Nor	
1	59300477	59300477	Lim	
1	61365597	61365597	Hol	
1	61931836	61931836	Gue	
1	63366408	63366408	Ang	
1	63644513	63644513	Bro	
1	63674787	63983394		Nel
1	66877916	66877916	Bro	
1	67291110	67291110	Ang	
1	67291110	67291110	Jer	
1	68098581	68098581	Lim	
1	69295961	69295961	Gue	
1	70759781	70759781	Hol	
1	72286379	72286379	Bro	
1	72649349	72649349	Gue	
1	72724406	72724406	Bro	
1	74302401	74302401	Bro	

1	74585891	74585891	Gue
1	74679286	74718807	Bro
1	74679286	74718807	Cha
1	74822552	74822552	Bro
1	74822552	74822552	Cha
1	75028047	75028047	Cha
1	75135995	75309533	Cha
1	75206924	75206924	Jer
1	75367346	75367346	Hol
1	75553499	75553499	Lim
1	75574770	75574770	Ang
1	75736512	75736512	Jer
1	75811289	75811289	Nor
1	75993310	75993310	Lim
1	76110765	76110765	Lim
1	76285407	76527417	Cha
1	79155749	79155749	Hol
1	79810878	79810878	Bro
1	79976244	79976244	Lim
1	80441713	80441713	Hol
1	81289271	81289271	Ang
1	83309970	83309970	Lim
1	83903856	83903856	Bro
1	85888115	85888115	Her
1	87258985	87258985	Hol
1	87381119	87381119	Jer
1	88462591	88462591	Jer
1	88801264	88801264	Gue
1	89245146	89245146	Nor
1	89600494	89600494	Gue
1	89697566	89697566	Bro
1	89828304	89828304	Bro
1	90704654	90704654	Jer
1	90919291	90919291	Ang
1	91066621	91066621	Lim
1	91823434	91823434	Nor
1	91890443	91890443	Ang
1	92014411	92014411	Lim
1	92186479	92186479	Lim
1	92417044	92417044	Hol
1	93027728	93027728	Her
1	93608310	93672088	Cha
1	93608310	93608310	Hol
1	93841104	94409201	Cha
1	93841104	93841104	Hol
1	94199961	94199961	Lim
1	94361121	94361121	Jer
1	94511587	94511587	Hol

1	96477076	96477076	Jer	
1	96923981	96923981	Her	
1	97254722	97254722	Jer	
1	99261398	99261398	Bro	
1	100108192	100108192	Lim	
1	102140097	102140097	Nor	
1	102349222	102349222	Nor	
1	102403092	102403092	Bro	
1	102628793	102628793	Gue	
1	102888992	102888992	Nor	
1	103117773	103117773	Ang	
1	103370251	103370251	Bro	
1	105018867	105018867	Gue	
1	106400046	106400046	Gue	
1	106665821	106665821	Nor	
1	106833049	106833049	Jer	
1	106881204	106881204	Nor	
1	107241380	107241380	Gue	
1	107410516	107410516	Bro	
1	107410516	107410516	Her	
1	108099284	108099284	Nor	
1	108708542	108708542	Her	
1	111674525	111674525	Nor	
1	112734752	112734752	Gue	
1	113499809	113499809	Jer	
1	115074035	115074035	Nor	
1	115385404	115385404	Nor	
1	115752909	115752909	Her	
1	115752909	115752909	Hol	
1	115954859	115954859	Bro	
1	117567419	117567419	Ang	
1	117588730	117829536		Guz
1	117699067	117699067	Bro	
1	117926170	117926170	Lim	
1	121191922	121191922	Lim	
1	121945003	121945003	Hol	
1	122427093	122427093	Her	
1	122787987	122787987	Bro	
1	122787987	122787987	Hol	
1	124396724	124396724	Nor	
1	124596574	124596574	Hol	
1	125064596	125064596	Nor	
1	126538395	126538395	Ang	
1	126994435	126994435	Gue	
1	128255883	128255883	Her	
1	129741579	129741579	Lim	
1	130552004	130552004	Ang	
1	130552004	130552004	Gue	

1	130820087	130820087	Jer	
1	131011331	131011331	Gue	
1	134798943	134798943	Jer	
1	135031329	135031329	Jer	
1	135594701	135594701	Hol	
1	136137365	136137365	Bro	
1	136567014	136567014	Ang	
1	139159980	139251277		Gir
1	139560861	139560861	Lim	
1	139847652	139847652	Gue	
1	140273555	140273555	Her	
1	140709356	140709356	Lim	
1	141685250	141728055	Her	
1	142199519	142199519	Hol	
1	142425137	142425137	Her	
1	142551927	142551927	Jer	
1	142766186	142766186	Jer	
1	142856767	142887803	Ang	
1	142856767	142856767	Nor	
1	143016693	143164410		Guz
1	143016693	143141556		Nel
1	143145310	143145310	Her	
1	144192798	144192798	Bro	
1	144204684	144414936		Guz
1	144429157	144429157	Nor	
1	144524860	144524860	Lim	
1	145547429	145547429	Ang	
1	145842336	145842336	Nor	
1	147812230	147920597		Nel
1	148203263	148277313		Gir
1	148284136	148284136	Her	
1	148405539	148405539	Ang	
1	148514653	148514653	Hol	
1	150055733	150055733	Jer	
1	150367867	150367867	Jer	
1	151315136	151315136	Her	
1	151355656	151355656	Hol	
1	151508673	151508673	Lim	
1	151687326	151687326	Bro	
1	152630666	152630666	Ang	
1	153076432	153076432	Nor	
1	153528274	153528274	Bro	
1	153806681	153806681	Ang	
1	154016701	154016701	Bro	
1	155167189	155167189	Her	
1	155674973	155674973	Gue	
1	156700396	156700396	Jer	
1	157849523	157849523	Nor	

1	157883533	157883533	Hol
1	158282513	158282513	Hol
2	646808	646808	Ang
2	1596441	1596441	Gue
2	2280719	2280719	Bro
2	4136804	4136804	Nor
2	4157616	4157616	Hol
2	4389239	4389239	Lim
2	4568324	4568324	Lim
2	5041395	5041395	Bro
2	5870153	5870153	Bro
2	5977564	5977564	Jer
2	6022748	6022748	Bro
2	9102872	9102872	Hol
2	9418115	9418115	Jer
2	10174040	10174040	Ang
2	10348519	10348519	Lim
2	11640024	11640024	Her
2	11884273	11884273	Jer
2	12436034	12436034	Bro
2	12480914	12480914	Ang
2	12533969	12533969	Lim
2	13301199	13301199	Lim
2	13396487	13396487	Her
2	13682527	13682527	Lim
2	13723329	13723329	Bro
2	14211270	14211270	Nor
2	16429115	16429115	Gue
2	17111598	17111598	Lim
2	17921005	17921005	Ang
2	18102582	18102582	Gue
2	18180730	18180730	Jer
2	18357045	18357045	Bro
2	18685033	18685033	Lim
2	19280130	19280130	Gue
2	23693202	23693202	Ang
2	24382976	24382976	Nor
2	24423751	24423751	Jer
2	24737161	24737161	Nor
2	26011778	26011778	Bro
2	27066560	27066560	Hol
2	27136048	27136048	Nor
2	27859036	27859036	Hol
2	27859036	27859036	Nor
2	28391327	28391327	Ang
2	28672357	28672357	Bro
2	28672357	28672357	Gue
2	28870029	28870029	Jer

2	30323278	30323278		Nor
2	31966138	31966138		Her
2	32329120	32329120		Jer
2	32439651	32439651		Ang
2	33636445	33636445		Jer
2	34919470	34919470		Lim
2	35402595	35402595		Bro
2	36270652	36270652		Nor
2	36763328	36763328		Her
2	37304367	37304367		Lim
2	37526672	37526672		Jer
2	37702062	37702062		Lim
2	39089169	39089169		Bro
2	39089169	39089169		Hol
2	40094725	40094725		Ang
2	40094725	40094725		Hol
2	40850499	40850499		Gue
2	41908338	41908338		Her
2	44204687	44204687		Jer
2	46655612	46655612		Lim
2	47857335	48065161	ZT	
2	48516124	48516124		Nor
2	52168719	52168719		Nor
2	52946980	52946980		Hol
2	53180385	53180385		Jer
2	53552010	53552010		Bro
2	53775362	53775362		Her
2	53995940	53995940		Jer
2	55414166	55414166		Bro
2	56693788	56693788		Ang
2	57800824	57800824		Gue
2	58824184	58824184		Lim
2	59591867	59591867		Ang
2	59875940	59875940		Her
2	60063026	60063026		Ang
2	60115259	60115259		Lim
2	60566784	60566784		Nor
2	61244832	61244832		Ang
2	61244832	61244832		Gue
2	61244832	61244832		Hol
2	61506516	61506516		Gue
2	61569580	61569580		Hol
2	61613191	62280025		Cha
2	61766123	61766123		Gue
2	62380547	62380547		Bro
2	62380547	62440950		Cha
2	62946327	62946327		Lim
2	62979265	62979265		Gue

2	62979265	62979265		Her	
2	63966741	63966741		Jer	
2	64385878	64385878		Bro	
2	65695312	65695312		Hol	
2	65804538	65804538		Her	
2	66233586	66649528			Guz
2	66391542	66558980			Nel
2	67515924	67515924		Hol	
2	69251179	69251179		Hol	
2	69527656	69527656		Her	
2	70717882	70717882		Lim	
2	71565086	72228200	ZT		
2	71893504	71893504		Hol	
2	72329320	72885823	ZT		
2	73274348	73274348		Gue	
2	74090172	74173898		Cha	
2	74202772	74202772		Lim	
2	74642174	74642174		Hol	
2	74642174	74642174		Jer	
2	75210246	75210246		Nor	
2	75885171	76083544		Cha	
2	76766037	76766037		Gue	
2	78609064	78609064		Bro	
2	78893301	78893301		Jer	
2	79153842	79153842		Bro	
2	79259841	79525424			Guz
2	79490369	79525424			Nel
2	79642876	80179443			Guz
2	79642876	80066104			Nel
2	80521364	80521364		Lim	
2	81527119	81527119		Ang	
2	82245159	82245159		Bro	
2	82448538	82448538		Bro	
2	82763789	82763789		Jer	
2	83349093	83349093		Jer	
2	83500338	83500338		Ang	
2	84064764	84064764		Hol	
2	84348194	84348194		Lim	
2	84954520	84954520		Gue	
2	87553236	87553236		Lim	
2	88047008	88047008		Hol	
2	89756351	89756351		Her	
2	90734138	90734138		Her	
2	91614274	91614274		Bro	
2	92080445	92080445		Jer	
2	92396877	92396877		Lim	
2	93325192	93325192		Ang	
2	93408277	93408277		Lim	

2	93495708	93495708	Her	
2	93549360	93549360	Lim	
2	93951461	93951461	Lim	
2	94072308	94072308	Bro	
2	94072308	94072308	Hol	
2	94260936	94260936	Nor	
2	95199905	95199905	Ang	
2	96082524	96082524	Hol	
2	96961746	96961746	Nor	
2	97080357	97080357	Jer	
2	101875760	101875760	Her	
2	102538954	103096368		Gir
2	103663439	103663439	Bro	
2	103707675	103707675	Jer	
2	103848414	103848414	Jer	
2	104540939	104540939	Lim	
2	104958840	105258724		Nel
2	105040403	105040403	Her	
2	105042095	105178397		Guz
2	105158290	105158290	Jer	
2	105666691	105666691	Gue	
2	105748575	105748575	Nor	
2	106925004	106925004	Hol	
2	107472555	107472555	Her	
2	107524304	107524304	Ang	
2	108181274	108181274	Hol	
2	108362982	108362982	Hol	
2	108495497	108495497	Her	
2	108913997	108913997	Ang	
2	110703049	110703049	Gue	
2	112110103	112110103	Ang	
2	112227843	112227843	Jer	
2	114067552	114067552	Jer	
2	116128218	116128218	Jer	
2	117210902	117210902	Ang	
2	117305584	117305584	Gue	
2	117451325	117502424	Cha	
2	117531904	117531904	Lim	
2	117686433	117686433	Bro	
2	117686433	117686433	Hol	
2	118500317	118500317	Ang	
2	118591144	118591144	Lim	
2	118947575	118947575	Ang	
2	118994262	118994262	Bro	
2	119122938	119122938	Ang	
2	119293733	120037582	Cha	
2	119350819	119350819	Bro	
2	119350819	119350819	Gue	

2	119452002	119546677	Lim	
2	119668056	119965520		Nel
2	119708912	119946550		Guz
2	119870309	119870309	Lim	
2	120751088	120751088	Gue	
2	120784862	120784862	Bro	
2	120993778	120993778	Lim	
2	121707643	121733389	Lim	
2	121757818	121757818	Ang	
2	121757818	121895125	Cha	
2	121757818	121757818	Hol	
2	121757818	121757818	Jer	
2	121954509	121954509	Lim	
2	123217794	123217794	Bro	
2	126050590	126050590	Hol	
2	127206815	127206815	Bro	
2	127206815	127263801	Hol	
2	127680352	127680352	Lim	
2	128021742	128021742	Her	
2	128451474	128451474	Lim	
2	128606233	128826190		Nel
2	128915082	128915082	Hol	
2	130843893	130843893	Hol	
2	131320980	131320980	Hol	
2	131817068	131817068	Nor	
2	132582699	132582699	Hol	
2	133482329	133482329	Jer	
2	133523656	133523656	Hol	
2	134481652	134481652	Ang	
2	135241531	135241531	Her	
2	135864028	135864028	Her	
3	452620	452620	Bro	
3	565368	565368	Jer	
3	939853	939853	Jer	
3	1200810	1200810	Gue	
3	3071606	3071606	Gue	
3	4099239	4099239	Bro	
3	4099239	4099239	Jer	
3	4099239	4099239	Lim	
3	4278285	4278285	Nor	
3	4487413	4487413	Bro	
3	4867336	4867336	Ang	
3	5577229	5577229	Her	
3	6845205	6845205	Nor	
3	7749394	7749394	Ang	
3	7874948	7874948	Bro	
3	9631023	9631023	Jer	
3	9997905	9997905	Bro	

3	10240563	10240563		Bro
3	10737892	10737892		Her
3	11037495	11037495		Her
3	11107155	11107155		Gue
3	11142773	11142773		Her
3	11594566	11594566		Hol
3	15883697	15883697		Gue
3	16390961	16390961		Nor
3	19689648	20166059	ZT	
3	20065439	20065439		Hol
3	20065439	20065439		Nor
3	20207243	20207243		Bro
3	21925914	21925914		Her
3	21925914	21925914		Jer
3	22362469	22362469		Lim
3	22509000	22509000		Hol
3	23791827	23791827		Lim
3	24350459	24350459		Lim
3	24487867	24597335		Cha
3	25074163	25074163		Lim
3	27594669	27594669		Hol
3	28054460	28054460		Her
3	28116948	28116948		Bro
3	29329346	29329346		Bro
3	30331825	30331825		Ang
3	31641866	31641866		Lim
3	32076326	32076326		Hol
3	32342257	32342257		Lim
3	32416106	32416106		Bro
3	32416106	32416106		Cha
3	32537088	32620178		Cha
3	32731219	32731219		Cha
3	32986013	32986013		Her
3	34208191	34208191		Nor
3	34424540	34424540		Gue
3	34576718	34576718		Bro
3	35785053	35785053		Hol
3	36469616	36469616		Her
3	36469616	36469616		Hol
3	37996416	37996416		Gue
3	38048428	38048428		Hol
3	38241587	38241587		Her
3	39547004	39547004		Jer
3	40130621	40130621		Jer
3	40535360	40535360		Ang
3	40950927	40950927		Hol
3	43009131	43009131		Jer
3	43749668	43749668		Lim

3	43831677	43831677	Nor	
3	43980009	43980009	Lim	
3	45518641	45518641	Jer	
3	46721234	46721234	Gue	
3	47279869	47279869	Lim	
3	47471386	47471386	Bro	
3	48631933	48631933	Gue	
3	49592722	49592722	Jer	
3	49665930	49665930	Lim	
3	53997758	53997758	Jer	
3	54731814	54731814	Jer	
3	55627105	55700001		Nel
3	55629068	55629068	Gue	
3	55832709	55832709		Nel
3	56084554	56084554	Nor	
3	56787867	56787867	Bro	
3	57159614	57159614	Her	
3	57809978	57809978	Hol	
3	58231701	58231701	Lim	
3	58953004	58953004	Bro	
3	59423265	59423265	Bro	
3	60680636	60680636	Hol	
3	63049356	63049356	Ang	
3	63398273	63398273	Jer	
3	63724726	63724726	Jer	
3	64607605	64607605	Her	
3	64772318	64772318	Ang	
3	65422854	65422854	Her	
3	67942605	67942605	Her	
3	70955109	70955109	Gue	
3	71095209	71095209	Lim	
3	73081911	73081911	Ang	
3	73455740	73455740	Gue	
3	73739712	73739712	Lim	
3	74075284	74075284	Nor	
3	74471839	74471839	Her	
3	75082992	75082992	Her	
3	75450949	75450949	Bro	
3	75688684	75688684	Jer	
3	77727223	77727223	Her	
3	78788446	78788446	Hol	
3	78832116	78832116	Lim	
3	80028642	80028642	Lim	
3	81172014	81172014	Lim	
3	81597965	81597965	Hol	
3	81634346	81634346	Her	
3	81777433	81777433	Her	
3	83413464	83413464	Her	

3	84920929	84920929		Hol
3	87595926	87595926		Bro
3	87808481	87808481		Ang
3	88899431	88899431		Nor
3	90386098	90386098		Bro
3	91328969	91328969		Ang
3	91328969	91328969		Hol
3	92050686	92050686		Lim
3	93666280	93666280		Gue
3	93985538	93985538		Jer
3	94063731	94063731		Bro
3	94742479	95401345	ZT	
3	96021243	96021243		Ang
3	97099662	97099662		Nor
3	97244701	97244701		Ang
3	97863924	97863924		Ang
3	97981877	97981877		Hol
3	98095410	98095410		Her
3	98222548	98222548		Nor
3	98904556	98904556		Hol
3	99312179	99312179		Her
3	102282008	102282008		Bro
3	103376036	103376036		Jer
3	103480386	103480386		Ang
3	103637948	103637948		Her
3	104385665	104385665		Hol
3	104566106	104566106		Jer
3	105017393	105017393		Jer
3	105942016	105942016		Lim
3	105989537	105989537		Bro
3	106490874	106490874		Hol
3	106845577	106845577		Hol
3	106948502	106948502		Gue
3	108243606	108243606		Gue
3	109566974	109566974		Lim
3	110374505	110374505		Her
3	110689018	110689018		Hol
3	111197176	111197176		Jer
3	111492824	111492824		Lim
3	111557162	111557162		Nor
3	112528099	112528099		Her
3	112853018	112853018		Ang
3	113625978	113625978		Nor
3	114578646	114578646		Her
3	115195756	115195756		Lim
3	115727220	115727220		Jer
3	115917466	115917466		Nor
3	116551733	116551733		Ang

3	117104133	117104133		Ang
3	117434023	117526423		Nor
3	117477829	117477829		Lim
3	117602026	117602026		Bro
3	119177155	119177155		Jer
3	120250131	120250131		Ang
3	124586031	124586031		Jer
4	2729422	2729422		Jer
4	3196019	3196019		Nor
4	3700272	3700272		Bro
4	3930202	3930202		Bro
4	5061724	5061724		Hol
4	6298054	6298054		Hol
4	7708368	7708368		Jer
4	8338571	8408268		Gue
4	9105532	9105532		Gue
4	10720132	10720132		Jer
4	11477947	11477947		Nor
4	12106878	12361811	ZT	
4	12657845	12657845		Ang
4	12657845	12657845		Hol
4	12657845	12657845		Jer
4	13098702	13098702		Hol
4	14831474	14831474		Nor
4	15186402	15186402		Nor
4	15329081	15329081		Nor
4	15423695	15423695		Lim
4	15575489	15575489		Her
4	15689489	15689489		Hol
4	15909646	15909646		Her
4	17487722	17487722		Lim
4	17568532	17568532		Jer
4	18514743	18514743		Nor
4	18977629	18977629		Gue
4	20786756	20786756		Bro
4	21024200	21024200		Her
4	21182534	21182534		Lim
4	21924904	21924904		Nor
4	22856027	22856027		Gue
4	23054302	23054302		Lim
4	23406784	23406784		Nor
4	23747430	23747430		Gue
4	24001957	24001957		Ang
4	26676918	26676918		Nor
4	27413627	27413627		Gue
4	28512491	28512491		Jer
4	29613879	29613879		Nor
4	30372172	30372172		Hol

4	30760882	30760882		Gue	
4	32802556	32802556		Bro	
4	32968840	32968840		Nor	
4	35964068	36058064			Nel
4	36012109	36012109		Hol	
4	36134798	36134798		Gue	
4	38380207	38380207		Gue	
4	40298925	40298925		Nor	
4	40726093	40726093		Her	
4	42080076	42080076		Jer	
4	43621021	43621021		Nor	
4	44424562	44424562		Gue	
4	46183425	46183425		Her	
4	46670940	46814875	ZT		
4	47258707	47258707		Gue	
4	48054791	48054791		Gue	
4	49349163	49349163		Her	
4	49385543	49385543		Lim	
4	49740381	49740381		Jer	
4	50167830	50167830		Nor	
4	50293840	50293840		Her	
4	52026595	52026595		Her	
4	52954149	52954149		Hol	
4	53001038	53001038		Nor	
4	53122964	53122964		Hol	
4	53122964	53122964		Jer	
4	53896224	53896224		Hol	
4	54074463	54074463		Jer	
4	55050570	55134721			Nel
4	55257636	55443618			Nel
4	55564014	55653930			Nel
4	55912717	55912717		Her	
4	56315518	56404051			Nel
4	56475874	56475874		Hol	
4	56505005	56619245			Nel
4	56561489	56561489		Nor	
4	56875012	56875012		Nor	
4	57121371	57121371		Hol	
4	57874268	57874268		Jer	
4	58599517	58599517		Gue	
4	59596296	59596296		Nor	
4	61609813	61609813		Hol	
4	63377248	63377248		Bro	
4	63528542	63528542		Lim	
4	64926156	64926156		Gue	
4	65351239	65351239		Bro	
4	65351239	65351239		Hol	
4	65351239	65351239		Lim	

4	65469531	65469531	Her
4	67726145	67726145	Gue
4	68247759	68247759	Hol
4	68340015	68340015	Lim
4	69528746	69528746	Hol
4	70147152	70147152	Gue
4	70289114	70289114	Lim
4	70632396	70632396	Nor
4	71507654	71507654	Gue
4	71671560	71671560	Her
4	73518942	73518942	Her
4	75598840	75598840	Her
4	76141626	76141626	Bro
4	77730993	77730993	Hol
4	78061802	78061802	Jer
4	78704761	78704761	Lim
4	79324525	79324525	Gue
4	80381215	80381215	Lim
4	80595579	80595579	Her
4	81637676	81637676	Ang
4	81637676	81637676	Nor
4	81797539	81797539	Lim
4	82298398	82298398	Ang
4	83061026	83061026	Hol
4	83061026	83061026	Nor
4	83168596	83168596	Ang
4	83574715	83604127	Gue
4	83697713	83697713	Jer
4	84320005	84320005	Bro
4	84626367	84626367	Her
4	87027941	87027941	Gue
4	87706457	87706457	Nor
4	88213063	88213063	Hol
4	89360938	89360938	Nor
4	89946180	89946180	Lim
4	90134841	90134841	Bro
4	93277289	93277289	Jer
4	95629588	95629588	Bro
4	96169933	96169933	Hol
4	97052759	97052759	Jer
4	97873655	97873655	Gue
4	98262275	98262275	Bro
4	99106053	99106053	Nor
4	99256469	99256469	Her
4	100161336	100161336	Hol
4	100259038	100259038	Nor
4	101976538	101976538	Ang
4	102085712	102085712	Gue

4	102213664	102213664	Nor
4	102827186	102827186	Nor
4	103519543	103519543	Ang
4	104102374	104102374	Her
4	105905928	105905928	Lim
4	106222170	106222170	Nor
4	107096645	107148814	Her
4	108177949	108177949	Ang
4	108177949	108177949	Hol
4	108177949	108177949	Jer
4	108554118	108554118	Ang
4	108835578	108835578	Bro
4	108863093	108863093	Ang
4	111134506	111134506	Lim
4	111714075	111714075	Her
4	111800401	111800401	Gue
4	112262940	112262940	Nor
4	112321244	112321244	Her
4	112385885	112385885	Lim
4	112520776	112520776	Bro
4	112520776	112520776	Lim
4	112669025	112669025	Lim
4	112975792	112975792	Nor
4	114337692	114337692	Jer
4	115150661	115150661	Bro
4	115274669	115274669	Jer
4	116352975	116352975	Bro
4	116457483	116457483	Hol
4	116525761	116525761	Jer
4	116650274	116650274	Jer
4	117459963	117459963	Lim
4	117524821	117524821	Nor
4	118015333	118015333	Gue
4	119875926	119875926	Hol
5	532145	532145	Ang
5	1780028	1780028	Ang
5	2226415	2226415	Nor
5	6249289	6249289	Ang
5	11575122	11575122	Bro
5	12172499	12172499	Bro
5	12241848	12241848	Jer
5	12435713	12435713	Gue
5	12435713	12435713	Jer
5	13203664	13203664	Her
5	13261705	13261705	Bro
5	15062606	15062606	Lim
5	15147209	15147209	Ang
5	15524211	15524211	Hol

5	15561728	15561728	Bro	
5	15706452	15706452	Lim	
5	16978964	16978964	Ang	
5	16978964	16978964	Gue	
5	16978964	16978964	Hol	
5	17749424	17749424	Ang	
5	17749424	17749424	Hol	
5	17749424	17749424	Jer	
5	18373081	18373081	Jer	
5	20611530	20754669		Guz
5	20856908	21238822		Guz
5	20856908	20913188		Nel
5	21861283	21861283	Gue	
5	22445252	22445252	Jer	
5	22678637	22678637	Gue	
5	23352672	23352672	Lim	
5	24023395	24023395	Gue	
5	24195453	24195453	Hol	
5	24918518	25088771		Nel
5	27137119	27137119	Ang	
5	29511503	29511503	Bro	
5	29962857	29962857	Hol	
5	31068358	31068358	Hol	
5	31590800	32108697		Nel
5	31618030	31618030	Her	
5	31690859	31690859	Nor	
5	32151620	32151620	Nor	
5	33453639	33453639	Gue	
5	36507904	36677850		Nel
5	37736215	37736215	Bro	
5	39183182	39498441		Guz
5	39293694	39462835		Nel
5	40553720	40864734		Guz
5	40646030	40788257		Nel
5	41855583	41855583	Ang	
5	42276968	42276968	Lim	
5	42387005	42387005	Gue	
5	42810659	42810659	Lim	
5	42835886	43384479		Gir
5	43494691	43733326		Gir
5	43498222	43498222	Jer	
5	44346949	44346949	Ang	
5	45749110	45807839		Gir
5	46183975	46183975	Ang	
5	46183975	46183975	Hol	
5	46183975	46183975	Jer	
5	46317932	46317932	Her	
5	46516881	46516881	Hol	

5	46766079	47022326			Guz
5	47545721	47545721		Ang	
5	47545721	47545721		Gue	
5	47545721	47545721		Hol	
5	48229556	48336996	ZT		
5	49701652	49701652		Nor	
5	49783026	49783026		Her	
5	50009234	50549666			Guz
5	50326119	50549666			Nel
5	51245751	51358939			Gir
5	51276764	51276764		Her	
5	51276764	51276764		Jer	
5	51307855	51307855		Hol	
5	51531787	51818460			Gir
5	52127055	52127055		Nor	
5	52740959	52740959		Lim	
5	52806574	52806574		Nor	
5	53554507	53554507		Gue	
5	53733336	53733336		Hol	
5	55881766	56049790	ZT		
5	56187224	56801729	ZT		
5	56684875	56684875		Hol	
5	56809677	56899118			Gir
5	57010135	57335801			Gir
5	57175000	57335801			Nel
5	57254378	57335801			Guz
5	57475894	57754977			Guz
5	57475894	57643602			Nel
5	57937432	57937432		Bro	
5	58344936	58344936		Jer	
5	58480291	58480291		Hol	
5	58513997	58868784			Nel
5	58545475	58868784			Gir
5	59268092	59268092			Gir
5	59268092	59268092			Nel
5	59332478	59332478		Lim	
5	59686148	59768602			Gir
5	59686148	59768602			Nel
5	59942136	59942136			Gir
5	60090401	60090401			Gir
5	60214497	60214497			Gir
5	60241190	60241190		Gue	
5	60602065	60642347		Cha	
5	61552763	61552763		Hol	
5	61798086	61798086		Ang	
5	61827732	62260477			Gir
5	61870300	62438598			Nel
5	61896542	61969069		Hol	

5	61990458	62345270		Guz
5	62094189	62094189	Bro	
5	62094189	62094189	Hol	
5	62198999	62198999	Bro	
5	62198999	62198999	Hol	
5	62322675	62322675	Lim	
5	62932240	62932240	Her	
5	63171539	63171539	Bro	
5	65618331	65618331	Jer	
5	67239744	67239744		Gir
5	67341443	67854189		Gir
5	68037433	68037433	Gue	
5	68088287	69541923		Gir
5	69276358	69276358	Nor	
5	69648685	70077918		Gir
5	69745832	69745832	Jer	
5	70182071	70234777		Gir
5	70236734	70236734	Lim	
5	70278553	70278553	Jer	
5	70338965	70338965		Gir
5	70458662	70458662		Gir
5	70568914	70568914		Gir
5	70680437	71119866		Gir
5	71182243	71182243	Gue	
5	72117069	72117069	Ang	
5	72160661	72427500		Gir
5	72805361	72995040		Gir
5	73226825	73226825	Lim	
5	73632210	73632210	Jer	
5	74229804	74229804	Bro	
5	74229804	74229804	Gue	
5	75961587	75961587	Lim	
5	76102522	76102522	Ang	
5	76653989	76653989	Gue	
5	76653989	76653989	Nor	
5	77345145	77345145	Nor	
5	80052274	80052274	Ang	
5	80458550	80458550	Lim	
5	82548748	82548748	Hol	
5	82779995	82779995	Lim	
5	82951695	82951695	Bro	
5	85418307	85418307	Bro	
5	86704345	86704345	Her	
5	87508398	87508398	Bro	
5	87563709	87563709	Her	
5	88483657	88483657	Ang	
5	89339202	89339202	Her	
5	89512928	89512928	Nor	

5	91152852	91197726	Nor
5	91449572	91449572	Her
5	91467698	91467698	Ang
5	92403004	92403004	Jer
5	96070058	96070058	Jer
5	96430014	96430014	Hol
5	97652740	97652740	Ang
5	98393443	98393443	Nor
5	98802828	98802828	Jer
5	99341425	99341425	Lim
5	100226474	100226474	Gue
5	100821035	100821035	Bro
5	100934878	100934878	Ang
5	102689791	102689791	Hol
5	102689791	102689791	Jer
5	102760399	102760399	Gue
5	102826491	102826491	Ang
5	103104527	103104527	Her
5	103984545	103984545	Her
5	105116043	105116043	Hol
5	105173591	105173591	Bro
5	105361302	105361302	Lim
5	106744927	106744927	Gue
5	107041248	107041248	Lim
5	107209045	107209045	Lim
5	107671017	107671017	Lim
5	107704991	107704991	Bro
5	107704991	107946258	Cha
5	107916169	107916169	Lim
5	107977613	107977613	Hol
5	108172899	108172899	Lim
5	108400665	108400665	Gue
5	109376304	109376304	Jer
5	109711617	109711617	Bro
5	110046386	110046386	Gue
5	112825269	112825269	Ang
5	112906879	112906879	Gue
5	113163784	113163784	Ang
5	113163784	113163784	Gue
5	113491995	113491995	Her
5	113744682	113744682	Bro
5	113744682	113744682	Her
5	114699559	114699559	Hol
5	114958931	114958931	Lim
5	115171406	115171406	Hol
5	115308130	115308130	Gue
5	117917533	117917533	Jer
5	118571958	118571958	Hol

5	118719909	118719909	Gue	
5	118719909	118719909	Nor	
5	120403003	120403003	Nor	
5	120973535	120973535	Ang	
5	120973535	120973535	Hol	
6	684934	684934	Her	
6	1004585	1004585	Nor	
6	1405878	1405878	Gue	
6	4234476	4234476	Jer	
6	4837150	4837150	Nor	
6	6881832	6881832	Nor	
6	7426584	7426584	Bro	
6	8297644	8297644	Ang	
6	10287413	10287413	Her	
6	10369158	10369158	Bro	
6	10869692	11186217		Gir
6	10958234	10958234	Ang	
6	12693539	12693539	Jer	
6	12797614	12797614	Ang	
6	13132216	13132216	Lim	
6	16547650	16577676		Gir
6	16850753	16850753	Bro	
6	17237816	17886829		Guz
6	17512361	17886829		Nel
6	17544456	17544456	Her	
6	17758788	17758788	Hol	
6	18035285	18609964		Guz
6	18035285	18398817		Nel
6	18397886	18397886	Nor	
6	18495302	18495302	Jer	
6	18576650	18576650	Bro	
6	20658721	20658721	Nor	
6	20769990	20769990	Hol	
6	21034521	21034521	Her	
6	22577502	22577502	Lim	
6	23166766	23166766	Hol	
6	23429169	23429169	Bro	
6	24238233	24238233	Nor	
6	25648085	25648085	Jer	
6	25860295	25860295	Jer	
6	26500724	26500724	Gue	
6	27196752	27196752	Ang	
6	27927608	27927608	Nor	
6	28810002	28810002	Nor	
6	30181447	30181447	Nor	
6	30270520	30270520	Hol	
6	30679552	30679552	Ang	
6	30679552	30679552	Nor	

6	30751312	30751312	Jer
6	30893089	30893089	Gue
6	31210404	31210404	Ang
6	31671451	31671451	Jer
6	32067092	32067092	Bro
6	32208164	32208164	Bro
6	32605838	32605838	Hol
6	33302947	33302947	Bro
6	35787317	35787317	Her
6	35854675	35854675	Nor
6	35922378	35922378	Gue
6	37141253	37141253	Nor
6	38321808	38321808	Ang
6	38369497	38369497	Gue
6	38732667	38732667	Bro
6	39069719	39069719	Nor
6	40098301	40098301	Lim
6	40448848	40448848	Jer
6	40580921	40580921	Jer
6	41983400	41983400	Hol
6	42714287	42714287	Bro
6	43254403	43254403	Jer
6	43590568	43590568	Gue
6	43859732	43859732	Ang
6	43859732	43859732	Gue
6	44228217	44228217	Gue
6	44372550	44372550	Nor
6	44614641	44614641	Nor
6	44714358	44714358	Jer
6	45264193	45264193	Gue
6	46259614	46259614	Her
6	46556246	46556246	Ang
6	46931105	46931105	Her
6	47612084	47612084	Jer
6	47743983	47743983	Nor
6	47999518	47999518	Nor
6	48054147	48054147	Her
6	48171183	48171183	Gue
6	48755138	48755138	Jer
6	49942445	49942445	Hol
6	50407385	50407385	Gue
6	55830448	55830448	Hol
6	56079069	56079069	Nor
6	57005400	57005400	Bro
6	57331206	57331206	Her
6	57679318	57679318	Bro
6	58411107	58411107	Gue
6	58679474	58679474	Nor

6	58893067	58893067	Her	
6	59358794	59358794	Nor	
6	60513152	60513152	Hol	
6	62144436	62144436	Jer	
6	64071026	64071026	Bro	
6	64428159	64775155		Gir
6	65363458	65363458		Gir
6	65491643	65917106		Gir
6	65578924	65578924	Bro	
6	65578924	65578924	Hol	
6	66029830	67324212		Gir
6	66228416	66228416	Nor	
6	66554508	66554508	Her	
6	66840522	66840522	Bro	
6	67726209	68650753		Gir
6	67865555	67865555	Lim	
6	67965922	68615393		Guz
6	68551449	68551449	Bro	
6	68579990	69047811	Cha	
6	68848548	68848548	Bro	
6	68848548	68848548	Gue	
6	68952101	68952101	Lim	
6	69246461	69879993	Cha	
6	69655525	69655525	Bro	
6	69655525	69655525	Gue	
6	69772367	69772367	Bro	
6	69772367	69772367	Gue	
6	69832241	69832241	Lim	
6	69924213	70108109		Gir
6	69924213	69924213	Gue	
6	70074322	70074322	Hol	
6	70142390	70142390	Bro	
6	70142390	71046728	Cha	
6	70250655	70250655	Nor	
6	70304717	70304717	Bro	
6	70304717	70304717	Gue	
6	70369018	70729075		Guz
6	70397767	70421237	Ang	
6	70552634	70552634	Bro	
6	70552634	70552634	Gue	
6	70663881	70704748	Lim	
6	70777638	70777638	Bro	
6	70777638	70777638	Gue	
6	70825726	70825726	Ang	
6	70878324	70909347	Bro	
6	70878324	70909347	Gue	
6	71111976	71168557		Gir
6	71289939	71709066		Gir

6	71289939	71709066		Guz
6	71293044	71293044	Nor	
6	71394063	71709066		Nel
6	71432119	71453049	Lim	
6	71453049	71453049	Jer	
6	71849761	72247851		Gir
6	71849761	72755638		Guz
6	71849761	72023200		Nel
6	72589945	72589945	Lim	
6	72649477	72812225		Nel
6	72652172	72652172	Hol	
6	72736904	72907971	Cha	
6	72736904	72736904	Her	
6	72837455	72907971	Nor	
6	73878270	73878270	Gue	
6	74672294	74672294	Bro	
6	75337457	75337457	Her	
6	75539962	75615442	Cha	
6	75615442	75615442	Gue	
6	76343192	76343192	Gue	
6	76717175	76717175	Lim	
6	77016454	77016454	Ang	
6	77016454	77016454	Gue	
6	77016454	77016454	Hol	
6	77170401	77170401	Gue	
6	77458333	77458333	Lim	
6	77654694	77654694	Her	
6	77770651	77770651	Bro	
6	78106873	78421672		Nel
6	78818101	78818101	Jer	
6	80303879	80303879	Lim	
6	81086466	81086466	Bro	
6	81195717	81195717	Ang	
6	81362585	81362585	Gue	
6	82395727	82395727	Gue	
6	83419616	83419616	Jer	
6	83799011	83799011	Lim	
6	83988527	83988527	Lim	
6	84851488	84851488	Jer	
6	84874750	84874750	Lim	
6	85093387	85093387	Her	
6	85452843	85452843	Ang	
6	86210585	86210585	Hol	
6	87009162	87009162	Gue	
6	87528247	87528247	Lim	
6	88085965	88085965	Gue	
6	88407205	88407205	Bro	
6	88973755	88973755	Bro	

6	88973755	89110574	Cha	
6	89357777	89357777	Hol	
6	89357777	89357777	Jer	
6	89586128	89586128	Lim	
6	90486780	90486780	Her	
6	90695950	90695950	Gue	
6	90816708	90816708	Her	
6	92623916	92623916	Jer	
6	93107752	93107752	Nor	
6	93661955	93661955	Lim	
6	95701043	95701043	Her	
6	95868577	95868577	Lim	
6	96133577	96133577	Gue	
6	97048566	97048566	Hol	
6	97295497	97295497	Her	
6	97874422	97874422	Hol	
6	98620384	98620384	Nor	
6	99741981	99741981	Her	
6	102043097	102043097	Lim	
6	102354018	102354018	Ang	
6	102656425	102656425	Her	
6	103684904	103684904	Gue	
6	103684904	103684904	Jer	
6	104147101	104147101	Her	
6	104428788	104428788	Ang	
6	104896770	104896770	Hol	
6	104995710	104995710	Ang	
6	105082901	105082901	Bro	
6	105282590	105282590	Nor	
6	105476912	105476912	Lim	
6	105647257	105647257	Nor	
6	106195779	106195779	Jer	
6	106953242	106953242	Hol	
6	106953242	106953242	Jer	
6	107423566	107423566	Her	
6	108140878	108140878	Bro	
6	108355687	108355687	Ang	
6	109897928	109897928	Hol	
6	109997823	110151219		Gir
6	110099567	110099567	Lim	
6	110262552	110336208	Jer	
6	110452712	110898941	Cha	
6	110656909	110656909	Ang	
6	111040377	111040377	Gue	
6	111132017	111132017	Her	
6	111382589	111382589	Bro	
6	111448459	111448459	Gue	
6	111532693	111532693	Ang	

6	111532693	111532693		Hol
6	111737970	111737970		Jer
6	113194494	113194494		Bro
6	113405516	113405516		Jer
6	114223059	114223059		Ang
6	114626046	114626046		Bro
6	114626046	114626046		Gue
6	114709329	114709329		Ang
6	114709329	114709329		Hol
6	116433715	116433715		Ang
6	117097825	117097825		Jer
6	117514172	117514172		Bro
6	119189552	119189552		Her
6	119189552	119189552		Hol
7	3408345	3408345		Nor
7	3764385	3764385		Her
7	4408245	4408245		Ang
7	5535767	5535767		Her
7	5670723	5670723		Her
7	6391481	6391481		Ang
7	8957328	8957328		Her
7	9574967	9574967		Her
7	12396999	12396999		Nor
7	14437027	14437027		Jer
7	14591281	14591281		Gue
7	14950181	14950181		Gue
7	15238915	15238915		Jer
7	15824296	15824296		Ang
7	16377983	16377983		Her
7	17567301	17567301		Gue
7	18043992	18043992		Bro
7	19347072	19347072		Hol
7	19752701	19752701		Hol
7	19752701	19752701		Nor
7	21008805	21606667	ZT	
7	21545270	21545270		Gue
7	21663575	21663575		Gue
7	21732061	21732061		Lim
7	21979132	21979132		Hol
7	22936186	22936186		Jer
7	23078152	23078152		Gue
7	24589644	24589644		Jer
7	25638909	25638909		Jer
7	26211382	26211382		Lim
7	26418683	26418683		Nor
7	27694291	27694291		Bro
7	27746668	27746668		Gue
7	28079542	28079542		Nor

7	28188631	28188631		Lim	
7	28352637	28352637		Her	
7	29823611	29823611		Lim	
7	30269254	30269254		Gue	
7	30776329	30776329		Ang	
7	30916696	30916696		Ang	
7	31048900	31048900		Ang	
7	32244753	32244753		Nor	
7	32299740	32299740		Jer	
7	32356476	32441261			Nel
7	32362945	32362945		Ang	
7	32553636	32553636			Nel
7	32565544	32565544		Nor	
7	33072433	33072433		Gue	
7	35583298	35727864			Nel
7	35646155	35646155		Hol	
7	35801918	35801918		Hol	
7	35801918	35801918		Nor	
7	37064981	37064981		Nor	
7	37620911	37620911		Her	
7	39408588	39408588		Hol	
7	41560760	41560760		Gue	
7	41800624	41800624		Nor	
7	41988894	41988894		Jer	
7	45456287	45456287		Hol	
7	46821712	46821712		Lim	
7	47299497	47859329	ZT		
7	47559314	47559314		Lim	
7	48465451	48465451		Bro	
7	48465451	48465451		Hol	
7	48531566	48531566		Lim	
7	48675923	48675923		Gue	
7	48976487	49339126		Cha	
7	48976487	48976487		Gue	
7	49043281	49043281		Ang	
7	49339126	49339126		Lim	
7	49604191	49604191		Gue	
7	49757631	49757631		Her	
7	50000591	50000591		Lim	
7	50083726	50083726		Gue	
7	50207714	50207714		Hol	
7	50359286	50359286		Jer	
7	50417918	50417918		Hol	
7	50945085	50945085		Bro	
7	50945085	50945085		Hol	
7	50951861	52544266	ZT		
7	51007922	51273470		Cha	
7	51065526	51065526		Gue	

7	51923355	52518362			Gir
7	51938829	51938829		Nor	
7	52676102	53757384	ZT		
7	53314454	53738523		Cha	
7	53627815	53627815		Ang	
7	53984004	54160135			Guz
7	55413032	55855334		Cha	
7	55560135	55560135		Jer	
7	55657740	55869106			Guz
7	55699270	55699270		Gue	
7	55699899	55869106			Nel
7	55723868	55723868		Jer	
7	55990245	56063642			Guz
7	55990245	56038711			Nel
7	56636311	56636311		Lim	
7	57015697	57015697		Jer	
7	60413422	60413422		Gue	
7	60930457	60930457		Gue	
7	60930457	60930457		Nor	
7	61142271	61142271		Her	
7	61220348	61220348		Hol	
7	61658196	61658196		Nor	
7	62026556	62026556		Lim	
7	62951920	62951920		Lim	
7	63319085	63319085		Hol	
7	63567466	63567466		Bro	
7	64315456	64315456		Ang	
7	64513354	64513354		Gue	
7	64714612	64714612		Nor	
7	67576213	67576213		Nor	
7	68201746	68201746		Bro	
7	68262706	68262706		Jer	
7	68702530	68702530		Nor	
7	69243391	69243391		Lim	
7	69403803	69403803		Her	
7	69507347	69507347		Gue	
7	70988849	70988849		Her	
7	71089100	71089100		Lim	
7	71293075	71293075		Hol	
7	71697239	71697239		Hol	
7	72563514	72563514		Her	
7	72899435	72899435		Jer	
7	73155099	73155099		Jer	
7	74568754	74568754		Nor	
7	75825484	75825484		Her	
7	76994051	76994051		Bro	
7	77027187	77027187		Lim	
7	77141024	77141024		Bro	

7	77725073	77725073	Hol
7	77725073	77725073	Nor
7	80553574	80553574	Ang
7	81706317	81706317	Nor
7	82391192	82391192	Lim
7	82756535	82756535	Bro
7	82795447	82795447	Gue
7	83646180	83646180	Bro
7	84023632	84023632	Jer
7	84561462	84561462	Hol
7	84693489	84693489	Bro
7	85408031	85408031	Lim
7	86484464	86484464	Lim
7	86812524	86812524	Her
7	87000448	87000448	Bro
7	87481184	87481184	Hol
7	87681984	87681984	Nor
7	88413966	88413966	Ang
7	88632985	88632985	Lim
7	90358476	90358476	Bro
7	91619336	91619336	Ang
7	91619336	91619336	Hol
7	92639709	92639709	Ang
7	92685811	92685811	Lim
7	92950866	92950866	Gue
7	94052461	94052461	Bro
7	94796489	94796489	Ang
7	95720431	95720431	Ang
7	96523316	96523316	Bro
7	97732454	97732454	Bro
7	97954254	97954254	Lim
7	98044495	98044495	Nor
7	98711109	98711109	Ang
7	98893611	98893611	Jer
7	99177441	99177441	Her
7	99967071	99967071	Bro
7	100270303	100270303	Nor
7	100935977	100935977	Her
7	102320223	102320223	Her
7	103371336	103371336	Gue
7	104127595	104127595	Lim
7	104241239	104241239	Lim
7	104290665	104290665	Bro
7	104402974	104402974	Lim
7	106734846	106734846	Gue
7	107551125	107551125	Lim
7	107939136	107939136	Jer
7	108807769	108807769	Hol

7	109138859	109138859	Jer	
7	109285657	109285657	Bro	
7	109697318	109697318	Her	
7	110125602	110125602	Ang	
7	110125602	110125602	Jer	
7	110744040	110744040	Hol	
7	111090063	111090063	Her	
7	111527301	111527301	Nor	
7	112021719	112021719	Bro	
8	227611	356238		Nel
8	807843	807843	Jer	
8	1393934	1393934	Jer	
8	2400846	2400846	Bro	
8	4207450	4207450	Her	
8	5083689	5083689	Her	
8	5580213	5580213	Ang	
8	6088202	6088202	Lim	
8	6444437	6444437	Her	
8	7456372	7456372	Her	
8	7456372	7456372	Jer	
8	7745692	7745692	Bro	
8	9290785	9290785	Gue	
8	9615972	9657727		Gir
8	10383387	10383387	Nor	
8	11383695	11383695	Lim	
8	11658773	11658773	Jer	
8	11713177	11713177	Her	
8	13148140	13148140	Jer	
8	15848671	15848671	Bro	
8	15996735	15996735	Hol	
8	16910644	16910644	Ang	
8	17446440	17446440	Hol	
8	17486491	17486491	Bro	
8	19208229	19208229	Her	
8	20329496	20329496	Gue	
8	22308841	22308841	Ang	
8	22919820	22919820	Her	
8	23168545	23168545	Jer	
8	23290934	23290934	Her	
8	23619430	23619430	Bro	
8	23789303	23789303	Bro	
8	23984036	23984036	Nor	
8	24618142	24618142	Hol	
8	25261181	25261181	Jer	
8	26718343	26718343	Hol	
8	28079584	28079584	Nor	
8	28471805	28471805	Bro	
8	28687578	28872401		Guz

8	28687578	28872401			Nel
8	28763870	28763870		Gue	
8	28825152	28825152		Her	
8	29449439	29449439		Ang	
8	29647443	29647443		Ang	
8	29838160	30090854			Gir
8	29922096	29922096		Bro	
8	30025994	30090854			Nel
8	30443348	30443348		Ang	
8	30628097	30842868			Guz
8	30644755	30644755		Gue	
8	30715351	30715351		Bro	
8	30799143	30799143		Gue	
8	30799143	30799143		Her	
8	32085661	32085661		Hol	
8	34583250	34583250		Gue	
8	36477188	36477188		Her	
8	37222756	37222756		Nor	
8	37342100	37342100		Ang	
8	37388481	37388481		Bro	
8	37822309	37822309		Bro	
8	38070407	38070407		Ang	
8	39188107	39188107		Lim	
8	39288115	39689110	ZT		
8	39800492	39800492	ZT		
8	40111441	40111441		Jer	
8	40570499	40570499		Bro	
8	40858649	40858649		Lim	
8	41089930	41089930		Jer	
8	41960719	41960719		Jer	
8	42033636	42033636		Bro	
8	42126519	42126519		Ang	
8	42126519	42126519		Hol	
8	42228790	42228790		Hol	
8	43102448	43102448		Her	
8	45755851	45755851		Lim	
8	45838096	45838096		Bro	
8	45903217	45903217		Her	
8	45903217	45903217		Jer	
8	46097338	46097338		Hol	
8	46097338	46097338		Jer	
8	47036986	47036986		Jer	
8	47195421	47195421		Nor	
8	48646578	48646578		Hol	
8	48744904	48809204		Bro	
8	51243026	51243026		Bro	
8	52638344	52638344		Jer	
8	52999092	52999092		Her	

8	53490845	54592381	ZT		
8	53840928	53840928		Nor	
8	55586698	55586698		Nor	
8	57499577	57499577		Nor	
8	57743546	57743546		Her	
8	58401933	58469030			Nel
8	58649674	58727004	ZT		
8	58852794	58852794		Hol	
8	60586901	60634349			Nel
8	61012058	61012058		Ang	
8	61543379	62082735	ZT		
8	62223829	62874750	ZT		
8	63144428	63144428		Gue	
8	63563140	63661095		Gue	
8	63934537	63934537		Hol	
8	64077413	64077413		Hol	
8	66171277	66171277		Jer	
8	66593971	66593971		Jer	
8	66757742	66990971			Guz
8	67156577	67156577		Ang	
8	68238670	68238670		Ang	
8	68238670	68238670		Nor	
8	68915433	68915433		Nor	
8	69691214	70488061	ZT		
8	70228036	70228036		Ang	
8	70344673	70344673		Her	
8	70801131	70801131		Bro	
8	71503728	71503728		Jer	
8	71985952	71985952		Gue	
8	72460748	72460748		Lim	
8	73230618	73230618		Jer	
8	73552298	73552298		Jer	
8	73617355	73704634	ZT		
8	75100951	75171282			Nel
8	75889454	76670107			Nel
8	76319321	76319321		Her	
8	76797897	76833951			Nel
8	77319956	77319956		Gue	
8	77646612	77646612		Nor	
8	77917690	77917690		Ang	
8	79269699	79269699		Hol	
8	82371836	82371836		Jer	
8	83705825	83705825		Bro	
8	84132219	84132219		Lim	
8	85657033	85657033		Bro	
8	88419753	88419753		Jer	
8	88894493	88894493		Ang	
8	90107228	90107228		Gue	

8	92009341	92009341	Hol
8	92797782	92797782	Ang
8	92797782	92797782	Jer
8	93263312	93263312	Gue
8	93363018	93363018	Ang
8	93576985	93576985	Her
8	93832928	93832928	Bro
8	94893598	94893598	Bro
8	95194896	95194896	Her
8	95301329	95301329	Lim
8	95713968	95713968	Ang
8	95829528	95829528	Hol
8	96069029	96069029	Nor
8	96128989	96128989	Her
8	96292160	96292160	Bro
8	97981732	97981732	Gue
8	98888929	98888929	Her
8	99591497	99591497	Nor
8	100184842	100184842	Her
8	101627593	101627593	Hol
8	103695388	103695388	Jer
8	104111627	104111627	Jer
8	104425148	104425148	Bro
8	104507052	104507052	Jer
8	104994753	104994753	Nor
8	106029979	106029979	Nor
8	106172780	106172780	Ang
8	108048851	108048851	Bro
8	108128729	108128729	Hol
8	109067870	109067870	Lim
8	109882809	109882809	Ang
9	690802	690802	Lim
9	3684547	3684547	Hol
9	3796382	3796382	Bro
9	3796382	4191092	Cha
9	4007054	4007054	Bro
9	4129332	4129332	Hol
9	4305338	4362391	Cha
9	4305338	4305338	Lim
9	4873335	4873335	Jer
9	5176318	5176318	Gue
9	5228930	5228930	Nor
9	5772412	5772412	Ang
9	6535846	6535846	Hol
9	6700692	6700692	Jer
9	7200551	7200551	Bro
9	7985903	7985903	Gue
9	8012538	8012538	Jer

9	8640019	8640019	Bro	
9	8842006	8842006	Gue	
9	9402427	9402427	Nor	
9	11124315	11124315	Jer	
9	11337983	11337983	Hol	
9	12070981	12070981	Gue	
9	12226204	12226204	Nor	
9	12599151	12599151	Bro	
9	13303881	13303881	Nor	
9	15193951	15193951	Bro	
9	15893918	15893918	Lim	
9	16416954	16416954	Bro	
9	17425045	17425045	Bro	
9	17425045	17425045	Gue	
9	18700553	18700553	Lim	
9	19074860	19074860	Lim	
9	19407892	19407892	Lim	
9	19638679	19638679	Ang	
9	19816668	19816668	Lim	
9	20123664	20123664	Her	
9	20123664	20123664	Jer	
9	20563228	20563228	Lim	
9	21663298	21663298	Ang	
9	22358470	22358470	Nor	
9	22638267	22638267	Her	
9	23170155	23170155	Hol	
9	23221513	23221513	Gue	
9	23307499	23307499	Lim	
9	23371579	23371579	Bro	
9	23482919	23482919	Bro	
9	23482919	23482919	Hol	
9	23751786	23964598		Guz
9	24015001	24015001	Ang	
9	24120158	24120158	Lim	
9	24707581	24707581	Her	
9	25387468	25387468	Hol	
9	25770398	25964541		Guz
9	26459366	26459366	Nor	
9	28195699	28195699	Ang	
9	28195699	28195699	Gue	
9	28195699	28195699	Hol	
9	28302578	28302578	Jer	
9	29502871	29502871	Her	
9	30620057	30620057	Jer	
9	31028182	31028182	Jer	
9	32346476	32346476	Bro	
9	32827869	32827869	Jer	
9	33744291	33744291	Her	

9	35602819	35602819	Bro	
9	36285976	36285976	Gue	
9	36285976	36285976	Nor	
9	36315157	36315157	Lim	
9	36962229	37067407		Gir
9	38196189	38196189	Her	
9	38251741	38251741	Gue	
9	40082884	40287003		Guz
9	40389526	40389526	Lim	
9	40400556	40676359		Guz
9	40524828	40691358	Cha	
9	40845265	40845265	Gue	
9	40845265	40845265	Nor	
9	41873404	41873404	Gue	
9	42056117	42056117	Her	
9	42231014	42231014	Jer	
9	42308530	42866215		Gir
9	42506424	42556097	Lim	
9	42800959	42800959	Ang	
9	42800959	42800959	Hol	
9	42852058	42852058	Lim	
9	42930839	43296117	Cha	
9	43296117	43296117	Bro	
9	45545519	45767377	Cha	
9	46118697	46118697	Her	
9	46160692	46160692	Ang	
9	46910116	47250608	Cha	
9	47150686	47150686	Jer	
9	47354617	47354617	Ang	
9	47354617	47503904	Cha	
9	47392224	47392224	Gue	
9	47429976	47429976	Jer	
9	47882445	47882445	Gue	
9	48191836	48191836	Jer	
9	48233084	48233084	Lim	
9	49006798	49006798	Lim	
9	49010680	49326280		Guz
9	49382213	49382213	Bro	
9	49573660	49573660	Lim	
9	49800695	50235638		Gir
9	50225398	50225398	Nor	
9	50366177	50366177	Jer	
9	51165951	51165951	Her	
9	51617221	51617221	Lim	
9	51790511	51790511	Her	
9	52509936	52587905	Hol	
9	52556989	52873430		Nel
9	52587905	52587905	Ang	

9	52587905	52587905	Jer
9	52743323	52743323	Hol
9	53324392	53324392	Gue
9	55813477	55813477	Nor
9	55944849	55944849	Bro
9	60114332	60114332	Hol
9	60283742	60283742	Nor
9	61397847	61397847	Nor
9	61617079	61617079	Jer
9	61937112	61937112	Bro
9	62922027	62922027	Gue
9	63825670	63825670	Bro
9	63928374	63928374	Nor
9	64331474	64331474	Nor
9	64381905	64381905	Her
9	64593605	64593605	Ang
9	65485634	65485634	Hol
9	66171529	66171529	Nor
9	66541767	66541767	Lim
9	67110583	67110583	Lim
9	67889925	67889925	Lim
9	68611689	68611689	Bro
9	68611689	68611689	Gue
9	68773418	68773418	Jer
9	71798963	71979638	Lim
9	71827103	71827103	Bro
9	71914087	71914087	Gue
9	72201716	72201716	Hol
9	72201716	72201716	Jer
9	72291005	72291005	Gue
9	72985658	72985658	Bro
9	74410911	74410911	Jer
9	75595971	75595971	Ang
9	76188149	76188149	Lim
9	76534576	76534576	Gue
9	77760067	77760067	Her
9	78774222	78774222	Lim
9	79916244	79916244	Bro
9	80194380	80194380	Lim
9	80921402	80998297	Hol
9	81312423	81312423	Hol
9	81399462	81399462	Bro
9	81523056	81600085	Gue
9	81710664	81710664	Lim
9	81964703	81964703	Hol
9	81964703	81964703	Nor
9	82495072	82495072	Hol
9	83837068	83837068	Hol

9	84489855	84489855	Nor
9	85536454	85536454	Lim
9	85987860	85987860	Ang
9	86343243	86343243	Nor
9	86546451	86546451	Jer
9	86588370	86588370	Lim
9	86902322	86902322	Bro
9	87186800	87186800	Her
9	87542864	87542864	Ang
9	87855163	87855163	Jer
9	88033710	88033710	Hol
9	88366032	88366032	Ang
9	88366032	88366032	Her
9	88385591	88385591	Bro
9	88502597	88502597	Nor
9	90247980	90247980	Gue
9	90410552	90410552	Jer
9	90690991	90690991	Nor
9	91717629	91717629	Hol
9	93880473	93880473	Bro
9	96021779	96021779	Ang
9	96673261	96673261	Hol
9	96704827	96704827	Nor
9	97459242	97459242	Nor
9	97584669	97584669	Ang
9	98130070	98130070	Nor
9	99073790	99073790	Her
9	99931149	99931149	Nor
9	100382402	100382402	Her
9	100625191	100625191	Lim
9	101083060	101083060	Lim
9	101436616	101436616	Lim
9	101530740	101530740	Nor
9	101751589	101751589	Her
9	101824431	101824431	Hol
9	102443593	102443593	Hol
9	102573146	102573146	Ang
9	102573146	102573146	Nor
9	102838428	102838428	Lim
9	104154509	104154509	Nor
9	105649672	105649672	Her
10	134749	134749	Gue
10	134749	134749	Jer
10	2228385	2228385	Gue
10	2311581	2311581	Ang
10	2504463	2504463	Jer
10	2637641	2637641	Bro
10	2924279	2924279	Hol

10	3141092	3141092	Her	
10	3585430	3585430	Her	
10	3929654	3929654	Nor	
10	4469925	4469925	Ang	
10	5715632	5877794		Guz
10	6278469	6278469	Lim	
10	6403269	6403269	Bro	
10	6451551	6451551	Hol	
10	7261838	7261838	Bro	
10	7261838	7369851	Cha	
10	7392379	7392379	Hol	
10	7430222	7430222	Gue	
10	7835791	7835791	Gue	
10	8069320	8069320	Lim	
10	8329810	8329810	Her	
10	8395912	8494503	Lim	
10	8643228	8643228	Gue	
10	9363492	9456921	Cha	
10	11407194	11407194	Bro	
10	11998905	11998905	Her	
10	12216332	12216332	Jer	
10	12731297	12731297	Lim	
10	12883315	12883315	Hol	
10	12919154	12919154	Ang	
10	14605227	14605227	Ang	
10	15226746	15226746	Nor	
10	15437548	15437548	Gue	
10	15535737	15535737	Hol	
10	15756752	15756752	Ang	
10	15994229	15994229	Her	
10	16583007	16583007	Ang	
10	17127861	17127861	Bro	
10	17173220	17173220	Jer	
10	17544378	17544378	Bro	
10	18035832	18035832	Bro	
10	21871198	21871198	Gue	
10	22212977	22212977	Nor	
10	25629283	25629283	Ang	
10	26500270	26500270	Jer	
10	27125997	27125997	Ang	
10	27431672	27431672	Lim	
10	27711498	27711498	Bro	
10	29252807	29252807	Ang	
10	29282049	29282049	Gue	
10	29324665	29324665	Jer	
10	29398414	29398414	Ang	
10	29789876	29789876	Her	
10	31330749	31330749	Ang	

10	31503971	31503971		Ang	
10	34505253	34505253		Hol	
10	34708307	34708307		Bro	
10	35275282	35275282		Her	
10	36488829	37051537	ZT		
10	36751951	36751951		Her	
10	37810802	37810802		Nor	
10	38030548	38030548		Hol	
10	38262598	38262598		Bro	
10	41051430	41051430		Bro	
10	41727808	41727808		Hol	
10	44105865	44198451		Nor	
10	44825282	44825282		Jer	
10	47429340	47429340		Her	
10	47928667	47928667		Gue	
10	48058379	48058379		Her	
10	48558673	48558673		Ang	
10	48590596	48590596		Her	
10	48675935	48675935		Lim	
10	51377461	51377461		Ang	
10	51807323	51807323		Her	
10	53903255	53903255		Gue	
10	54925134	54925134		Bro	
10	54994071	54994071		Her	
10	55311677	55311677		Nor	
10	56304161	56304161		Lim	
10	56397326	56397326		Gue	
10	56443444	56443444		Lim	
10	56538370	56538370		Nor	
10	57588822	57588822		Nor	
10	57691818	57691818		Lim	
10	58121753	58121753		Bro	
10	59615280	59615280		Lim	
10	59927870	59927870		Gue	
10	60321912	60321912		Her	
10	60381852	60446128			Nel
10	60512444	60512444		Her	
10	60554234	60621528			Nel
10	62795166	62795166		Lim	
10	63025239	63025239		Lim	
10	63187437	63187437		Her	
10	63498887	63498887		Ang	
10	63498887	63498887		Gue	
10	63498887	63498887		Hol	
10	63953496	63953496		Gue	
10	65388858	65388858		Jer	
10	65923499	65923499		Lim	
10	66121449	66121449		Jer	

10	66704624	66704624	Nor	
10	68880888	68880888	Ang	
10	69352412	69352412	Gue	
10	69508613	69508613	Hol	
10	69988910	69988910	Bro	
10	70142738	70142738	Gue	
10	70231445	70231445	Ang	
10	70756526	70756526	Her	
10	70756526	70756526	Hol	
10	71421255	71421255	Bro	
10	71770156	71770156	Ang	
10	72809829	72809829	Nor	
10	73755051	73755051	Bro	
10	75007019	75007019	Bro	
10	75306249	75306249	Nor	
10	75509429	75509429	Lim	
10	75844063	75844063	Nor	
10	76057864	76057864	Hol	
10	76624088	76624088	Lim	
10	76777629	76777629	Her	
10	76832220	76832220	Lim	
10	77332937	77332937	Hol	
10	77332937	77332937	Jer	
10	77532253	77532253	Nor	
10	77788502	77788502	Hol	
10	78516979	78516979	Nor	
10	82904079	82904079	Gue	
10	83271286	83271286	Jer	
10	84447428	84447428	Ang	
10	85008546	85008546	Ang	
10	85008546	85008546	Nor	
10	86272394	86272394	Her	
10	87794200	87794200	Her	
10	88130360	88130360	Ang	
10	88130360	88130360	Gue	
10	88130360	88130360	Nor	
10	88834174	88834174	Hol	
10	89023133	89023133	Ang	
10	89023133	89023133	Nor	
10	89434017	89434017	Nor	
10	89782984	90050733		Gir
10	89945955	89945955	Her	
10	91970455	91970455	Hol	
10	92073773	92073773	Ang	
10	92602520	92602520	Jer	
10	92653880	92653880	Hol	
10	92790932	92790932	Jer	
10	93346551	93346551	Jer	

10	93509401	93509401	Ang	
10	93509401	93509401	Jer	
10	94715950	94715950	Gue	
10	96103382	96103382	Bro	
10	96388351	96388351	Ang	
10	97377127	97377127	Nor	
10	97649823	97649823	Ang	
10	98623937	98623937	Hol	
10	99308679	99308679	Ang	
10	99944011	99944011	Gue	
10	100228887	100228887	Her	
10	100270341	100270341	Ang	
10	100534627	100534627	Ang	
10	100770781	100770781	Gue	
10	101028982	101028982	Hol	
10	104145811	104145811	Ang	
11	393478	393478	Jer	
11	616630	616630	Jer	
11	663516	663516	Gue	
11	1412348	1412348	Her	
11	2041610	2041610	Ang	
11	3722186	3722186	Lim	
11	4415832	4415832	Hol	
11	4456531	4456531	Jer	
11	6006940	6006940	Nor	
11	6731396	6731396	Nor	
11	7107211	7107211	Jer	
11	7274240	7274240	Jer	
11	8518930	8518930	Gue	
11	9144119	9144119	Ang	
11	9475973	9475973	Hol	
11	9676196	9676196	Her	
11	10002642	10002642	Her	
11	10739491	10739491	Gue	
11	11532236	11620540		Guz
11	11532236	11620540		Nel
11	11540044	11540044	Bro	
11	11744765	11744765		Guz
11	11744765	11744765		Nel
11	11846607	11846607		Guz
11	11846607	11846607		Nel
11	11955169	12535496		Guz
11	11955169	12494220		Nel
11	12428643	12428643	Gue	
11	12587425	12587425	Ang	
11	13320143	13320143	Nor	
11	14522171	14675391		Guz
11	14558180	14558180	Her	

11	14587676	14675391		Nel
11	14778206	14982921		Guz
11	14778206	14982921		Nel
11	15084889	15412373		Guz
11	15084889	15084889		Nel
11	15363009	15363009		Nel
11	16865585	16865585	Jer	
11	17062464	17062464	Jer	
11	17640346	17640346	Ang	
11	17956431	17956431	Her	
11	18512028	18512028	Nor	
11	18781213	18781213	Her	
11	19598379	19598379	Ang	
11	19760018	19760018	Gue	
11	20509501	20509501	Nor	
11	21094431	21094431	Ang	
11	21094431	21094431	Hol	
11	21999151	21999151	Jer	
11	22949943	22949943	Her	
11	24356707	24356707	Nor	
11	24843332	24843332	Lim	
11	24919445	24919445	Jer	
11	25270871	25270871	Gue	
11	25411625	25411625	Jer	
11	25440847	25440847	Her	
11	27583789	27583789	Ang	
11	28506262	28506262	Jer	
11	29200605	29200605	Her	
11	29208446	29241578		Nel
11	29374448	29558210		Nel
11	29736085	29736085	Jer	
11	29806067	29806067	Gue	
11	29806067	29806067	Nor	
11	30092731	30151802	Jer	
11	31447557	31447557	Ang	
11	31468196	31468196	Bro	
11	32790675	32790675	Bro	
11	33896920	33896920	Jer	
11	34722810	34722810	Her	
11	38431802	38431802	Ang	
11	38901068	38901068	Nor	
11	39110389	39110389	Jer	
11	41315430	41315430	Ang	
11	41523105	41523105	Lim	
11	41976332	41976332	Nor	
11	42029737	42029737	Hol	
11	42029737	42029737	Jer	
11	43038137	43038137	Lim	

11	43359807	43359807	Ang	
11	43468467	43468467	Bro	
11	43755155	43755155	Jer	
11	44623029	44623029	Gue	
11	45048973	45048973	Lim	
11	45621258	45621258	Nor	
11	45870048	45870048	Her	
11	46194079	46194079	Her	
11	46400199	46400199	Hol	
11	48182216	48182216	Ang	
11	50592011	50592011	Ang	
11	50959186	50959186	Lim	
11	51769143	51769143	Jer	
11	52015128	52015128	Jer	
11	52015128	52015128	Lim	
11	52908086	53532817	Cha	
11	53473259	53473259	Lim	
11	53594492	53594492	Her	
11	53996368	53996368	Gue	
11	56158676	56158676	Hol	
11	57077011	57077011	Nor	
11	57444061	57444061	Bro	
11	58316028	58316028	Ang	
11	60423060	60423060	Bro	
11	60744028	60744028	Bro	
11	60913659	60993402	Cha	
11	61144194	61144194	Hol	
11	61950840	62507424	Cha	
11	61989432	61989432	Lim	
11	62107505	62107505	Nor	
11	62260556	62260556	Gue	
11	62566339	62566339	Ang	
11	62566339	62566339	Hol	
11	63482013	63482013	Jer	
11	63888104	63888104	Jer	
11	64098029	64098029	Lim	
11	64159759	64159759	Hol	
11	64229665	64229665	Ang	
11	65139772	65139772	Nor	
11	65444332	65444332	Bro	
11	66789796	66872690		Nel
11	66895046	66895046	Gue	
11	66930525	66930525	Hol	
11	67637286	67637286	Her	
11	68585171	68585171	Bro	
11	68616008	68616008	Lim	
11	68882864	68882864	Ang	
11	69072869	69072869	Hol	

11	69209744	69209744	Lim	
11	69565598	69565598	Lim	
11	69964661	69964661	Hol	
11	70073456	70073456		Guz
11	70133736	70133736	Her	
11	70260677	70351671	Cha	
11	70422723	70422723	Lim	
11	70547699	70547699	Nor	
11	70974393	70974393	Ang	
11	71110237	71110237	Gue	
11	71764022	71764022	Nor	
11	71857568	71857568	Ang	
11	72654390	72802766	Cha	
11	72822024	72822024	Bro	
11	73023355	73073616	Bro	
11	73023355	73588810	Cha	
11	73191754	73191754	Bro	
11	73191754	73191754	Gue	
11	73407648	73407648	Jer	
11	73453361	73453361	Bro	
11	73520084	73520084	Lim	
11	74083671	74083671	Hol	
11	74215819	74215819	Cha	
11	74215819	74215819	Lim	
11	74350377	74599988	Cha	
11	74541961	74541961	Gue	
11	74731727	74793726	Cha	
11	74924368	74924368	Cha	
11	75037477	75387992	Cha	
11	75088356	75088356	Bro	
11	75324266	75324266	Gue	
11	75896887	75896887	Hol	
11	75972707	75972707	Nor	
11	76679431	76679431	Gue	
11	77488961	77488961	Lim	
11	77719309	77719309	Her	
11	78293151	78293151	Lim	
11	78559406	78559406	Hol	
11	79457949	79457949	Ang	
11	80730546	80730546	Her	
11	83210249	83210249	Bro	
11	83426113	83426113	Hol	
11	83702805	83702805	Bro	
11	83866684	83866684	Lim	
11	84229041	84229041	Ang	
11	85789132	85789132	Ang	
11	85997549	85997549	Her	
11	86030202	86030202	Ang	

11	86685748	86685748	Her
11	86953667	86953667	Bro
11	87137735	87137735	Bro
11	88056164	88056164	Bro
11	88379504	88379504	Jer
11	89454963	89454963	Gue
11	90475740	90475740	Jer
11	92530892	92530892	Gue
11	94181367	94181367	Jer
11	94590620	94590620	Hol
11	94590620	94590620	Nor
11	95212298	95212298	Ang
11	95802430	95802430	Hol
11	97189737	97189737	Bro
11	99096532	99096532	Lim
11	99985406	99985406	Lim
11	100742447	100742447	Ang
11	101262682	101262682	Gue
11	101759879	101841579	Gue
11	102227232	102227232	Nor
11	102501787	102501787	Nor
11	102883420	102883420	Bro
11	103410170	103410170	Jer
11	104117370	104117370	Bro
11	106402446	106402446	Ang
11	106402446	106402446	Jer
12	2203849	2203849	Her
12	2411488	2411488	Bro
12	2610868	2610868	Jer
12	4026108	4026108	Her
12	4195607	4195607	Gue
12	4329618	4329618	Her
12	4807493	4807493	Lim
12	5074528	5074528	Ang
12	5717994	5717994	Lim
12	6880711	6880711	Lim
12	9047249	9047249	Bro
12	10384095	10384095	Bro
12	10566940	10566940	Nor
12	10640416	10640416	Gue
12	10858590	10858590	Her
12	12353385	12353385	Bro
12	12756105	12756105	Nor
12	13111153	13111153	Jer
12	14985891	14985891	Her
12	15637232	15637232	Ang
12	16030260	16030260	Hol
12	16187847	16187847	Her

12	16790859	16790859		Jer
12	17044377	17044377		Nor
12	17407295	17407295		Bro
12	18003452	18003452		Jer
12	18106772	18106772		Gue
12	18132138	18132138		Her
12	19917196	19917196		Bro
12	20841058	20841058		Her
12	22840328	22840328		Ang
12	24151669	24151669		Nor
12	24776838	24776838		Lim
12	25448771	25448771		Jer
12	25941332	25941332		Nor
12	26891864	26891864		Hol
12	27114388	27114388		Bro
12	27344275	27344275		Ang
12	27935604	29508940	ZT	
12	28137445	28137445		Hol
12	28514178	28514178		Gue
12	30766995	30766995		Nor
12	31157450	31157450		Nor
12	31787030	31787030		Nor
12	32438446	32438446		Jer
12	32596654	32596654		Her
12	33312158	33312158		Ang
12	34894964	34944506		Lim
12	35250806	35250806		Nor
12	37966212	37966212		Nor
12	38858520	38858520		Nor
12	42222361	42222361		Bro
12	42601919	42601919		Her
12	42601919	42601919		Hol
12	43242579	43242579		Hol
12	43242579	43242579		Jer
12	43575958	43575958		Lim
12	44120862	44120862		Gue
12	46269299	46269299		Bro
12	47603732	47603732		Lim
12	48557678	48557678		Lim
12	48587202	48587202		Ang
12	48831255	48831255		Hol
12	49756074	49756074		Hol
12	50223292	50223292		Gue
12	50813072	50813072		Ang
12	50859036	50859036		Jer
12	52147705	52147705		Gue
12	52647465	52647465		Her
12	53070951	53070951		Bro

12	53213890	53213890	Her	
12	53777939	53777939	Ang	
12	54241569	54241569	Gue	
12	54754795	54754795	Jer	
12	55414014	55414014	Bro	
12	55903047	55903047	Ang	
12	56419641	56419641	Hol	
12	56782756	56782756	Bro	
12	56938705	56938705	Her	
12	58710498	58710498	Ang	
12	58997484	58997484	Ang	
12	61101274	61101274	Gue	
12	61676514	61676514	Jer	
12	62285245	62285245	Lim	
12	62996126	62996126	Hol	
12	63259793	63259793	Ang	
12	66684733	66684733	Hol	
12	67104828	67104828	Ang	
12	68509420	68509420	Jer	
12	68708862	68708862	Lim	
12	69067361	69067361	Her	
12	70116727	70116727	Her	
12	78810215	78810215	Nor	
12	79257925	79257925	Her	
12	79377538	79377538	Hol	
12	82625429	82625429	Lim	
12	82849097	82849097	Bro	
12	85076524	85076524	Lim	
12	85134208	85134208	Her	
12	85516869	85516869	Lim	
12	85698140	85698140	Ang	
12	85862176	85862176	Lim	
12	86086671	86086671	Lim	
12	86840773	86840773	Lim	
12	87070903	87070903	Nor	
12	87211312	87211312	Gue	
12	89066393	89263967		Guz
12	89122653	89557545		Gir
12	89355616	89664275	Cha	
12	89664275	89664275	Lim	
12	90774139	90774139	Her	
12	91048415	91048415	Ang	
12	91091598	91091598	Nor	
13	534257	534257	Bro	
13	802694	802694	Bro	
13	1393404	1393404	Hol	
13	1676337	1676337	Gue	
13	2954757	2954757	Ang	

13	3919075	3919075	Ang	
13	6343393	6343393	Jer	
13	6402417	6402417	Hol	
13	7605879	7605879	Ang	
13	9229380	9229380	Bro	
13	9374861	9374861	Gue	
13	9585434	9585434	Gue	
13	9764993	9764993	Jer	
13	10356935	10356935	Bro	
13	10564434	10564434	Ang	
13	11630764	12073488	Cha	
13	11763891	11794896	Ang	
13	11862167	11862167	Lim	
13	12017225	12017225	Lim	
13	12626523	12626523	Her	
13	13210821	13226766	Cha	
13	14020222	14020222	Her	
13	15665548	15665548	Ang	
13	16298764	16298764	Gue	
13	17335139	17335139	Nor	
13	18905143	18905143	Ang	
13	19547365	19547365	Her	
13	19696845	19696845	Nor	
13	20215298	20215298	Ang	
13	21167068	21167068	Jer	
13	21453849	21453849	Hol	
13	21649552	21649552	Bro	
13	21989172	21989172	Jer	
13	22470648	22470648	Her	
13	22611652	22611652	Her	
13	23767720	23767720	Nor	
13	24218141	24218141	Hol	
13	24218141	24218141	Lim	
13	25229684	25418630		Guz
13	25349272	25349272	Her	
13	25632784	25632784	Ang	
13	26871148	26871148	Hol	
13	26937127	26937127	Bro	
13	26974732	26974732	Lim	
13	27251090	27251090	Bro	
13	27365455	27365455	Lim	
13	28041352	28041352	Gue	
13	28901873	28901873	Lim	
13	29022697	29022697	Lim	
13	30052358	30052358	Gue	
13	30528915	30528915	Lim	
13	31110305	31110305	Bro	
13	31212984	31212984	Bro	

13	31264582	31264582		Hol	
13	31582538	32107494			Guz
13	31691092	31691092		Lim	
13	31861758	31861758		Nor	
13	31987072	31987072		Jer	
13	32251293	32251293			Guz
13	32368052	32368052			Guz
13	32793714	32793714		Lim	
13	33015023	33015023		Hol	
13	33184186	33184186		Nor	
13	33646254	33646254		Jer	
13	33881397	33881397		Jer	
13	34080843	34080843		Lim	
13	34119211	34234546	ZT		
13	34336406	34554671	ZT		
13	34659293	35054048	ZT		
13	35841589	35841589		Ang	
13	36614926	36614926		Lim	
13	37710271	37710271		Her	
13	37836399	37836399		Ang	
13	37990777	37990777		Jer	
13	38971698	39215562			Nel
13	41141645	41141645		Ang	
13	42015567	42151317			Nel
13	42236684	42364607			Gir
13	44187504	44187504		Nor	
13	45253509	45533567			Gir
13	45465713	45465713		Her	
13	46174667	46174667		Nor	
13	47103298	47103298		Jer	
13	48893096	48893096	ZT		
13	49031363	49197290	ZT		
13	49300389	49816619	ZT		
13	49628146	49847477			Guz
13	49963611	50055754			Guz
13	50231261	50291741			Guz
13	50291741	50291741			Nel
13	50451529	50451529			Guz
13	50451529	50451529			Nel
13	50565794	50644646			Guz
13	50565794	50644646			Nel
13	50692816	50692816		Jer	
13	50760200	50760200			Guz
13	50760200	50760200			Nel
13	50869402	51095524			Guz
13	50869402	51095524			Nel
13	51214983	51489943			Guz
13	51397521	51397521		Bro	

13	51604518	51604518	Nor	
13	51616130	51616130		Guz
13	51755553	51929601		Guz
13	52030693	52086828		Guz
13	52215695	52215695		Guz
13	52309239	52309239	Bro	
13	52352327	52352327		Guz
13	53370379	53370379	Bro	
13	54019491	54019491	Her	
13	54136766	54178604	Bro	
13	54235658	54235658	Her	
13	54416245	54416245	Her	
13	54416245	54416245	Hol	
13	54679600	54679600	Jer	
13	56083986	56083986	Bro	
13	58880236	58928119	Ang	
13	60912509	60912509	Bro	
13	61207085	61207085	Her	
13	63023671	63023671	Jer	
13	63287690	63287690	Ang	
13	66357502	66357502	Her	
13	67817498	67817498	Nor	
13	68228050	68228050	Gue	
13	68396195	68396195	Nor	
13	69638087	69638087	Jer	
13	70886673	70886673	Jer	
13	71499910	71499910	Nor	
13	71833920	71833920	Hol	
13	72491288	72491288	Ang	
13	73180097	73180097	Jer	
13	74480533	74480533	Jer	
13	75187129	75187129	Bro	
13	75436349	75672837		Nel
13	76822779	76822779	Jer	
13	77346284	77346284	Her	
13	77884465	77944238	Bro	
13	79586048	79586048	Ang	
13	79586048	79586048	Hol	
13	80021463	80021463	Lim	
13	80536435	80536435	Hol	
13	82124568	82124568	Lim	
13	82240934	82240934	Lim	
13	82913657	82913657	Bro	
13	83500584	83500584	Gue	
14	593705	593705	Nor	
14	2580667	2580667	Hol	
14	3734807	3734807	Nor	
14	3852717	3852717	Ang	

14	3942818	3942818		Hol	
14	4716252	4716252		Jer	
14	5095604	5095604		Her	
14	5095604	5095604		Hol	
14	5118510	5118510		Gue	
14	5225467	5225467		Nor	
14	5396141	5396141		Nor	
14	5474762	5474762		Lim	
14	6620615	6620615		Ang	
14	6883461	6883461		Gue	
14	8297461	8297461		Jer	
14	8931824	8931824		Hol	
14	9449027	9449027		Nor	
14	9760053	9760053		Jer	
14	10164698	10164698		Ang	
14	10164698	10164698		Nor	
14	10240454	10240454		Her	
14	10941384	10941384		Gue	
14	11501300	11501300		Gue	
14	11554484	11554484		Hol	
14	13320866	13320866		Her	
14	13594566	13594566		Nor	
14	16786915	16786915		Nor	
14	16957863	16957863		Her	
14	17760635	17760635		Ang	
14	19278453	19278453		Jer	
14	19426465	19426465		Ang	
14	19524665	19524665		Bro	
14	20269546	20269546		Ang	
14	20595450	20595450		Jer	
14	21725632	21725632		Ang	
14	22633666	22633666		Lim	
14	23477783	23477783		Gue	
14	23922664	23922664		Bro	
14	24603090	25298972	ZT		
14	25015640	25015640		Nor	
14	26126660	26126660		Her	
14	28399107	28399107		Nor	
14	28616985	28616985		Bro	
14	28822547	28822547		Hol	
14	30557994	30557994		Her	
14	31497667	31497667		Jer	
14	32242397	32242397		Bro	
14	32961490	33028131			Guz
14	35419556	35419556		Bro	
14	36487168	36487168		Hol	
14	36715710	37511658	ZT		
14	36829769	36829769		Gue	

14	38919669	39027008	ZT		
14	39024260	39024260		Gue	
14	39024260	39024260		Her	
14	39523938	39523938		Jer	
14	40889968	40889968		Gue	
14	42121450	42376970	ZT		
14	43460382	43460382		Lim	
14	43811139	43811139		Lim	
14	44169293	44169293		Gue	
14	44269436	44269436		Bro	
14	44979200	44979200		Ang	
14	45478315	46437276	ZT		
14	46456216	46562573		Cha	
14	46868870	46868870		Her	
14	47201844	47201844		Hol	
14	47780752	47780752		Ang	
14	48337384	48337384		Nor	
14	50191506	50191506		Jer	
14	50502349	50502349		Nor	
14	51084182	51084182		Lim	
14	52415429	52415429		Gue	
14	53193111	53193111		Her	
14	53726449	53726449		Lim	
14	54164119	54164119		Her	
14	54402173	54402173		Hol	
14	54636577	54636577		Gue	
14	55872388	55872388		Bro	
14	58831995	58831995		Gue	
14	59086394	59086394		Her	
14	59145420	59145420			Guz
14	59699691	59699691		Bro	
14	59956308	59956308		Ang	
14	61386112	61499185			Guz
14	61728798	61728798		Jer	
14	62155894	62155894		Her	
14	63471687	63471687		Ang	
14	63971497	63971497		Hol	
14	64525338	64525338		Hol	
14	64892207	64892207		Jer	
14	64921543	64921543		Gue	
14	65425379	65425379		Ang	
14	65835855	65835855		Hol	
14	66153764	66153764		Nor	
14	66549938	66549938		Ang	
14	66610254	66610254		Lim	
14	66786047	66924336			Guz
14	67314952	67314952		Jer	
14	67977853	67977853		Bro	

14	68620692	68847775	Cha	
14	68705641	68705641	Hol	
14	68705641	68705641	Jer	
14	68924060	68924060	Ang	
14	69802514	69802514	Nor	
14	73531668	73531668	Gue	
14	73755259	73755259	Nor	
14	74310747	74310747	Nor	
14	74482108	74482108	Ang	
14	77810155	77897196	Nor	
14	78081859	78081859	Bro	
14	78558407	78558407	Gue	
14	78764654	78764654	Nor	
14	80468495	80468495	Bro	
14	80838143	80838143	Gue	
14	81238462	81238462	Hol	
14	81238462	81238462	Jer	
14	81449754	81449754	Gue	
14	82299086	82299086	Jer	
14	82510580	82510580	Hol	
14	82873341	82873341	Hol	
14	83085249	83200348	Cha	
14	83124356	83124356	Bro	
14	83385461	83385461	Jer	
14	83666534	83831390		Gir
14	83776145	83776145	Ang	
14	83975039	83975039	Hol	
14	84193809	84193809	Her	
14	84378204	84378204	Ang	
15	577326	577326	Her	
15	1533401	1533401	Her	
15	4518219	4518219	Gue	
15	4749879	4749879	Jer	
15	6063913	6063913	Her	
15	6335201	6335201	Jer	
15	6883177	6883177	Her	
15	8803547	8803547	Jer	
15	8923661	8923661	Ang	
15	8963352	8963352	Lim	
15	9168098	9168098	Lim	
15	9959794	9959794	Lim	
15	10479750	10479750	Ang	
15	11505690	11505690	Lim	
15	14452509	14452509	Bro	
15	14452509	14452509	Lim	
15	15554569	15554569	Nor	
15	17527385	17527385	Her	
15	18397101	18397101	Gue	

15	19622027	19622027	Her	
15	19813387	19813387	Gue	
15	20544975	20544975	Jer	
15	20702646	20702646	Lim	
15	20748077	20748077	Her	
15	24107618	24107618	Ang	
15	25191633	25191633	Gue	
15	26219411	26219411	Bro	
15	27461860	27461860	Gue	
15	28187349	28187349	Gue	
15	29099098	29099098	Her	
15	29427085	29427085	Hol	
15	30219463	30219463	Jer	
15	30794937	30794937	Nor	
15	31146406	31146406	Lim	
15	31593742	31671928	Gue	
15	31745902	31745902	Jer	
15	32060613	32060613	Lim	
15	32474091	32474091	Gue	
15	33781326	33781326	Lim	
15	34174802	34174802	Nor	
15	34791323	34791323	Ang	
15	35293898	35293898	Her	
15	35442019	35442019	Her	
15	35577401	35577401	Ang	
15	37735270	37735270	Gue	
15	37920515	37920515	Hol	
15	37953561	37953561	Lim	
15	38488528	38488528	Bro	
15	38787942	38787942	Gue	
15	39384673	39384673	Lim	
15	40192007	40192007	Jer	
15	40316847	40316847	Gue	
15	40450742	40450742	Her	
15	41157757	41157757	Ang	
15	41609973	41609973	Jer	
15	41832209	41832209	Bro	
15	42021233	42370575		Nel
15	42155423	42370575		Guz
15	42478754	42559771		Nel
15	42591956	42591956	Hol	
15	44726542	44726542	Bro	
15	44939719	44939719	Hol	
15	46005939	46005939	Gue	
15	47017013	47017013	Her	
15	47605562	47605562	Her	
15	47797093	47797093	Lim	
15	49571663	49571663	Hol	

15	51548685	51548685	Jer	
15	52323484	52323484	Her	
15	52420655	52420655	Lim	
15	52869669	52869669	Nor	
15	54216680	54216680	Her	
15	55884002	55884002	Lim	
15	56445798	56445798	Nor	
15	56820572	56820572	Lim	
15	57243853	57243853	Nor	
15	57287768	57287768	Gue	
15	57599024	57599024	Nor	
15	58497739	58497739	Lim	
15	58746126	58746126	Hol	
15	59308913	59308913	Jer	
15	59620713	59620713	Jer	
15	61230076	61230076	Her	
15	61636045	61636045	Lim	
15	61906679	61906679	Jer	
15	63453218	63453218	Nor	
15	64571517	64571517	Jer	
15	65142614	65142614	Bro	
15	65804969	65804969	Ang	
15	66064827	66133731	Hol	
15	66309896	66309896	Lim	
15	66853433	67024310		Gir
15	67679022	67679022	Jer	
15	69941897	69941897	Her	
15	70174702	70174702	Her	
15	70990116	70990116	Nor	
15	71307101	71307101	Nor	
15	72387341	72387341	Her	
15	72706627	72738024		Nel
15	72760977	72760977	Hol	
15	73905238	73905238	Gue	
15	73984029	73984029	Ang	
15	73984029	73984029	Jer	
15	74290188	74290188		Nel
15	74423905	74524111		Nel
15	74501976	74501976	Her	
15	74955442	74955442	Lim	
15	75376248	75376248	Hol	
15	75749702	75749702	Jer	
15	76425084	76425084	Ang	
15	76425084	76425084	Nor	
15	77456598	77456598	Jer	
15	78305649	78305649	Bro	
15	78770047	78770047	Bro	
15	78896843	78896843	Gue	

15	81230124	81230124	Jer	
15	81583592	81583592	Bro	
15	82180549	82180549	Gue	
15	83664314	83664314	Ang	
15	83952725	83974893	Ang	
15	85085535	85085535	Ang	
16	950232	950232	Ang	
16	950232	950232	Her	
16	1951374	1951374	Nor	
16	3513917	3513917	Ang	
16	4154922	4154922	Hol	
16	4294296	4340374	Gue	
16	4544485	4544485	Nor	
16	9474801	9474801	Ang	
16	9929930	9929930	Jer	
16	12902221	12902221	Ang	
16	13021637	13021637	Her	
16	14329324	14405522	Nor	
16	15297195	15297195	Her	
16	15416252	15416252	Jer	
16	15655956	15655956	Bro	
16	15771063	15771063	Jer	
16	16030130	16030130	Lim	
16	16136785	16136785	Lim	
16	16398298	16398298	Hol	
16	17508199	17508199	Her	
16	17974220	17974220	Nor	
16	18263233	18263233	Hol	
16	18818921	18818921	Her	
16	19528648	19528648	Jer	
16	19691718	19691718	Nor	
16	20356811	20356811	Gue	
16	20629654	20629654	Gue	
16	20721140	20721140	Her	
16	20780071	20780071	Lim	
16	21058625	21058625	Lim	
16	22165889	22165889	Lim	
16	22743482	22743482	Her	
16	23194508	23194508	Lim	
16	23435304	23435304	Her	
16	24415438	24984384	Cha	
16	24503655	24503655	Lim	
16	24682103	24856863		Nel
16	24710520	24845497	Bro	
16	24710520	24845497	Gue	
16	24740093	24740093	Ang	
16	25407599	25407599	Ang	
16	25407599	25407599	Gue	

16	25407599	25407599		Hol	
16	26080421	26080421		Jer	
16	26300274	26300274		Ang	
16	26599441	26599441		Ang	
16	26992388	26992388		Hol	
16	27360346	27360346		Hol	
16	27592472	27592472		Her	
16	27618028	27618028		Nor	
16	27857164	27857164		Nor	
16	28785286	28960102			Gir
16	29189081	29189081		Nor	
16	30143682	30143682		Nor	
16	30228692	30228692		Gue	
16	30551740	30551740		Her	
16	30618697	30618697		Ang	
16	30991351	30991351		Nor	
16	31793568	31793568		Hol	
16	31793568	31793568		Jer	
16	31929371	31929371		Ang	
16	33496176	33496176		Ang	
16	33496176	33496176		Hol	
16	33496176	33496176		Jer	
16	35128071	35128071		Lim	
16	36397735	36397735		Nor	
16	36882994	36882994		Hol	
16	36882994	36882994		Nor	
16	38757722	38757722		Her	
16	39086170	39086170		Hol	
16	40044051	40309659			Gir
16	40318965	40545245	ZT		
16	40656961	40656961	ZT		
16	40886797	41149860	ZT		
16	41453104	41453104		Lim	
16	41564542	41564542	ZT		
16	41672654	41767607	ZT		
16	41775870	41775870		Jer	
16	41877813	42407997	ZT		
16	42667035	42667035		Nor	
16	43250880	43313085	ZT		
16	43419087	43501100	ZT		
16	44277286	45534177	ZT		
16	44584439	44584439		Hol	
16	44879718	44879718		Nor	
16	45232127	45919794			Guz
16	45433371	45773321			Nel
16	46038878	46038878			Guz
16	46970643	46970643		Ang	
16	47442862	47442862		Jer	

16	47894912	47894912	Ang	
16	47894912	47894912	Nor	
16	47974232	47974232	Bro	
16	48326002	48326002	Her	
16	48504078	48504078	Hol	
16	48504078	48504078	Nor	
16	48836104	48836104	Gue	
16	49045020	49045020		Guz
16	49590524	49590524	Hol	
16	50501339	50501339	Jer	
16	51661530	51661530	Jer	
16	52389421	52389421	Ang	
16	53741575	53741575	Lim	
16	53783614	53783614	Nor	
16	53826914	53826914	Bro	
16	56867692	56867692	Hol	
16	57858883	57858883	Bro	
16	58022121	58022121	Ang	
16	58895036	59521908		Guz
16	58938382	58938382	Lim	
16	59033013	59345789		Nel
16	59301385	59301385	Nor	
16	60023796	60023796	Hol	
16	60497162	60540206		Nel
16	61056572	61056572	Nor	
16	61686279	61686279	Lim	
16	61728924	61757564	Her	
16	62564542	62564542	Ang	
16	62564542	62661510	Gue	
16	62564542	62564542	Nor	
16	62935327	62935327	Bro	
16	64704446	65339532		Nel
16	65313199	65313199	Hol	
16	68076567	68076567	Bro	
16	68525706	68525706	Nor	
16	68663695	69853720		Guz
16	68730323	68920589		Nel
16	68935940	68935940	Lim	
16	69010341	69010341	Bro	
16	69061411	69853720		Nel
16	69205352	69808147		Gir
16	69263110	69263110	Hol	
16	71035322	71035322	Ang	
16	71562994	71562994	Her	
16	74300380	74300380	Lim	
16	74656398	74656398	Lim	
16	74784360	74784360	Her	
16	75455078	75455078	Hol	

16	75580704	75580704	Hol	
16	76531079	76531079	Ang	
16	77398493	77398493	Jer	
16	77571128	77571128	Gue	
16	77678176	77678176	Nor	
16	78102411	78102411	Ang	
16	78174119	78596042		Guz
16	78257225	78257225	Hol	
16	79746720	79746720	Gue	
16	80114473	80114473	Ang	
16	81672961	81672961	Jer	
17	852669	852669	Jer	
17	1781813	1781813	Hol	
17	2321511	2321511	Gue	
17	2607518	2607518	Hol	
17	3149962	3149962	Hol	
17	4791944	4791944	Jer	
17	4834929	4834929	Her	
17	5549379	5549379	Hol	
17	6662551	6662551	Lim	
17	7658075	7658075	Gue	
17	7765177	7765177	Gue	
17	8067629	8067629	Her	
17	8335532	8335532	Her	
17	8410348	8410348	Ang	
17	8410348	8410348	Nor	
17	9912468	9912468	Bro	
17	9981281	9981281	Gue	
17	10319304	10319304	Gue	
17	10335313	10335313	Her	
17	10967262	10967262	Hol	
17	11541627	11541627	Bro	
17	11667404	11667404	Jer	
17	12245185	12245185	Ang	
17	12678479	12678479	Ang	
17	12713251	12713251	Lim	
17	12857924	12857924	Gue	
17	14191364	14191364	Hol	
17	16585610	16585610	Hol	
17	16668439	16668439	Her	
17	16712824	16712824	Ang	
17	17126550	17126550	Ang	
17	18216663	18216663	Lim	
17	18335608	18335608	Nor	
17	19236474	19236474	Hol	
17	19771813	19771813	Hol	
17	20462863	20462863	Bro	
17	21280317	21280317	Gue	

17	21627514	21627514	Her
17	22012082	22012082	Hol
17	22384870	22384870	Her
17	23219327	23219327	Bro
17	23440483	23440483	Hol
17	23686714	23686714	Nor
17	24774230	24774230	Jer
17	27057425	27057425	Her
17	28502802	28502802	Gue
17	32589781	32589781	Lim
17	34443645	34443645	Ang
17	34620959	34620959	Hol
17	34675555	34953139	Cha
17	34953139	34953139	Hol
17	36502591	36502591	Gue
17	36502591	36502591	Her
17	36571163	36935056	Cha
17	36602912	36602912	Lim
17	36778666	36778666	Lim
17	36935056	36935056	Her
17	37300822	37300822	Ang
17	38832181	38832181	Bro
17	38832181	38832181	Gue
17	39288058	39288058	Hol
17	39347048	39347048	Jer
17	39933636	39933636	Gue
17	40004303	40289698	Cha
17	40192981	40226461	Ang
17	40478595	40535983	Hol
17	40478595	40478595	Jer
17	40597804	40597804	Lim
17	41526248	41526248	Ang
17	41701959	41701959	Jer
17	43711813	43711813	Her
17	44303353	44303353	Jer
17	44303353	44303353	Lim
17	44694694	44694694	Jer
17	47123292	47123292	Bro
17	47123292	47123292	Gue
17	47335871	47335871	Bro
17	47876580	47876580	Hol
17	48049218	48049218	Lim
17	48737633	48737633	Bro
17	48800637	48800637	Jer
17	48846124	48904473	Her
17	49183026	49183026	Jer
17	52267308	52267308	Gue
17	52526310	52526310	Ang

17	52604653	52604653	Lim	
17	52768653	52768653	Lim	
17	52811292	52811292	Gue	
17	52998036	52998036	Gue	
17	53452610	53452610	Ang	
17	53452610	53894855	Cha	
17	53452610	53452610	Hol	
17	53562854	53562854	Lim	
17	53719313	53719313	Bro	
17	53966240	53966240	Ang	
17	54425265	54425265	Lim	
17	54495107	54495107	Her	
17	54738535	54738535	Hol	
17	54800208	55184003	Cha	
17	54938787	54938787	Bro	
17	55247896	55247896	Gue	
17	55646327	55646327	Gue	
17	56264668	56264668	Gue	
17	56390012	56390012	Ang	
17	56488289	56488289	Hol	
17	56826610	56915577		Nel
17	56975285	56975285	Jer	
17	57289572	57289572	Jer	
17	58025358	58025358	Bro	
17	58419025	58419025	Hol	
17	58536079	58536079	Hol	
17	59570096	59570096		Gir
17	61012828	61012828	Jer	
17	61745667	61745667	Gue	
17	61745667	61745667	Jer	
17	62091890	62091890	Lim	
17	62437757	62437757	Lim	
17	62883633	62883633	Lim	
17	63080286	63080286	Gue	
17	63185013	63356608	Cha	
17	63315449	63315449	Bro	
17	63593490	63593490	Gue	
17	63765280	63765280	Hol	
17	64337986	64337986	Hol	
17	65431771	65431771	Hol	
17	65724202	65724202	Gue	
17	65795029	65795029	Her	
17	65985836	65985836	Gue	
17	66404458	66404458	Jer	
17	66807833	66807833	Gue	
17	66879214	66879214	Ang	
17	67975896	68078441		Nel
17	68579649	68579649	Lim	

17	68612895	68612895		Gue	
17	68642664	68642664		Nor	
17	68882202	68882202		Her	
17	69359168	70008031			Guz
17	69496828	69727937			Nel
17	70166943	70166943			Guz
17	70797006	70797006		Bro	
17	70913770	70913770		Lim	
17	71139021	71139021		Gue	
17	71675771	71675771		Hol	
17	72189054	72189054		Ang	
17	72189054	72189054		Gue	
17	73083885	73083885		Lim	
17	73224194	73224194		Nor	
17	73878627	73878627		Nor	
17	74235267	74292319		Ang	
17	74235267	74235267		Nor	
17	74412830	74412830		Her	
18	514332	514332		Her	
18	806069	806069		Jer	
18	959986	959986		Lim	
18	1653679	1653679		Jer	
18	3349402	3349402		Jer	
18	3835280	3835280		Ang	
18	4541123	4541123		Jer	
18	5279153	5279153		Bro	
18	5319149	5440260		Cha	
18	5417593	5417593		Bro	
18	5514066	5514066		Ang	
18	5606378	5606378		Lim	
18	6053445	6053445		Her	
18	6311708	6311708		Bro	
18	6792097	6792097		Jer	
18	8207165	8349174		Cha	
18	8267599	8267599		Hol	
18	8310093	8310093		Bro	
18	8310093	8310093		Gue	
18	8461263	8461263		Hol	
18	9101606	9101606		Hol	
18	9449975	9449975		Hol	
18	9662984	9662984		Gue	
18	9662984	9662984		Jer	
18	10215813	10215813		Hol	
18	10507911	10507911		Bro	
18	10618754	10618754		Bro	
18	11061389	11061389		Her	
18	11147240	11147240		Hol	
18	11298096	11372486	ZT		

18	11484200	11959392	ZT		
18	11605447	11605447		Bro	
18	14171624	14702657	ZT		
18	15167441	15167441		Ang	
18	15167441	15167441		Hol	
18	15659112	15659112		Her	
18	15659112	15659112		Hol	
18	15739507	16095680			Gir
18	16199824	16418196			Gir
18	17269742	17269742		Bro	
18	17272777	17548027			Nel
18	17560586	17560586		Lim	
18	18424292	18424292		Jer	
18	19492608	19492608		Gue	
18	19738288	19738288		Her	
18	23118542	23118542		Lim	
18	23347562	23347562		Lim	
18	24531834	24531834		Her	
18	25073940	25073940		Gue	
18	25804502	25804502		Jer	
18	26289657	26289657		Hol	
18	28641862	28703331			Nel
18	28852172	28939981			Nel
18	29073309	29120448			Nel
18	29223607	29223607		Ang	
18	30500066	30500066		Her	
18	30705918	30705918		Hol	
18	31614885	31614885		Her	
18	31661075	31661075		Gue	
18	32416748	32416748		Jer	
18	33293455	33293455		Bro	
18	33395535	33395535		Her	
18	33622799	33622799		Ang	
18	33992332	33992332		Bro	
18	34030879	34030879		Jer	
18	36674600	36674600		Nor	
18	37545832	37545832		Nor	
18	37573172	37573172		Ang	
18	37573172	37573172		Hol	
18	38323757	38323757		Lim	
18	39780582	39780582		Hol	
18	40864318	40864318		Lim	
18	41280825	41280825		Hol	
18	43518866	43518866		Ang	
18	43975857	43975857		Bro	
18	45402866	45402866		Gue	
18	46033080	46033080		Her	
18	47130199	47130199		Gue	

18	47311006	47311006	Lim
18	47909267	47909267	Bro
18	48314149	48314149	Gue
18	50305250	50305250	Her
18	51153494	51153494	Gue
18	52370981	52370981	Bro
18	52609336	52609336	Her
18	54276361	54276361	Ang
18	54852067	54852067	Nor
18	55819899	55819899	Bro
18	58047616	58047616	Ang
18	58459210	58459210	Hol
18	59835694	59835694	Bro
18	60467996	60467996	Hol
18	60467996	60467996	Nor
18	62253639	62253639	Jer
18	62695412	62695412	Hol
18	63020277	63020277	Ang
18	63457199	63457199	Bro
18	64137120	64137120	Bro
18	65097218	65097218	Hol
19	2609059	2609059	Bro
19	2609059	2609059	Gue
19	2924545	2924545	Hol
19	3216655	3216655	Nor
19	3710275	3710275	Nor
19	4268920	4268920	Gue
19	5622026	5622026	Ang
19	5806258	5806258	Ang
19	7145391	7145391	Her
19	8802707	8802707	Bro
19	9621306	9621306	Nor
19	9739269	9739269	Hol
19	10671018	10671018	Bro
19	11188424	11188424	Her
19	11535729	11535729	Ang
19	11535729	11535729	Hol
19	11535729	11535729	Jer
19	12063906	12201723	Cha
19	12201723	12201723	Lim
19	12251625	12251625	Bro
19	12955639	12955639	Her
19	13423217	13423217	Bro
19	13840644	13840644	Her
19	14274648	14274648	Ang
19	14525387	14525387	Gue
19	15293355	15293355	Bro
19	15442296	15442296	Ang

19	15442296	15442296	Jer
19	16180128	16180128	Bro
19	17208514	17208514	Her
19	17693331	17693331	Bro
19	17910717	17910717	Lim
19	18156962	18156962	Hol
19	18380038	18380038	Gue
19	18419654	18419654	Hol
19	19034517	19034517	Her
19	19577234	19577234	Gue
19	20878990	20878990	Jer
19	20945594	20945594	Gue
19	21758532	21758532	Hol
19	22386428	22386428	Lim
19	22540340	22540340	Lim
19	22587535	22587535	Bro
19	22623108	22623108	Gue
19	23038861	23038861	Lim
19	23211385	23529957	Cha
19	23253079	23253079	Lim
19	23400125	23400125	Ang
19	23465768	23494003	Gue
19	23494003	23529957	Bro
19	23511125	23511125	Nor
19	23621511	23621511	Gue
19	23650335	23650335	Lim
19	25304574	25304574	Bro
19	25669872	25669872	Her
19	26087727	26087727	Her
19	27340012	27340012	Her
19	28644586	28644586	Hol
19	29715194	29715194	Ang
19	29715194	29715194	Nor
19	29970036	29970036	Hol
19	30291199	30291199	Gue
19	30291199	30291199	Her
19	31400400	31400400	Ang
19	33103125	33103125	Her
19	33633705	33633705	Ang
19	34533313	34533313	Gue
19	36727804	36727804	Nor
19	37041794	37041794	Gue
19	37693334	37693334	Bro
19	38089892	38089892	Nor
19	38474828	38474828	Ang
19	41584389	41584389	Ang
19	41584389	41665727	Nor
19	42757515	42757515	Jer

19	46370360	46370360		Lim
19	46477611	46477611		Hol
19	46580893	46580893		Gue
19	46696973	46696973		Her
19	47473217	47473217		Hol
19	48607730	48607730		Bro
19	49080697	49149854		Lim
19	49445681	49445681		Gue
19	49704150	49704150		Gue
19	50087148	50087148		Gue
19	50164188	50218827		Lim
19	51718265	51718265		Lim
19	53871632	53871632		Her
19	54191766	54191766		Jer
19	55330510	55330510		Jer
19	55460966	55460966		Hol
19	55511768	55511768		Lim
19	56080387	56080387		Bro
19	57805558	57805558		Hol
19	59282431	59282431		Ang
19	59718254	59718254		Hol
19	60271884	60271884		Bro
19	61021458	61021458		Gue
19	61021458	61021458		Nor
19	61160708	61160708		Hol
19	62726258	62726258		Jer
19	62956338	62956338		Her
19	63511364	63511364		Gue
19	63511364	63511364		Nor
20	220247	220247		Nor
20	276942	276942		Bro
20	434778	434778		Jer
20	1321610	1321610		Her
20	1798415	1798415		Her
20	1868761	1868761		Jer
20	1892651	1892651		Lim
20	6991289	6991289		Lim
20	8327825	8327825		Ang
20	9123270	9123270		Ang
20	9504319	9504319		Gue
20	10113604	10113604		Ang
20	10520408	10520408		Ang
20	10762327	10762327		Ang
20	11177346	11177346		Her
20	13714109	14014051	ZT	
20	14121548	14441958	ZT	
20	14563004	14563004	ZT	
20	14708423	14708423	ZT	

20	14822650	15011780	ZT		
20	15135107	15135107	ZT		
20	15329597	15329597		Ang	
20	17454072	17454072		Hol	
20	20545621	20545621		Gue	
20	21661324	21661324		Bro	
20	21896629	21896629		Ang	
20	22150287	22150287		Jer	
20	23017834	23017834		Nor	
20	25779683	25779683		Bro	
20	26335111	26335111		Nor	
20	26707247	26707247		Her	
20	27748175	27748175		Her	
20	27803244	27909789			Nel
20	28546796	28546796		Her	
20	28836898	28836898		Ang	
20	29419333	29419333		Her	
20	29714923	29714923		Nor	
20	31615010	31834900			Guz
20	31679503	31894246			Nel
20	32851502	32851502		Nor	
20	33048635	33048635		Lim	
20	33327920	33327920		Ang	
20	33702432	33702432		Lim	
20	34637707	34637707		Ang	
20	34637707	34637707		Jer	
20	35765175	35765175		Ang	
20	36485236	36485236		Jer	
20	37699755	37699755		Jer	
20	38584191	38584191		Gue	
20	38688854	38688854		Her	
20	39997182	39997182		Jer	
20	40829443	40829443		Her	
20	41664409	41664409		Nor	
20	43351945	43351945		Her	
20	43545648	43545648		Ang	
20	43976492	43976492		Bro	
20	47345125	47345125		Lim	
20	47561347	47561347		Lim	
20	48282061	48282061		Gue	
20	49045314	49045314		Nor	
20	49421139	49421139		Hol	
20	49754059	50263448			Guz
20	49948360	49948360		Hol	
20	49978524	50577363			Nel
20	50609613	50609613		Hol	
20	50685586	50754373			Nel
20	50757998	50757998		Lim	

20	51095242	51095242		Bro
20	55369918	55369918		Ang
20	55994732	55994732		Her
20	56086137	56086137		Lim
20	56375940	56375940		Ang
20	56580787	56580787		Lim
20	57172737	57172737		Jer
20	57569177	57569177		Ang
20	58455297	58455297		Hol
20	58491204	58491204		Her
20	58741188	58741188		Her
20	58878031	58878031		Nor
20	59095135	59095135		Bro
20	59547361	59547361		Gue
20	61703362	61703362		Nor
20	62629282	62629282		Gue
20	63302487	63302487		Nor
20	65891632	65891632		Hol
20	65891632	65891632		Jer
20	66375195	66375195		Nor
20	67161518	67161518		Jer
20	67455096	67455096		Bro
20	69180337	69180337		Lim
20	69538476	69538476		Bro
20	70562140	70562140		Gue
20	70849409	70849409		Gue
20	71069702	71069702		Jer
20	71629018	71967622	ZT	
21	83766	83766	ZT	
21	302276	302276	ZT	
21	410363	410363	ZT	
21	560942	560942	ZT	
21	729486	898385	ZT	
21	774574	774574		Gue
21	1058688	1058688	ZT	
21	1264130	1264130	ZT	
21	1780745	1780745	ZT	
21	2135292	2216087	ZT	
21	2329730	2416432	ZT	
21	3178241	3178241		Hol
21	3653561	3653561		Her
21	4489850	4489850		Gue
21	4802612	4802612		Lim
21	5806951	5806951		Hol
21	6034317	6034317		Ang
21	7136870	7136870		Lim
21	9315185	9315185		Bro
21	9688958	9688958		Gue

21	10328625	10328625		Bro	
21	10843245	10843245		Ang	
21	11018691	11018691		Lim	
21	11285775	11285775		Ang	
21	11715137	11715137		Nor	
21	11996254	11996254		Lim	
21	12173251	12173251		Ang	
21	12634264	12634264		Gue	
21	12767093	12767093		Jer	
21	13630516	13630516		Jer	
21	13775692	13775692		Gue	
21	14407345	14407345		Hol	
21	14474236	14474236		Lim	
21	15079273	15079273		Lim	
21	15414028	15414028		Her	
21	15581199	15581199		Lim	
21	15881556	15881556		Nor	
21	16785248	16785248		Ang	
21	17140806	17140806		Bro	
21	17224297	17224297		Ang	
21	17702229	17702229		Jer	
21	19080172	19080172		Bro	
21	19134133	19134133		Jer	
21	19221246	19221246		Ang	
21	19858774	19858774		Her	
21	20604446	20749275			Nel
21	20669309	20669309		Lim	
21	21407592	21407592		Nor	
21	21460863	21460863		Bro	
21	25775845	25775845		Bro	
21	25858249	25858249		Hol	
21	27632190	27632190		Lim	
21	28346209	28346209		Nor	
21	29966640	29966640		Her	
21	30436035	30436035		Nor	
21	30990428	30990428		Jer	
21	31681776	33273658	ZT		
21	32174987	32174987		Her	
21	33719179	33719179		Jer	
21	34995078	34995078		Her	
21	35336480	35336480		Her	
21	35786021	35786021		Lim	
21	35940053	35940053		Nor	
21	36602306	36602306		Her	
21	36907183	36907183		Jer	
21	37024330	37024330		Ang	
21	38233793	38233793		Hol	
21	38281466	38281466		Nor	

21	38446915	38446915		Bro	
21	39721245	39721245		Nor	
21	41402002	42458641			Gir
21	41676419	41676419		Ang	
21	42253894	42253894		Bro	
21	42671492	42671492		Her	
21	43716780	43716780		Bro	
21	43835814	43835814		Bro	
21	44055266	44055266		Gue	
21	44190088	44190088		Ang	
21	44310760	44310760		Gue	
21	45435950	45435950		Bro	
21	45793883	45979589	ZT		
21	47785165	47854533			Nel
21	48674506	48674506		Lim	
21	48834422	48834422		Nor	
21	51263827	51263827		Jer	
21	51632410	51632410		Jer	
21	52092528	52092528		Nor	
21	52286546	52286546		Jer	
21	52338607	52338607		Hol	
21	52524822	52524822		Jer	
21	52938578	52938578		Her	
21	53153813	53153813		Gue	
21	53324291	53324291		Gue	
21	54540263	54540263		Bro	
21	54600298	54600298		Lim	
21	55636264	55636264		Nor	
21	56136037	56136037		Ang	
21	56634361	56634361		Her	
21	57365818	57365818		Nor	
21	57657495	57657495		Ang	
21	57791376	57791376		Hol	
21	57791376	57791376		Jer	
21	59091888	59091888		Bro	
21	59091888	59091888		Hol	
21	59380074	59380074		Lim	
21	59679183	59679183		Jer	
21	61068120	61068120		Ang	
21	63646469	63646469		Jer	
21	64139734	64139734		Ang	
21	64216060	64216060		Gue	
21	64295557	64295557		Ang	
21	64366520	64366520		Nor	
21	64788279	64788279		Ang	
21	65151031	65151031		Jer	
21	66893391	66893391		Bro	
21	68314240	68314240		Gue	

21	68349152	68943249	ZT		
21	68782355	68782355		Ang	
21	69171066	69171066		Hol	
21	69505292	69505292		Her	
21	69651875	69651875		Jer	
21	70202199	70401009			Nel
21	70228643	70228643		Her	
21	70268849	70268849		Jer	
21	70384755	70384755		Bro	
21	70566220	70566220		Hol	
21	71237182	71237182		Jer	
21	71464920	71464920		Ang	
22	223875	223875		Her	
22	1634108	1634108		Jer	
22	2922700	2922700		Gue	
22	3215464	3215464		Gue	
22	4514702	4514702		Nor	
22	4763900	4763900		Her	
22	5004055	5004055		Her	
22	5144693	5144693		Ang	
22	5253524	5253524		Lim	
22	8687590	8687590		Gue	
22	9064466	9064466		Nor	
22	9825898	9825898		Lim	
22	10714182	10714182		Her	
22	12034774	12034774		Gue	
22	14046942	14046942		Gue	
22	14139516	14139516		Jer	
22	14991411	14991411		Hol	
22	15281286	15330491			Nel
22	15476132	15476132			Gir
22	15476132	15476132			Nel
22	15589177	15924030			Gir
22	15589177	15924030			Nel
22	16739072	16739072		Her	
22	16739072	16739072		Hol	
22	17380447	17629960			Nel
22	17734414	17790781			Nel
22	18748905	18748905		Bro	
22	18778517	18778517		Ang	
22	18778517	18778517		Jer	
22	18978658	18978658		Ang	
22	19575681	19575681		Jer	
22	19773432	19773432		Nor	
22	21121244	21121244		Nor	
22	21788372	21788372		Her	
22	22869809	22869809		Nor	
22	22986987	22986987		Hol	

22	23186972	23186972	Bro	
22	24389805	24389805	Hol	
22	24938846	24938846	Her	
22	25228823	25228823	Her	
22	26157144	26157144	Nor	
22	26919242	26919242	Gue	
22	27121386	27121386	Ang	
22	27417822	27417822	Gue	
22	27503111	27503111	Ang	
22	27503111	27503111	Hol	
22	28626857	28626857	Jer	
22	29096738	29096738	Nor	
22	29802653	29802653	Her	
22	32231307	32231307	Hol	
22	32886762	32886762	Ang	
22	33098772	33098772	Her	
22	33382808	33382808	Lim	
22	33437808	33437808	Nor	
22	33956188	33956188	Jer	
22	34113952	34113952	Hol	
22	34954193	34954193	Lim	
22	35363041	35363041	Gue	
22	36110577	36110577	Lim	
22	36631130	36631130	Lim	
22	36804226	36804226	Her	
22	37615930	37615930	Her	
22	37739457	37739457	Bro	
22	38544707	38544707	Bro	
22	39116221	39116221	Bro	
22	39448055	39448055	Hol	
22	39579899	39579899	Jer	
22	41276173	41276173	Ang	
22	41990501	41990501	Hol	
22	43019540	43019540	Ang	
22	43104089	43104089	Lim	
22	43194827	43194827	Her	
22	44489267	44945838		Gir
22	45047902	45381866		Gir
22	45513779	45513779		Gir
22	45548761	45548761	Her	
22	45618606	45670270		Gir
22	45795690	45795690		Gir
22	45903075	45903075		Gir
22	46184881	46184881	Bro	
22	46885785	46885785	Jer	
22	47134013	47134013	Ang	
22	48866088	48943916		Gir
22	49058093	49058093		Gir

22	49169752	49376919		Gir
22	49228279	49228279	Her	
22	49250533	49250533	Bro	
22	50102342	50102342	Hol	
22	52092282	52092282	Ang	
22	52398798	52398798	Lim	
22	52426809	52520543	Hol	
22	53858879	53858879	Gue	
22	54471732	54471732	Hol	
22	54756606	54756606	Nor	
22	55284827	55284827	Her	
22	56358873	56358873	Lim	
22	56543107	56543107	Nor	
22	56650378	56650378	Ang	
22	56650378	56650378	Hol	
22	56677054	56677054	Nor	
22	56719491	56719491	Bro	
22	56901581	56901581	Jer	
22	57284409	57284409	Nor	
22	59676713	59676713	Lim	
22	61340279	61340279	Lim	
23	1722267	1722267	Nor	
23	3130810	3130810	Nor	
23	6568421	6568421	Gue	
23	6604188	6604188	Hol	
23	6901457	6958671	Ang	
23	7122656	7122656	Gue	
23	7418519	7418519	Her	
23	7469550	7563884		Guz
23	8515946	8515946	Gue	
23	9240908	9240908	Bro	
23	9408634	9408634	Lim	
23	9553885	9553885	Bro	
23	9705388	9705388	Lim	
23	10866245	10866245	Gue	
23	11254502	11254502	Gue	
23	12024909	12024909	Ang	
23	12102265	12102265	Lim	
23	12257262	12257262	Hol	
23	12449291	12449291	Nor	
23	12569920	12569920	Lim	
23	15061292	15061292	Nor	
23	15686158	15686158	Lim	
23	16038896	16038896	Jer	
23	17081775	17081775	Bro	
23	17502522	17502522	Ang	
23	17598366	17598366	Lim	
23	19178586	19178586	Lim	

23	19290469	19290469	Her
23	19712427	19712427	Jer
23	19801163	19801163	Ang
23	20217537	20217537	Hol
23	21047788	21047788	Hol
23	21375181	21375181	Her
23	23388685	23388685	Hol
23	23557329	23557329	Her
23	23770678	23770678	Hol
23	24210610	24210610	Hol
23	25119525	25119525	Her
23	25208222	25208222	Ang
23	27003561	27003561	Hol
23	28382993	28382993	Nor
23	28568611	28568611	Jer
23	28638238	28638238	Ang
23	29393009	29393009	Bro
23	29481801	29481801	Hol
23	29887010	29887010	Bro
23	30163726	30163726	Nor
23	32374089	32374089	Bro
23	32805879	32805879	Ang
23	33116927	33116927	Her
23	33308775	33389104	Nor
23	35844276	35844276	Lim
23	36005120	36005120	Lim
23	36873947	36873947	Ang
23	37621058	37621058	Gue
23	38108739	38137505	Jer
23	39009221	39009221	Lim
23	39236560	39236560	Nor
23	39416740	39416740	Bro
23	39416740	39416740	Lim
23	41431432	41431432	Hol
23	42675687	42675687	Ang
23	42675687	42675687	Gue
23	43348970	43348970	Ang
23	44493427	44493427	Bro
23	44493427	44493427	Lim
23	46147244	46147244	Bro
23	49219258	49219258	Gue
23	49257068	49257068	Hol
23	49766555	49766555	Ang
23	49766555	49766555	Gue
23	49936797	49936797	Bro
23	50696236	50696236	Jer
23	51482321	51482321	Her
23	52113879	52113879	Lim

24	595437	595437		Ang
24	1931077	1931077		Her
24	1931077	1931077		Jer
24	3728431	3728431		Her
24	3860135	3940751		Her
24	4003577	4003577		Hol
24	4069308	4069308		Bro
24	4481598	4481598		Hol
24	4552001	4552001		Nor
24	6464111	6464111		Gue
24	6592653	6592653		Lim
24	6653759	6653759		Hol
24	6815805	6815805		Gue
24	6815805	6815805		Jer
24	7046195	7046195		Hol
24	7268842	7268842		Jer
24	7414910	7414910		Bro
24	8475964	8475964		Gue
24	9018756	9018756		Her
24	9325287	9325287		Nor
24	10184151	10184151		Gue
24	11042693	11042693		Lim
24	11671411	11671411		Jer
24	12035684	12035684		Nor
24	12109566	12109566		Jer
24	12689748	12689748		Ang
24	13980307	13980307		Hol
24	14453986	14453986		Nor
24	14550660	14550660		Ang
24	14654716	14654716		Nor
24	14955777	14955777		Lim
24	15085174	15085174		Lim
24	15157104	15157104		Ang
24	15157104	15157104		Hol
24	17781574	17781574		Nor
24	20181440	20181440		Gue
24	20328632	20328632		Jer
24	21735980	21735980		Nor
24	22959432	22959432		Bro
24	24114816	24452344	ZT	
24	24126741	24126741		Nor
24	25268157	25327416		Nor
24	25327416	25327416		Ang
24	25475172	25475172		Ang
24	25940349	25940349		Bro
24	26180396	26180396		Lim
24	27163570	27163570		Lim
24	27850482	27850482		Bro

24	28131181	28131181		Jer
24	28294938	28294938		Hol
24	29464711	29464711		Nor
24	29734324	29734324		Nor
24	30135742	30135742		Ang
24	30737579	30737579		Nor
24	31555588	31555588		Her
24	31869672	31869672		Her
24	32463032	32794826		Cha
24	33307670	33307670		Bro
24	33779670	33779670		Lim
24	36487225	36487225		Lim
24	37927189	37927189		Lim
24	40140343	40140343		Bro
24	40304952	40304952		Lim
24	40928928	40928928		Jer
24	41922106	41922106		Gue
24	41922106	41922106		Nor
24	42875547	42925107	ZT	
24	43649003	43649003		Lim
24	43770409	43824028		Her
24	45655306	45655306		Bro
24	45813402	45837548		Jer
24	46053659	46053659		Hol
24	47419901	47419901		Gue
24	47440919	47440919		Bro
24	47742388	47742388		Jer
24	49794284	49794284		Nor
24	51988098	51988098		Bro
24	52426314	52426314		Jer
24	53228187	53228187		Hol
24	53338779	53338779		Her
24	53528690	53528690		Nor
24	55104948	55104948		Bro
24	55497641	55497641		Hol
24	57098899	57098899		Her
24	57341186	57341186		Gue
24	57764659	57764659		Her
24	57950814	57950814		Nor
24	58173824	58173824		Gue
24	58457858	58457858		Bro
24	59072755	59072755		Nor
24	59179876	59179876		Her
24	59598481	59598481		Bro
24	60249445	60249445		Ang
24	60249445	60249445		Jer
24	61270542	61270542		Her
24	61356757	61356757		Hol

24	61931908	61931908	Lim
25	2226168	2226168	Nor
25	3267124	3267124	Nor
25	4599247	4599247	Nor
25	5196611	5196611	Nor
25	6304451	6304451	Gue
25	7084164	7084164	Jer
25	8780012	8780012	Nor
25	9056548	9056548	Her
25	9056548	9056548	Hol
25	9823462	9823462	Bro
25	11518224	11518224	Nor
25	11647827	11647827	Jer
25	12965823	12965823	Nor
25	13991913	13991913	Ang
25	13991913	13991913	Gue
25	13991913	13991913	Nor
25	14162395	14162395	Gue
25	14971307	14971307	Ang
25	15362445	15362445	Nor
25	17188592	17188592	Her
25	19599194	19599194	Gue
25	20377310	20377310	Ang
25	21179530	21179530	Her
25	21420847	21420847	Nor
25	21530729	21530729	Bro
25	21583665	21583665	Gue
25	21712001	21712001	Bro
25	23311252	23311252	Nor
25	25159449	25159449	Ang
25	26469684	26469684	Gue
25	26752172	26752172	Jer
25	28100092	28100092	Bro
25	28739966	28739966	Lim
25	28841121	28841121	Bro
25	29003225	29044566	Nor
25	30552788	30552788	Ang
25	32021837	32021837	Nor
25	33829538	33829538	Ang
25	34117267	34117267	Jer
25	35900579	35900579	Lim
25	36003554	36003554	Her
25	36003554	36003554	Hol
25	37687592	37687592	Hol
25	38007215	38007215	Ang
25	38554362	38554362	Lim
25	38794162	38839907	Ang
25	38839907	38839907	Hol

25	41045423	41045423	Jer
25	42179355	42179355	Jer
26	4004822	4004822	Nor
26	4357955	4357955	Jer
26	4837610	4837610	Her
26	5186735	5186735	Ang
26	6798521	6798521	Nor
26	8573229	8573229	Ang
26	10255258	10255258	Bro
26	11847117	11847117	Ang
26	13814193	13814193	Hol
26	15896988	15896988	Gue
26	17296240	17296240	Her
26	17851349	17851349	Gue
26	17890705	17890705	Her
26	18420542	18420542	Her
26	18530826	18530826	Jer
26	19276542	19276542	Jer
26	19294094	19294094	Lim
26	19580383	19580383	Bro
26	20364907	20364907	Jer
26	21143532	21143532	Gue
26	21461562	21461562	Hol
26	22925118	22925118	Ang
26	23335919	23335919	Gue
26	23491390	23491390	Nor
26	24881049	24881049	Ang
26	25257862	25322990	Lim
26	25994062	25994062	Ang
26	26477459	26477459	Bro
26	26715647	26715647	Ang
26	27248024	27248024	Her
26	27492649	27492649	Hol
26	27874379	27874379	Nor
26	28930038	28930038	Her
26	29108559	29108559	Ang
26	29232134	29232134	Nor
26	31197625	31197625	Ang
26	32514379	32514379	Jer
26	33003665	33003665	Jer
26	33544329	33544329	Ang
26	33919440	33919440	Nor
26	34331333	34331333	Bro
26	34388222	34388222	Her
26	34388222	34388222	Hol
26	34830744	34830744	Gue
26	34979362	34979362	Lim
26	35839349	35839349	Ang

26	36370940	36370940	Bro	
26	36696640	36696640	Nor	
26	36970886	36970886	Ang	
26	36970886	36970886	Gue	
26	36970886	36970886	Nor	
26	37599626	37999778		Guz
26	37865959	37865959	Her	
26	37917187	38047818		Nel
26	38137742	38137742	Gue	
26	39780004	39780004	Her	
26	39876353	39876353	Ang	
26	40733253	40733253	Jer	
26	41317644	41317644	Bro	
26	41815981	41815981	Gue	
26	42441667	42441667	Gue	
26	42579324	42579324	Lim	
26	43314317	43314317	Hol	
26	43739858	43739858	Ang	
26	44065654	44065654	Nor	
26	45265647	45265647	Hol	
26	45414142	45414142	Nor	
26	45609721	45609721	Jer	
26	45860522	45860522	Her	
26	46892521	46892521	Her	
26	46962007	46962007	Nor	
26	47147283	47147283	Gue	
26	47868995	47868995	Lim	
26	48634032	48634032	Ang	
26	48678090	48678090	Bro	
26	48946233	48946233	Gue	
26	49102906	49102906	Bro	
26	49464781	49464781	Nor	
26	50504201	50504201	Jer	
26	50641748	50641748	Lim	
26	50874925	50874925	Nor	
26	50957350	50957350	Jer	
26	51552739	51552739	Hol	
27	198019	198019	Bro	
27	1644121	1644121	Her	
27	2152791	2152791	Nor	
27	2427007	2427007	Nor	
27	2722382	2722382	Hol	
27	2918822	2918822	Bro	
27	3042504	3042504	Gue	
27	4656302	4656302	Bro	
27	5067846	5067846	Bro	
27	5407238	5407238	Gue	
27	6289689	6289689	Ang	

27	6289689	6289689	Gue
27	6289689	6289689	Hol
27	7809061	7809061	Her
27	8666615	8666615	Her
27	9100139	9100139	Nor
27	9498160	9498160	Gue
27	11570761	11570761	Her
27	12761372	12761372	Ang
27	14684871	14684871	Bro
27	15129280	15129280	Bro
27	15316486	15316486	Gue
27	15488427	15488427	Nor
27	16237074	16237074	Nor
27	16495466	16495466	Lim
27	16732706	16732706	Her
27	17504630	17504630	Ang
27	17504630	17504630	Nor
27	19351133	19351133	Her
27	19422656	19422656	Nor
27	20898312	20898312	Nor
27	22268519	22268519	Ang
27	22364920	22364920	Her
27	22604390	22604390	Her
27	22757505	22757505	Bro
27	22865771	22865771	Jer
27	23120365	23120365	Nor
27	23160424	23160424	Lim
27	24030500	24030500	Bro
27	24311520	24386563	Hol
27	24433603	24433603	Ang
27	25383890	25383890	Her
27	25529207	25529207	Her
27	31210394	31210394	Jer
27	31233518	31233518	Lim
27	31588911	31588911	Lim
27	32027251	32027251	Lim
27	34047531	34047531	Hol
27	35032230	35032230	Her
27	35389008	35389008	Jer
27	37223964	37223964	Nor
27	37970643	37970643	Jer
27	38593377	38593377	Hol
27	38593377	38593377	Jer
27	39076960	39076960	Lim
27	39237771	39237771	Jer
27	39761980	39761980	Lim
27	40019304	40019304	Hol
27	40144051	40144051	Gue

27	40395249	40395249	Her	
27	40698638	40698638	Gue	
27	40739599	40739599	Hol	
27	41298206	41298206	Gue	
27	41323268	41323268	Hol	
27	42224267	42224267	Lim	
27	42968597	42968597	Gue	
27	43026425	43026425	Bro	
27	43711279	43711279	Her	
27	44056399	44056399	Hol	
27	44130007	44130007	Lim	
27	44518346	44518346	Her	
27	44795496	44795496	Jer	
27	44952306	44952306	Lim	
28	685362	685362	Gue	
28	1522477	1522477	Ang	
28	2197476	2197476	Gue	
28	2197476	2197476	Nor	
28	3261517	3402458		Nel
28	3902035	3902035	Hol	
28	5250484	5250484	Jer	
28	6783645	6783645	Lim	
28	6863680	6863680	Her	
28	7439585	7439585	Gue	
28	8080087	8080087	Gue	
28	8354512	8354512	Jer	
28	8395160	8395160	Gue	
28	8590626	8590626	Hol	
28	8671561	8671561	Gue	
28	9657827	9657827	Gue	
28	10527491	10527491	Her	
28	10954154	10954154	Nor	
28	12660161	12660161	Hol	
28	13302247	13302247	Nor	
28	13429871	13429871	Jer	
28	13594431	13594431	Gue	
28	16291445	16291445	Jer	
28	17228071	17228071	Jer	
28	18186248	18186248	Hol	
28	18816874	18816874	Gue	
28	18971778	18971778	Lim	
28	20951400	20951400	Ang	
28	20951400	20951400	Nor	
28	21027360	21027360	Lim	
28	21833628	21833628	Nor	
28	23045649	23045649	Nor	
28	23457159	23457159	Her	
28	23632432	23632432	Bro	

28	27333444	27333444	Nor	
28	27695848	27695848	Nor	
28	27984648	27984648	Hol	
28	28030026	28030026	Bro	
28	28106435	28106435	Lim	
28	28395949	28395949	Bro	
28	28767391	28767391	Her	
28	29116183	29116183	Lim	
28	29394116	29612623		Gir
28	29734099	29734099		Gir
28	30323737	30323737	Bro	
28	30658103	30658103	Her	
28	30830682	30830682	Nor	
28	31084814	31084814	Her	
28	31364396	31364396	Nor	
28	31917001	31917001	Ang	
28	32035385	32035385	Bro	
28	32681073	32681073	Hol	
28	33355851	33355851	Nor	
28	33449126	33449126	Her	
28	34313489	34313489	Gue	
28	34434650	34434650	Ang	
28	34510944	34510944	Jer	
28	35025032	35025032	Hol	
28	35286017	35286017	Her	
28	36647755	36647755	Hol	
28	36949147	36949147	Bro	
28	37919850	37919850	Hol	
28	38369435	38369435	Lim	
28	38739206	38739206	Jer	
28	39109829	39109829	Lim	
28	39506736	39506736	Nor	
28	40124664	40124664	Gue	
28	40325483	40325483	Bro	
28	40325483	40325483	Jer	
28	40325483	40325483	Lim	
28	40579464	40579464	Nor	
28	40835122	40835122	Gue	
28	40835122	40835122	Jer	
28	40984324	40984324	Lim	
28	41738453	41738453	Gue	
28	43328482	43328482	Gue	
28	43440375	43440375	Gue	
28	43882171	43882171	Nor	
28	44483670	44483670	Jer	
28	44713619	44713619	Hol	
28	45521696	45521696	Her	
28	45701490	45701490	Nor	

28	46151137	46151137	Lim
29	2381798	2381798	Her
29	6689993	6689993	Her
29	7314334	7314334	Ang
29	7314334	7314334	Nor
29	7357191	7357191	Lim
29	7960759	7960759	Bro
29	8013686	8013686	Lim
29	8249041	8249041	Her
29	8919227	8919227	Hol
29	9496843	9496843	Jer
29	9996897	9996897	Her
29	9996897	9996897	Hol
29	10267644	10267644	Ang
29	11254870	11254870	Bro
29	12405803	12405803	Her
29	13286538	13286538	Hol
29	13388874	13388874	Gue
29	14267174	14267174	Gue
29	14777349	14777349	Gue
29	15007220	15007220	Lim
29	15389832	15389832	Gue
29	15419837	15419837	Hol
29	15604832	15604832	Nor
29	16519520	16519520	Bro
29	16563370	16563370	Ang
29	16832642	16832642	Gue
29	17470586	17470586	Her
29	19811305	19811305	Her
29	21856094	21856094	Her
29	25589023	25589023	Ang
29	26432802	26432802	Gue
29	26580551	26580551	Jer
29	27594621	27594621	Ang
29	28592323	28592323	Jer
29	28625093	28625093	Ang
29	30043204	30043204	Bro
29	30100272	30100272	Jer
29	30144517	30144517	Hol
29	31013389	31013389	Jer
29	31123926	31123926	Ang
29	31229505	31229505	Gue
29	32644310	32644310	Ang
29	33972280	33972280	Her
29	34027829	34027829	Nor
29	34432438	34432438	Gue
29	35799111	35799111	Gue
29	38767346	38767346	Gue

29	39781860	39781860		Lim	
29	40558177	40558177		Ang	
29	40558177	40558177		Nor	
29	40823987	40823987		Lim	
29	40901491	40901491		Her	
29	41324292	41324292		Hol	
29	43825229	43825229		Jer	
29	44224205	44224205		Ang	
29	44323545	44323545		Hol	
29	44494117	44775664			Gir
29	45117874	45117874		Jer	
29	47030125	47030125		Jer	
29	48475112	48475112		Nor	
29	48591282	48591282		Jer	
29	48766169	48766169		Hol	
29	49309125	49309125		Nor	
29	50106335	50106335		Gue	
29	51078181	51078181		Nor	
29	51452986	51452986	ZT		
