# Supplementary material for: Genomic divergence of zebu and taurine cattle identified through high-density SNP genotyping
Source: BMC Genomics. 2013 Dec 13;14(1):876. doi: 10.1186/1471-2164-14-876 (PMC4046821; doi:10.1186/1471-2164-14-876)
Supplement: Supplementary file 7 — Additional file 7: Table S4: Candidate regions for balancing selection: bottom 1% smoothed FST values for all breeds in all analyses. (PDF 769 KB) [file 12864_2012_5571_MOESM7_ESM.pdf]

Supplementary table 4. Summary of candidate regions in autosomes of balancing selection detected using smoothed FST in three analyses: 1) comparing zebu-taurine animals, 2) within taurine (n = 9 breeds), and 3) within zebu (n = 3 breeds).

The bottom 1% smoothed FST values for each breed in each analysis were considered peaks. For each analyses separately, if two breed specific peaks were less than 50Kb from each other they were joined into a single peak-region. In this table no attempt was made to join any breed specific peak-regions or to identify peak-regions in common (or overlaps) across breeds and across the different analyses.

BTA	Start	End	Zebu-Taurine	Within_taurine	Within_zebu
1	2037672	2352532		Lim	
1	3579259	3643368			Nel
1	3940733	4010489		Jer	
1	4067489	4067489			Nel
1	4170042	4170042			Nel
1	4295157	4410834			Nel
1	4530391	4530391			Nel
1	4812893	4918544			Gir
1	6835494	7165460		Cha	
1	9521109	9726816		Cha	
1	10392995	10541635		Jer	
1	11218497	11441406		Nor	
1	12332161	12380238		Gue	
1	12488078	12739533		Gue	
1	13492766	13908814		Nor	
1	15766553	15921372		Jer	
1	17070285	17652453		Lim	
1	20921793	21085526		Jer	
1	28716153	28981370		Lim	
1	28939097	29393631		Ang	
1	34586544	34586544			Guz
1	36710870	37100951		Cha	
1	37207407	37207407		Cha	
1	37207407	37271958		Lim	
1	39716103	39910369		Lim	
1	40014655	40096011		Lim	
1	44045405	44514015		Lim	
1	45893364	45942600			Nel
1	46056895	46135864			Nel
1	46258910	46258910			Nel
1	54988164	55364825			Guz
1	55901366	55998138		Gue	
1	58118007	58304127		Lim	
1	64338202	64387526		Nor	
1	64608435	65126749		Ang	
1	66490101	66701323		Ang	
1	66527937	67416313			Nel
1	66659645	66701323		Nor	

1	66814280	66814280	Ang	
1	67679688	68592048	Cha	
1	68276649	68661496	Ang	
1	69119625	69180180		Nel
1	70469092	70843771		Nel
1	70925090	70977963	Nor	
1	76704597	76917631		Nel
1	82192085	82240344	Cha	
1	83195623	83195623		Guz
1	83242738	83626527	Bro	
1	83348654	83538104		Guz
1	83733826	83903856	Bro	
1	84030978	84030978	Bro	
1	84188608	84418399	Bro	
1	87301706	87583573		Gir
1	87711085	88493112		Gir
1	88366327	88493112		Nel
1	88595430	88595430		Nel
1	89253733	89495325		Gir
1	90889100	91145158		Gir
1	91264694	91474650		Gir
1	91948784	92158374		Nel
1	92493101	92680870	Cha	
1	92869117	92936159	Cha	
1	95477262	95549663		Guz
1	96955573	97106632		Guz
1	98133752	98193221		Guz
1	102628793	103033809	Lim	
1	104066487	104160954	Cha	
1	105264358	105390377	Jer	
1	105475769	106184789	Hol	
1	106290322	106400046	Hol	
1	106431991	106514910	Her	
1	107854532	108189849		Guz
1	107854532	108006588		Nel
1	107881814	108260465	Hol	
1	108260465	108480479	Lim	
1	108428021	108428021	Her	
1	109302787	109427567	Lim	
1	111750865	111954606	Jer	
1	113827584	113906808	Bro	
1	114008939	114348164	Bro	
1	114008939	114263777		Gir
1	115427475	115793142	Hol	
1	118826155	119158995		Nel
1	118959520	119196385		Guz
1	123033120	123033120	Gue	
1	123148441	123223792	Gue	

1	126561061	126679598	Her	
1	126994435	127206157	Nor	
1	127429635	127905071	Bro	
1	127635326	127769440	Lim	
1	132612093	132735977	Nor	
1	139519066	139680260	Gue	
1	139823433	140009270	Cha	
1	140709356	141032878	Gue	
1	141158670	141158670	Gue	
1	141272688	141371753	Gue	
1	141850538	142110106		Gir
1	142006688	142284552	Cha	
1	144306464	144429157	Bro	
1	144487373	144682686		Nel
1	144639075	144722440	Gue	
1	146464733	146464733	Jer	
1	147262537	147314194		Guz
1	149995600	149995600	Cha	
1	150187027	150246003	Nor	
2	1039834	1069251	Nor	
2	1069251	1120956	Her	
2	4637012	5149149	Jer	
2	11814955	11856020	Her	
2	11884273	12071031		Nel
2	24285533	24350788	Nor	
2	26357124	26357124	Ang	
2	31195297	31528039	Lim	
2	34673859	34919470	Jer	
2	35144463	35374515		Nel
2	35472241	35880237	Lim	
2	36841417	37176489	Her	
2	37803158	37803158	Hol	
2	37922119	37922119	Hol	
2	40386188	40664544	Hol	
2	40987473	41125455	Bro	
2	41228198	41228198	Bro	
2	42862918	43458347	Bro	
2	42929914	43367798	Ang	
2	43220655	43669764	Nor	
2	44242869	44301656	Gue	
2	47484517	47723309	Lim	
2	56427965	56594393	Ang	
2	56796392	56818605	Hol	
2	60617679	60977906	Ang	
2	61876359	62211779		Guz
2	62040805	62093948	Jer	
2	62957859	63162563		Guz
2	65236181	65695312	Ang	

2	68969639	69770834	Gue	
2	69893248	70902064	Gue	
2	69947393	69947393	Ang	
2	71185300	72088161	Nor	
2	71819182	72154737	Jer	
2	71821926	72461152		Nel
2	73136334	73295397	Lim	
2	73136334	73235821	Nor	
2	73404896	73404896	Lim	
2	74005555	74202772	Lim	
2	74270829	74708745	Gue	
2	74565316	74708745	Lim	
2	76794789	76794789	Jer	
2	76872648	77063880		Nel
2	76970656	76970656	Nor	
2	77063880	77246194		Gir
2	77175718	77175718	Jer	
2	77423199	78007011	Jer	
2	78161232	78161232	Her	
2	78556325	78556325	Jer	
2	79413457	79522297	Ang	
2	79413457	79490369	Jer	
2	79633085	79633085	Ang	
2	79738886	80298164	Ang	
2	79738886	79901540	Jer	
2	80603725	80708386	Jer	
2	81335062	81461797	Jer	
2	81645767	81721478	Ang	
2	81844987	82840808	Ang	
2	82928052	83595986	Nor	
2	83712390	84143473	Nor	
2	84023099	84093028	Gue	
2	84270003	84348194	Nor	
2	90574020	90669806	Jer	
2	99405576	99661871	Gue	
2	100342719	100342719	Gue	
2	102318489	102782167	Gue	
2	113242573	113310877	Hol	
2	125375690	125375690	Nor	
2	125489097	125890340	Nor	
2	130716998	130865106	Bro	
3	4368671	4487413	Cha	
3	4588145	4615611	Cha	
3	6217109	6276686	Cha	
3	7184450	7266768	Cha	
3	8984749	9094992	Gue	
3	9221939	9281831	Gue	
3	9388253	9478846	Gue	

3	10641889	11142773	Her	
3	11557412	11930318		Gir
3	12159772	12159772		Gir
3	12275673	12516312		Gir
3	12323598	12708985	Gue	
3	12377695	12653440	Her	
3	12635306	12915639		Gir
3	14014602	14288655	Gue	
3	14288655	14501847	Her	
3	14853592	15441760	Gue	
3	14983356	15548277	Bro	
3	15076675	15265624	Nor	
3	15218694	15410466	Her	
3	15410466	15660317	Cha	
3	19308579	19385035		Gir
3	19328506	19646785	Bro	
3	19396423	19704580	Lim	
3	19522686	19522686		Gir
3	28856107	28998296	Hol	
3	29104477	29104477	Hol	
3	30040048	30040048	Ang	
3	30040048	30216573	Jer	
3	30309723	30331825	Hol	
3	34300912	34300912	Ang	
3	34424540	34512042	Ang	
3	35521695	35785053	Bro	
3	37800799	37800799	Ang	
3	37904788	38097870	Ang	
3	41566030	41640521	Hol	
3	43262046	43687651	Lim	
3	43528755	43562824	Gue	
3	57099499	57200004	Cha	
3	57407830	57477853	Lim	
3	61486775	61955379		Guz
3	65981531	66533414		Guz
3	66089009	66533414		Gir
3	66650037	66901916		Gir
3	66650037	66901916		Guz
3	67003456	67419002		Gir
3	67003456	67367498		Guz
3	67216388	67447860	Hol	
3	67338055	67427479	Gue	
3	67478877	67533434	Her	
3	75170259	75329394		Nel
3	75397165	75718184		Gir
3	75567932	75617933	Lim	
3	76045719	76262584		Nel
3	76447030	76795293	Jer	

3	77129465	77727223	Jer	
3	78151023	78213770	Jer	
3	79246696	79379819	Nor	
3	90342708	90417369	Hol	
3	93288452	93375163	Lim	
3	94181287	94583211	Lim	
3	96197501	96250668	Jer	
3	99126486	99718312	Her	
3	99823337	99823337	Her	
3	109332540	109692820	Jer	
3	110015096	110265914	Gue	
3	110538634	110636957	Bro	
3	115439388	115523479		Nel
3	115491592	115587197	Ang	
3	117549230	117580949	Ang	
3	119789440	119827826	Ang	
4	2548604	2666815		Nel
4	5157612	5477058		Guz
4	5586084	5586084		Guz
4	6248795	6578847		Gir
4	9417524	9505717		Nel
4	9612574	9779560		Nel
4	12045878	12496515		Nel
4	29081138	29081138	Jer	
4	29273904	29273904		Gir
4	29420798	29673705		Gir
4	29791365	30139557		Gir
4	29980543	30032467	Jer	
4	30252603	30252603		Gir
4	31742053	31901772		Nel
4	34909242	35142339	Cha	
4	35335600	36012109	Cha	
4	38538545	38538545	Her	
4	41316989	41456792		Guz
4	41569498	41603469		Guz
4	45762129	46154398	Bro	
4	46053489	46175467		Guz
4	46053489	46175467		Nel
4	47198262	47277331		Guz
4	48310001	48414247		Guz
4	48523422	48656607		Guz
4	48585020	48615363	Cha	
4	48718180	48718180	Cha	
4	48767482	48767482		Guz
4	48839792	48879377	Cha	
4	52649605	52954149	Jer	
4	52791137	53013177		Guz
4	57573757	57573757	Her	

4	57851919	57978926			Nel
4	64098755	64296439			Gir
4	64403984	64761593			Gir
4	64761593	64761593			Nel
4	64879673	64969611			Nel
4	68527851	68527851		Bro	
4	68750153	68832680		Hol	
4	70147152	70262792		Bro	
4	70262792	70395024		Gue	
4	70469620	70664476		Hol	
4	70508946	70656523			Gir
4	70632396	70717821		Lim	
4	72130878	72344551			Nel
4	72584147	72814820			Guz
4	72774164	72948376			Nel
4	72990437	73112667		Gue	
4	73222260	73302220		Gue	
4	83316121	83802875			Guz
4	83929999	84051842			Guz
4	84164532	84164532			Guz
4	84307106	84307106			Guz
4	84418065	84418065			Guz
4	84527948	85133731			Guz
4	85992357	86021183		Cha	
4	91208071	91208071		Lim	
4	92242777	92522721		Her	
4	92672093	93058899			Guz
4	92910098	93370273			Gir
4	93058899	93078720			Nel
4	93475999	93475999			Gir
4	94646302	94646302			Guz
4	98427600	98552147			Nel
4	102427721	102513433			Gir
4	104791437	105260800		Hol	
4	105204914	105448770		Lim	
4	105481885	105532313		Her	
4	105905928	106043564		Jer	
4	106564443	106633751		Hol	
4	106824046	106824046		Hol	
4	108733336	108872954			Guz
4	108835578	108863093		Jer	
4	110295764	111277799	ZT		
4	111378106	111378106	ZT		
4	111742866	112562902	ZT		
4	112975792	113362873		Bro	
4	114538317	114636819		Bro	
4	114565961	115354564		Hol	
4	115217092	115305733			Nel

4	116281024	116383880			Nel
4	116352975	116369242		Nor	
4	116457483	117264852		Bro	
4	117660545	118160445			Gir
4	118168607	118225601		Her	
4	119201399	119291918			Nel
4	119475712	119789473			Gir
4	120082962	120437782		Hol	
4	120082962	120416517			Guz
4	120082962	120581988			Nel
5	1562117	1648232			Guz
5	6083959	6315172		Hol	
5	19457756	19898237	ZT		
5	25902946	25953158			Nel
5	26714225	26967739			Nel
5	27216778	27401494		Jer	
5	30395381	30395381		Nor	
5	31825300	31949666		Jer	
5	37799897	37866320			Gir
5	39196424	39500339		Gue	
5	40366855	40637761		Cha	
5	43060961	43185507			Guz
5	47305323	47433954			Gir
5	47587701	47756295			Nel
5	54447666	54494300		Hol	
5	64506830	64613619		Lim	
5	65858094	66035895		Jer	
5	72979259	73335888		Nor	
5	74182928	74288348			Guz
5	76719327	76811165	ZT		
5	76972837	77207435	ZT		
5	87507444	87859948			Gir
5	89876954	89876954		Bro	
5	89994551	90192310		Bro	
5	91107601	91403113		Hol	
5	95306454	95306454		Her	
5	95410443	95459836		Her	
5	96475709	96764402			Gir
5	99198859	99379658		Ang	
5	104194524	104233341		Jer	
5	105477063	105758404		Jer	
5	108820698	109069968		Lim	
5	108820698	109129238		Nor	
5	109796357	110046386		Bro	
5	110258274	110310349		Nor	
5	111599240	112093369		Cha	
5	113491995	113505486		Bro	
5	114319413	114526589		Gue	

5	114319413	114430942		Jer	
5	114430942	114661464		Cha	
5	115171406	115171406		Jer	
5	115793651	115869934		Jer	
5	116804850	116882796		Ang	
6	449903	449903		Ang	
6	878249	1303767		Lim	
6	1405878	1604556		Lim	
6	2883313	3217181	ZT		
6	3358168	4231143	ZT		
6	9558946	9746897		Cha	
6	9850754	10287413		Cha	
6	9941278	10147796		Jer	
6	10671286	10958234		Bro	
6	11053007	11287112			Guz
6	11656479	11704613			Gir
6	12490545	13266473	ZT		
6	13857569	14030629		Bro	
6	19155106	19187523		Lim	
6	20095445	20139719		Lim	
6	24034416	24342463		Gue	
6	27927608	28277760		Gue	
6	31081921	31081921		Jer	
6	33450062	33712714		Jer	
6	33450062	33833305		Nor	
6	33642764	34076036		Ang	
6	34195111	34195111		Ang	
6	35242978	35461591		Ang	
6	37908330	37975696		Ang	
6	38774879	38774879		Ang	
6	39309298	39623155		Her	
6	41752613	42099998		Hol	
6	43893795	44068558		Ang	
6	44614641	44714358		Cha	
6	46616748	46878129		Her	
6	46820055	46878129		Lim	
6	54759464	55199755	ZT		
6	56079069	56143187		Ang	
6	61590746	61892976	ZT		
6	67190873	67190873		Lim	
6	70777638	70777638		Lim	
6	72198048	72253005		Jer	
6	77085018	77770651		Hol	
6	80251687	80338390			Guz
6	80455942	81821735			Guz
6	80574939	80789500		Gue	
6	81481074	81946116		Ang	
6	112611819	113194494		Gue	

6	118252961	118649364	ZT		
6	118281301	118353935			Guz
7	5426178	5695120		Ang	
7	8304451	8304451		Ang	
7	9003495	9182396		Ang	
7	9112088	9182396		Jer	
7	16886385	17033776		Jer	
7	17340164	17567301		Jer	
7	20289846	20289846		Nor	
7	20479996	20685141		Gue	
7	20786085	21599668		Gue	
7	21198207	21542670			Gir
7	22566709	23078152		Gue	
7	23016916	23156602		Nor	
7	39224663	39408588		Gue	
7	42319411	42319411		Nor	
7	42437898	42736530		Nor	
7	42858075	42858075		Nor	
7	42963574	43230190		Nor	
7	44034801	44108337		Hol	
7	44682616	44805462		Nor	
7	45816236	46263993		Gue	
7	47371662	47371662			Nel
7	47506357	47950533			Nel
7	47559314	47559314		Jer	
7	47950533	48078303			Gir
7	51131500	51273470		Hol	
7	51375336	52080598		Bro	
7	51375336	52080598		Hol	
7	51456410	52080598		Nor	
7	51626813	51710045		Her	
7	51783465	52080598		Ang	
7	52231298	52317683		Ang	
7	52231298	52231298		Bro	
7	52231298	52317683		Her	
7	52231298	52563489		Hol	
7	52231298	52317683		Nor	
7	52684381	52836863		Hol	
7	52836863	53402793		Gue	
7	53498236	53627815		Nor	
7	53627815	54144114		Gue	
7	54334873	54590451		Lim	
7	59180139	59704797			Gir
7	59314603	59910601		Gue	
7	63973572	64468563		Nor	
7	64685425	64764114		Nor	
7	65205183	65242121	ZT		
7	67345428	67435114		Jer	

7	68772679	68772679		Jer	
7	69919712	70148815			Nel
7	70313242	70391912			Nel
7	70935529	71042608		Cha	
7	71977836	72085410		Bro	
7	74662861	74746996		Ang	
7	76242524	76242524		Cha	
7	76352907	77027187		Cha	
7	78730794	79072441		Cha	
7	78993771	79072441		Gue	
7	79286849	79286849		Gue	
7	79286849	79286849		Lim	
7	79394912	79511994		Lim	
7	80505078	81051251		Bro	
7	81180619	81399995		Bro	
7	81422559	81550627			Nel
7	82745359	82948614		Nor	
7	87481184	87681984		Bro	
7	90378312	90534132			Nel
7	91842765	92286550		Nor	
7	95472912	95674482			Nel
7	98598188	99371157	ZT		
7	103016270	103294596			Gir
7	103457774	103499128		Nor	
7	105168965	105530475			Gir
7	110297126	110297126			Gir
7	110415789	110637456			Gir
7	110714665	110931177		Hol	
8	1317824	1572543		Gue	
8	2948943	3489893		Hol	
8	4744813	5580213		Bro	
8	9467104	9753660		Hol	
8	15446560	15612794		Ang	
8	16933256	17043052		Lim	
8	17777469	17946780		Lim	
8	20893054	20893054		Lim	
8	23344221	23619430		Ang	
8	24014551	24188422		Ang	
8	24732722	24842403		Jer	
8	24807908	24842403		Ang	
8	30585051	30644755		Gue	
8	31754646	31801262		Ang	
8	36656101	37085687		Ang	
8	36928935	37022168		Bro	
8	37491972	37771074			Gir
8	38166242	38250880			Guz
8	38166242	38206825			Nel
8	40238396	40532424		Lim	

8	43432019	43591156	Bro	
8	47115955	47403730	Nor	
8	49155564	49334308	Ang	
8	53701338	53871305	Nor	
8	54174761	54386837	Bro	
8	55649349	56283438	Bro	
8	55927704	56381952	Ang	
8	55927704	55959006	Her	
8	56322708	56452972	Her	
8	59209410	59419670		Guz
8	59759811	59819259	Bro	
8	60434020	60434020	Jer	
8	60805803	60872538	Her	
8	61741224	62123367	Gue	
8	61865662	62123367	Her	
8	62283927	62283927	Gue	
8	62817801	62888898		Nel
8	63065209	63280979		Nel
8	63072645	63273121	Gue	
8	63144428	63238470	Her	
8	63238470	63370830	Lim	
8	63320365	63708097	Ang	
8	63504584	63661095	Her	
8	68858661	69167651		Gir
8	69834960	70219718		Guz
8	71313642	71429865		Nel
8	72603211	72603211	Bro	
8	75140241	75449229	Nor	
8	76853364	77526328	Her	
8	78221423	78458014	Jer	
8	78221423	78318625		Nel
8	78453183	78453183		Nel
8	78556086	78556086		Nel
8	85262707	85546437	Bro	
8	85435042	85657033	Hol	
8	90378651	90427053	Her	
8	91924906	91924906	Her	
8	92369622	92513261		Guz
8	94358932	94358932	Her	
8	94470215	94637822	Her	
8	103695388	103741290	Lim	
8	104300617	104507052	Cha	
8	104614429	104719232	Cha	
8	104824384	104824384	Cha	
8	107733398	107830220	Jer	
8	108350843	108514141	Lim	
8	109611130	109693786	Gue	
9	1402494	1402494	Jer	

9	1503658	1587562	Jer	
9	3494268	3706545		Nel
9	3544279	3684547	Lim	
9	3796382	4191092	Lim	
9	3817160	3817160		Nel
9	3847236	3940908	Hol	
9	3926090	3926090		Nel
9	4033934	4158198		Nel
9	4305338	4362391	Lim	
9	6980134	7316340	Hol	
9	9688314	10071262	Jer	
9	10492704	10555304	Ang	
9	10761338	11187344	Hol	
9	11079124	11145913	Jer	
9	12909682	12987755	Jer	
9	19937462	19995669	Bro	
9	19995669	19995669	Jer	
9	20123664	20309620	Bro	
9	22107839	22169678		Gir
9	22232110	22452492	Hol	
9	22554584	22554584	Hol	
9	27666537	27882575	Hol	
9	33348492	33391054	Jer	
9	39183378	39451347	Cha	
9	40436120	40664307	Jer	
9	40964261	41291501	Hol	
9	41399038	41746816	Hol	
9	42556097	42767160	Ang	
9	43081332	43229106	Hol	
9	43229106	43421580	Jer	
9	43527096	43643762	Jer	
9	43563436	43877566	Ang	
9	43995195	44166269	Hol	
9	44269454	44269454	Hol	
9	45602588	45767377	Bro	
9	45784946	46299883		Guz
9	45867860	46118697	Bro	
9	47986240	48062003	Hol	
9	50586063	51244887	Lim	
9	55574798	55750201		Gir
9	55857023	56152439		Gir
9	56266678	56393910		Gir
9	56349339	56411057	Hol	
9	62015746	62153583	Lim	
9	63452753	63452753	Ang	
9	63574601	63574601	Ang	
9	63676365	63676365	Ang	
9	64800747	64905799	Bro	

9	67156161	67206285	Cha	
9	74249760	74601457		Gir
9	76300687	76346736	Cha	
9	76706088	76772631		Nel
9	84535049	84535049	Hol	
9	84901502	84973909	Jer	
9	104855944	105303067	Jer	
9	105376125	105531512		Nel
10	1506191	1927319		Nel
10	6583446	6644227	Lim	
10	21663015	21663015	Hol	
10	27828262	28125910	Her	
10	30494266	30837912	Gue	
10	30764887	30837912	Bro	
10	30837912	30837912	Jer	
10	30939474	30939474	Gue	
10	30939474	30939474	Jer	
10	31043847	31128138	Ang	
10	31043847	31330749	Gue	
10	31043847	31043847	Jer	
10	38030548	38262598	Bro	
10	38540164	38764506	Bro	
10	41329885	41479100	Hol	
10	41584872	41584872	Hol	
10	42512119	42625497	Cha	
10	44291217	44291217	Bro	
10	44942502	45084424	Nor	
10	45041722	45084424	Bro	
10	45225775	45225775	Bro	
10	45225775	45225775	Nor	
10	45346828	45846534	Bro	
10	45346828	45766891	Nor	
10	45509686	45538277	Hol	
10	48095531	48274876	Nor	
10	49896490	50595507	Bro	
10	58292569	58459047	Her	
10	58563129	58563129	Her	
10	61411012	61411012		Nel
10	61518236	61518236		Nel
10	65303032	65476239	Jer	
10	70485481	70710371	Ang	
10	70710371	70937071	Gue	
10	70710371	70937071	Nor	
10	70833294	71022679	Hol	
10	70889383	71022679	Jer	
10	70972920	71071718	Bro	
10	71045263	71045263	Ang	
10	73642900	73642900	Jer	

10	73766212	73788411			Nel
10	75550002	75645682		Nor	
10	77492297	77839926		Cha	
10	78742465	78871653		Ang	
10	78986607	78986607		Ang	
10	79110275	79240420		Ang	
10	79346962	79346962		Ang	
10	79455690	79455690		Ang	
10	79572452	79572452		Ang	
10	82814483	83061813		Her	
10	86353818	86353818		Ang	
10	86519779	86691323		Ang	
10	86592920	86592920		Hol	
10	90367300	90367300		Ang	
10	90847397	90902402		Ang	
10	91464250	91500061		Jer	
10	92073773	92073773		Ang	
10	96885456	96885456		Ang	
10	97259585	97356070		Ang	
10	103217676	103398749			Guz
10	103578088	103698520		Ang	
11	2779943	3050160		Jer	
11	11993676	13090823	ZT		
11	15790857	15790857			Nel
11	16914701	17253092	ZT		
11	17360567	17716204	ZT		
11	17796470	18117290		Her	
11	18749323	18933896			Guz
11	21281912	21628948		Hol	
11	32848500	32944087		Her	
11	34269089	34366561		Gue	
11	34474506	34620459		Gue	
11	36614495	36703565		Her	
11	41270304	41411065			Gir
11	41517065	41840880			Gir
11	42462274	42462274		Jer	
11	43782801	43782801			Nel
11	44623029	44623029		Jer	
11	44730405	44730405		Jer	
11	46152936	46752152			Guz
11	47547123	47751475		Hol	
11	49138061	49263389		Hol	
11	49513395	49718366		Hol	
11	52827981	53190924		Jer	
11	54589952	54835002		Hol	
11	55378041	55444756		Ang	
11	57077011	57122357		Nor	
11	57122357	57122357		Ang	

11	57225773	57225773		Ang	
11	57225773	57225773		Nor	
11	57352654	57612186		Ang	
11	57352654	57698139		Nor	
11	57513633	57612186		Jer	
11	57584865	57584865			Nel
11	57699190	57699190			Nel
11	58898410	58958613			Nel
11	60834800	60834800		Jer	
11	60993402	60993402		Jer	
11	61700872	61700872			Nel
11	61815063	62144546			Guz
11	61815063	62144546			Nel
11	62016830	62046170		Jer	
11	62286261	62468836			Guz
11	62286261	62286261			Nel
11	62922969	63083499			Guz
11	63083499	63083499			Nel
11	71049841	71110237		Cha	
11	72884873	73563924			Nel
11	75188485	75306027		Cha	
11	77638200	77952883		Cha	
11	82638082	83141013		Her	
11	87324940	87517044		Lim	
11	90834903	90834903		Her	
11	92760660	92861686			Nel
11	93546324	93589025		Jer	
11	94520855	94671637		Lim	
11	95851888	96109177		Ang	
11	95995955	96310082		Lim	
11	96231754	96416913		Jer	
11	96833927	97127235		Lim	
11	96856553	97165214			Nel
11	97977371	98061774			Gir
11	98007989	98248695			Nel
11	99133796	99169226		Jer	
11	102177393	102177393		Jer	
12	7398	7398	ZT		
12	855263	1185172		Hol	
12	2411488	2555233		Jer	
12	2555233	2644450		Hol	
12	2709896	2840501		Her	
12	3663510	3726470		Jer	
12	5790823	5873762		Ang	
12	9470495	9554780		Nor	
12	11413972	11465252			Gir
12	11952739	12079648		Jer	
12	12130438	12130438		Her	

12	15637232	15812507		Bro	
12	17652739	17777535		Her	
12	17821920	17821920		Nor	
12	18420344	18804912		Her	
12	21108696	21215798		Nor	
12	25821702	26728626		Nor	
12	26091552	26687038		Lim	
12	26415353	26474785		Gue	
12	26474785	26820533		Her	
12	28096664	28137445		Jer	
12	31117625	31166171			Gir
12	32310451	32519161			Guz
12	34003865	34066603		Nor	
12	34186079	34186079		Nor	
12	35071458	35071458		Her	
12	35505910	35760005		Nor	
12	35860041	36004714		Nor	
12	36034539	36034539			Guz
12	36327358	36580366			Guz
12	37594269	37748400			Nel
12	37871194	38222071			Nel
12	38351681	38351681			Nel
12	38541351	38541351			Nel
12	38708042	38708042			Nel
12	38811401	38811401			Nel
12	41831612	42072404		Her	
12	46692520	46692520			Guz
12	47545150	47647948		Lim	
12	52032001	52082569		Jer	
12	56924171	57455552			Guz
12	57094160	57094160			Nel
12	57556609	57687027			Guz
12	61782214	61782214		Gue	
12	70094561	70339877	ZT		
12	70512846	70512846	ZT		
12	70776064	70776064	ZT		
12	71453071	71453071	ZT		
12	72167314	72167314	ZT		
12	72272866	72495671	ZT		
12	73745994	73745994	ZT		
12	75254804	75330903	ZT		
12	76710313	76785743	ZT		
12	76791869	76881130		Nor	
12	80341071	80425016		Gue	
13	3623556	3862870		Gue	
13	11862167	11995413		Cha	
13	12626523	12626523		Her	
13	18962684	19024912		Bro	

13	19004088	19201759	Her	
13	23767720	24003859	Bro	
13	26937127	26937127	Jer	
13	27525398	28216775	Cha	
13	34712301	34758845		Nel
13	35474541	35781279		Nel
13	36231773	36367627		Nel
13	39167826	39280645	Hol	
13	42441984	42441984	Hol	
13	43033627	43033627	Gue	
13	43122300	43546670	Bro	
13	46817491	47103769		Gir
13	49406229	49503637	Bro	
13	50607191	50607191	Jer	
13	50760200	50760200		Gir
13	50869402	51028725		Gir
13	54668408	54668408		Gir
13	55309919	55888683	Her	
13	73752118	73752118	Nor	
13	74455449	74455449		Nel
13	77996845	78056241		Nel
14	10350252	10396044	Her	
14	11554484	11554484	Nor	
14	11782284	12070404		Guz
14	12189249	12398318		Guz
14	12727219	13036902	Jer	
14	24763610	24763610	Her	
14	24869608	24939285	Her	
14	26053531	26775878	Her	
14	28062900	28159943	Ang	
14	33192707	33303060	Bro	
14	34280875	34371583	Lim	
14	35801257	36075593	Ang	
14	39067537	39067537	Ang	
14	39184898	39581240	Ang	
14	39658425	39884259	Her	
14	40502641	40502641	Lim	
14	41954479	42187575	Bro	
14	42187575	42472189	Jer	
14	46359424	46456216	Bro	
14	46359424	46751517	Lim	
14	49540290	49604994	Lim	
14	49612207	49612207		Nel
14	49760299	49809387	Lim	
14	49766006	49955911		Nel
14	50075470	50219196		Nel
14	50219196	50219196		Guz
14	51324497	51347339	Jer	

14	52989072	54035518			Guz
14	53150040	53609912		Her	
14	53193111	53609912		Gue	
14	53332825	53438913			Nel
14	53550213	54035518	ZT		
14	53683729	53872420		Jer	
14	54175673	54300664			Guz
14	54175673	54231380	ZT		
14	57167775	57217596		Lim	
14	57678310	57888506		Ang	
14	64451563	64504478		Jer	
14	64451563	64757034			Gir
14	66332394	66549938		Jer	
14	68588028	68820639		Hol	
14	72531775	72675508		Her	
14	73231262	73583478		Her	
14	73755259	74135137		Her	
15	541451	684098		Hol	
15	607318	675523			Gir
15	4266078	4266078		Lim	
15	5286282	5342417		Nor	
15	10303535	10360514			Nel
15	10481071	10481071			Nel
15	11505690	11685734		Cha	
15	14562911	14562911		Hol	
15	17810751	17810751		Hol	
15	27505692	27548079		Ang	
15	28573894	28670202		Lim	
15	30605734	30605734		Jer	
15	35017416	35293898		Her	
15	35664204	36080303		Her	
15	36080303	36157938			Guz
15	36983921	37085321		Nor	
15	41223589	41285121		Her	
15	41367360	41498723			Guz
15	43000037	43039782		Lim	
15	43613588	44076774		Lim	
15	43644653	43644653			Nel
15	43779412	43912400			Nel
15	43884531	43996164		Jer	
15	48782120	48822979		Cha	
15	48861147	49064593			Guz
15	49245028	49245028			Guz
15	65071060	65270348		Gue	
15	69157600	69445029			Guz
16	3970030	4027681			Nel
16	4235903	4598916		Her	
16	12347163	12472290		Her	

16	14277442	14329324		Jer	
16	16039261	17069240	ZT		
16	19740336	20450779	ZT		
16	24776941	24845497		Jer	
16	30107699	30107699		Hol	
16	31019604	31139383			Gir
16	31473763	31548783		Nor	
16	34638116	34968754		Nor	
16	35049210	35634059		Ang	
16	35220072	35220072		Nor	
16	36476830	37151556	ZT		
16	37180652	37298418			Gir
16	37194331	37254143		Lim	
16	41368131	41453104		Gue	
16	41572983	41572983		Gue	
16	41678990	41775870		Gue	
16	41700411	41790638			Gir
16	41888513	42364249		Gue	
16	41899936	42048595			Gir
16	42537236	43096999		Jer	
16	42608418	43529259		Ang	
16	43272235	43623611		Gue	
16	44269629	44644017			Gir
16	44863309	44863309			Guz
16	45473156	45630316		Gue	
16	45473156	45630316		Hol	
16	45549919	45549919		Lim	
16	45736614	45736614		Gue	
16	45736614	45984474		Hol	
16	46085920	46141853		Hol	
16	46592707	46950070		Jer	
16	46677636	46724139		Lim	
16	47172573	47258906		Gue	
16	47507964	47570993			Gir
16	48258144	48421449		Gue	
16	48619950	48884638		Gue	
16	48711908	48867982		Lim	
16	49863171	49978311		Gue	
16	50535583	50783764		Lim	
16	51305656	51973649			Gir
16	52023890	52161691		Jer	
16	54046062	54301432		Nor	
16	57111792	57174124			Gir
16	57465584	57556128		Lim	
16	58190532	58471410		Bro	
16	58272188	58400017		Hol	
16	58359610	58837961		Nor	
16	58938382	59182205		Nor	

16	60430367	60470851		Lim	
16	61757564	61980466		Jer	
16	65313199	65313199		Nor	
16	72778008	72778008			Gir
16	76224768	76384441		Nor	
16	77946034	78278222		Nor	
16	77961709	78059640		Jer	
17	4791944	4791944		Hol	
17	4791944	4967167		Nor	
17	8512165	8575700	ZT		
17	22354814	22354814		Jer	
17	27654318	27732351			Nel
17	37763279	37822104		Her	
17	40004303	40004303		Jer	
17	58814396	58814396		Nor	
17	65096190	65236429		Nor	
17	66907233	66975172		Jer	
17	68032148	68069561		Ang	
17	69765254	69870054		Ang	
17	73166013	73559752		Her	
17	74674649	74769219		Her	
17	74898756	74898756		Her	
17	75044721	75044721		Her	
18	6169342	6190185		Her	
18	15724957	15724957		Ang	
18	18167040	18347077		Hol	
18	24421799	24770950			Guz
18	28035367	28265343		Lim	
18	29435311	29898412		Lim	
18	30759728	30784902			Nel
18	30918548	30918548			Nel
18	32039478	32197423		Jer	
18	33851145	34129031		Lim	
18	35033416	35187164		Jer	
18	40446288	40789159		Gue	
18	43109494	43510794			Nel
18	47444620	47613065			Nel
18	48040506	48227582			Guz
18	52288303	52429256			Nel
18	52701819	52764540			Gir
18	52900975	53320100			Gir
18	53439714	53537165			Gir
18	53638416	53638416			Gir
18	53753661	53915419			Gir
18	54394911	54573177			Guz
18	54704168	54704168			Guz
18	65911355	65985963		Hol	
19	262485	262485		Hol	

19	413710	413710	Hol	
19	10671018	10712603	Jer	
19	17759190	17777692	Jer	
19	19675737	19781067	Hol	
19	19970122	20088486	Hol	
19	20830901	20830901		Nel
19	26060515	26087727	Bro	
19	28277521	28312344	Gue	
19	28507797	28799133		Guz
19	34016467	34081031	Her	
19	34144101	34461281	Hol	
19	35049942	35222595	Jer	
19	44551095	44689047		Guz
19	44799390	44891730		Guz
19	47407572	47407572	Ang	
19	51796076	51796076	Lim	
20	3637677	4174779		Gir
20	4278244	4656931		Gir
20	8898757	8943192		Guz
20	11122063	11254640		Guz
20	14182164	14416570	Hol	
20	16402419	16690385	Gue	
20	20301638	20357937	Jer	
20	20396411	20595893	Lim	
20	27507810	27507810		Guz
20	30674472	30825893	Nor	
20	30928803	30928803	Nor	
20	31029524	31029524	Nor	
20	31433472	31674164	Nor	
20	31891078	32014836	Her	
20	32498665	32498665	Nor	
20	32633053	32874023	Nor	
20	33446648	33537819	Hol	
20	33490278	33537819	Gue	
20	34022009	34474873	Lim	
20	34414759	34414759	Gue	
20	36017483	36416315	Nor	
20	36324969	36375373	Lim	
20	41389949	41580673	Bro	
20	56689509	56718200	Lim	
20	58525877	58587805		Nel
20	58692179	58692179	Lim	
20	60612128	61616232	Her	
20	61984638	62036376	Lim	
20	62003414	62055493	Hol	
20	70734693	70772846		Gir
20	70875830	71231949		Gir
20	71280563	71922138	Jer	

20	71834868	71922138	Her	
21	8725	8725	Her	
21	8725	8725	Hol	
21	8725	8725	Jer	
21	204222	204222	Her	
21	204222	204222	Jer	
21	204222	204222	Nor	
21	361318	361318	Gue	
21	361318	361318	Her	
21	361318	361318	Jer	
21	361318	361318	Nor	
21	504994	504994	Gue	
21	504994	504994	Her	
21	504994	504994	Jer	
21	504994	504994	Nor	
21	645376	645376	Her	
21	645376	645376	Jer	
21	645376	645376	Nor	
21	774574	774574	Her	
21	774574	774574	Nor	
21	3561585	3653561	Her	
21	3762262	4308308	Her	
21	4411538	4411538	Her	
21	5900058	5971363	Nor	
21	6919205	7096541	Nor	
21	7049776	7173321	Bro	
21	8458546	8541959	Bro	
21	10503457	10677966	Ang	
21	13884396	13933435	Hol	
21	14288138	14766834	Bro	
21	14714185	14818540	Ang	
21	14957433	15127073		Gir
21	16237693	16237693	Jer	
21	16785248	16863723	Jer	
21	16863723	16979042	Gue	
21	18706352	18762361		Nel
21	19600565	19752873	Bro	
21	19799240	19949845	Hol	
21	22323904	22461853	Lim	
21	22323904	22639853	Nor	
21	22976563	23238454	Bro	
21	24136096	24539045	Bro	
21	24437617	24486885		Nel
21	24643755	24643755	Bro	
21	25974504	26020647	Jer	
21	29624611	29911323	Bro	
21	30874989	31032359	Gue	
21	31707051	31764616	Bro	

21	31873638	32830315		Bro	
21	34498778	34667353		Nor	
21	41059072	41128918			Nel
21	43118544	43924035		Hol	
21	43487945	43566426			Nel
21	44055266	44055266		Hol	
21	44748769	44901335			Gir
21	45234173	45435950		Lim	
21	45600155	45882200			Gir
21	45882200	45882200			Nel
21	47727421	47857951		Jer	
21	47749318	48177959		Cha	
21	48487618	48834422		Jer	
21	49143093	49178133			Guz
21	59060143	59199306		Ang	
21	60262849	60293487			Nel
21	64250367	64412250		Gue	
21	67218872	67760359			Guz
21	68673095	68868534			Guz
21	68987919	68987919			Guz
21	69852429	70230072	ZT		
22	638872	803110		Hol	
22	1504583	1504583	ZT		
22	1623884	1623884	ZT		
22	11402326	11402326		Gue	
22	11649808	11763239		Lim	
22	14103245	14186222		Lim	
22	16608311	16840205			Gir
22	19877407	20310645		Bro	
22	22669066	23049830		Cha	
22	23613510	23636256		Bro	
22	24966558	25284841		Her	
22	27869508	27944643		Hol	
22	28052191	28110766		Hol	
22	28725064	28875324		Gue	
22	34370024	34664272		Cha	
22	34784761	34986631		Cha	
22	40654899	40711031		Gue	
22	42030577	42250591		Cha	
22	42599407	42647970		Cha	
22	42749647	43246293		Cha	
22	48211935	48272599		Bro	
22	48866088	48866088			Guz
22	50199885	50474049		Hol	
22	50349312	50432123		Nor	
22	50637321	50809843		Her	
22	50919853	51471443		Her	
22	55958425	56128888		Cha	

22	57153731	57284409		Cha	
22	61312492	61312492			Gir
23	10121	10121			Gir
23	139285	139285			Gir
23	301633	301633			Gir
23	428745	503014			Gir
23	732008	844112		Hol	
23	5609998	5657236			Guz
23	5753208	5753208			Nel
23	5861179	5861179			Nel
23	9661692	9852730			Nel
23	10858794	11094059			Nel
23	11566720	11889590			Guz
23	11999280	12062277			Guz
23	12177758	12384489		Hol	
23	13997275	14111515			Guz
23	18964952	19215320		Bro	
23	24242547	25248392	ZT		
23	24392637	24939249		Cha	
23	25449663	25574353	ZT		
23	25859762	25859762	ZT		
23	26711242	27665155	ZT		
23	27776739	28435313	ZT		
23	28571770	28957304	ZT		
23	28952482	29024901		Lim	
23	29078825	29675638	ZT		
23	29148829	29304816		Lim	
23	29831845	31194961	ZT		
23	30015495	30939191		Ang	
23	30163726	30446125		Lim	
23	30777345	30977659		Her	
23	31895767	31978264			Nel
23	32608468	33237258	ZT		
23	32973497	33308775		Cha	
23	36707756	36787506			Nel
23	40660798	40684631		Hol	
24	154231	154231		Gue	
24	439276	439276		Gue	
24	595437	918703		Gue	
24	643511	809504		Hol	
24	9076993	9208956		Nor	
24	11671411	12035684		Cha	
24	14120858	14120858			Guz
24	14222578	14437894			Guz
24	20313385	20364536			Nel
24	20477264	20797489			Nel
24	20572058	20928280			Gir
24	21269616	21432906		Cha	

24	24925791	25369456		Guz
24	25475807	25751740		Guz
24	25751740	25968172	Gue	
24	25820839	25874297	Jer	
24	30934156	30978136	Cha	
24	31650946	31869672	Ang	
24	42101894	42101894		Nel
24	42232650	42500735		Nel
24	42623754	42689753		Nel
24	46653518	46761421	Ang	
24	48203225	48630311	Nor	
24	60807235	60879053	Lim	
24	61185652	61423980	Lim	
24	61892167	61892167	Lim	
24	61968531	62004962		Nel
24	62182152	62261855	Lim	
24	62580288	62580288	Cha	
25	3405842	3465726	Jer	
25	4248368	4309238	Jer	
25	6091348	6091348		Nel
25	8152490	8371682	Cha	
25	10628650	10628650		Guz
25	10735504	10735504		Guz
25	12252170	12303617		Guz
25	13405791	13571593	Lim	
25	21583665	21666132	Her	
25	22370742	22514409	Lim	
25	22812917	23081592		Guz
25	22969428	23104348		Nel
25	23210540	23210540		Nel
25	24671209	24836724		Nel
25	29690882	29690882	Her	
25	37676059	37867138		Nel
25	40674489	40714999		Nel
26	3586610	3586610	Jer	
26	4783644	4783644	Jer	
26	4891430	5141217		Nel
26	6161663	6619517	Bro	
26	10339734	10538894		Gir
26	22288463	22288463	Lim	
26	22390438	22390438	Lim	
26	22501867	22558692	Lim	
26	25585089	25641218	Lim	
26	26411617	26433407	Her	
26	30218417	30251629		Nel
26	30900459	30954376		Nel
26	39027844	39217448	Cha	
26	39331767	39706525	Cha	

26	39748287	39780004		Jer	
26	40698893	40733253		Cha	
26	42953022	43226269		Cha	
26	45786773	45786773		Jer	
26	46348285	46403318		Her	
26	46663802	47234109	ZT		
26	46685978	47706414		Cha	
26	51117232	51358645		Cha	
27	3314120	3486378		Cha	
27	3596438	3625884			Nel
27	6901933	7002026		Hol	
27	14898244	15044426		Hol	
27	18008818	18008818		Ang	
27	18108993	18265385		Ang	
27	18402344	18402344		Ang	
27	18506025	18506025		Ang	
27	18663778	18692965		Hol	
27	18947023	18947023			Nel
27	19508946	19749943		Her	
27	19861211	20138705		Her	
27	20372030	20504395		Ang	
27	24615175	24709555		Ang	
27	30006323	30006323		Lim	
27	31259957	31462506		Lim	
27	32813604	32986416		Gue	
27	34692151	34777867			Nel
27	36000462	36208029			Nel
28	471788	471788			Guz
28	999093	1148363		Her	
28	3424089	3720220		Ang	
28	6730869	6730869		Nor	
28	7051126	7186023		Bro	
28	9046486	9160211		Lim	
28	9695595	9852321		Gue	
28	19371256	19932570		Her	
28	25949221	26144471		Jer	
28	30113999	30207706			Nel
28	35113955	35216536		Her	
28	35362739	35566652		Cha	
28	35362739	35566652		Her	
28	35692135	35945282		Cha	
28	35692135	35778958		Her	
28	37238317	37394869		Cha	
28	40102528	40102528		Jer	
28	40920166	40964508		Ang	
29	8136855	8196100		Bro	
29	11803793	11984964		Lim	
29	19170744	19287565		Her	

29	19992231	19992231	Cha	
29	25144872	25202432	Her	
29	26751704	26827353		Guz
29	28041906	28087498		Gir
29	29219049	29547625		Gir
29	30100272	30201805	Hol	
29	30405864	30472536	Hol	
29	40823987	41149594	Nor	
29	41745163	41841952	Bro	
29	42677448	43098226	Nor	
29	46514979	46607724	Jer	
29	46776296	46834374		Gir
29	48006213	48100160		Guz
29	48963532	49024179		Guz
29	49132446	49132446		Guz
